# Identification of Prognostic miRNA Signature and Lymph Node Metastasis-Related Key Genes in Cervical Cancer

**DOI:** 10.3389/fphar.2020.00544

**Published:** 2020-05-08

**Authors:** Shuoling Chen, Chang Gao, Yangyuan Wu, Zunnan Huang

**Affiliations:** ^1^Key Laboratory of Big Data Mining and Precision Drug Design of Guangdong Medical University, Research Platform Service Management Center, Guangdong Medical University, Dongguan, China; ^2^The Second School of Clinical Medicine, Guangdong Medical University, Dongguan, China; ^3^Key Laboratory for Research and Development of Natural Drugs of Guangdong Province, School of Pharmacy, Guangdong Medical University, Dongguan, China; ^4^Institute of Marine Biomedical Research, Guangdong Medical University, Zhanjiang, China

**Keywords:** cervical cancer, miRNA, key gene, prognostic signature, lymph node metastasis

## Abstract

**Background:**

miRNAs and genes can serve as biomarkers for the prognosis and therapy of cervical tumors whose metastasis into lymph nodes is closely associated with disease progression and poor prognosis.

**Methods:**

R software and Bioconductor packages were employed to identify differentially expressed miRNAs (DEMs) from The Cancer Genome Atlas (TCGA) database. GEO2R detected differentially expressed genes (DEGs) in the GSE7410 dataset originating from the Gene Expression Omnibus (GEO). A Cox proportional hazard regression model was established to select prognostic miRNA biomarkers. Online tools such as TargetScan and miRDB predicted target genes, and overlapping DEGs and target genes were defined as consensus genes. Kyoto Encyclopedia of Genes and Genomes (KEGG) pathway enrichment and Gene Ontology (GO) function annotations were performed to discern the potential functions of consensus genes. STRING and Cytoscape screened key genes and constructed a regulatory network.

**Results:**

A combination of four miRNAs (down-regulated miR-502 and miR-145, up-regulated miR-142 and miR-33b) was identified as an independent prognostic signature of cervical cancer. A total of 94 consensus genes were significantly enriched in 7 KEGG pathways and 19 GO function annotations including the cAMP signaling pathway, the plasma membrane, integral components of the plasma membrane, cell adhesion, etc. The module analysis suggested that *CXCL12*, *IGF1*, *PTPRC CDH5*, *RAD51B*, *REV3L*, and *WDHD1* are key genes that significantly correlate with cervical cancer lymph node metastasis.

**Conclusions:**

This study demonstrates that a four-miRNA signature can be a prognostic biomarker, and seven key genes are significantly associated with lymph node metastasis in cervical cancer patients. These miRNAs and key genes have the potential to be therapeutic targets for cervical cancer. Among them, two miRNAs (miR-502 and miR-33b) and two key genes (*PTPRC* and *CDH5*) were first reported to be potential novel biomarkers for cervical cancer. The current study further characterizes the progression of lymph node metastasis and mechanism of cervical tumors; therefore, it provides a novel diagnostic indicator and therapeutic targets for future clinical treatments.

## Introduction

Worldwide, cervical cancer is the fourth most common female malignancy with an extremely high morbidity and mortality rate (Cohen et al., 2019). According to the World Health Organization (WHO), there were 569,874 new cases of cervical tumors in 2018, accounting for 3.2% of all new cancer cases in that same year (Bray et al., 2018). Most cervical tumors are attributed to recurrent HPV infections. Aberrant expression of oncoproteins encoded by HPV genetic material, such as E6 and E7, partially leads to epigenetic instability, which affects the carcinogenesis and metastasis of cervical cancer (Doorbar, 2006).

MicroRNAs (miRNAs) are associated with the development of a wide range of cancers, including cervical cancer (Park et al., 2017). miRNAs are a group of non-coding, single-stranded RNA molecules that are approximately 22 nucleotides (nt) in length and encoded by endogenous genes (Bartel, 2004; Chen and Kang, 2015). Abnormally expressed miRNAs regulate various biological processes such as apoptosis, proliferation, and metabolism (Zhang et al., 2007; Lee and Dutta, 2009). Moreover, miRNA dysregulation plays a critical role in cancer progression and metastasis in multiple cancers, including cervical cancer (Gómez-Gómez et al., 2013; Huang et al., 2017). For example, Yao et al. (Yao et al., 2018) demonstrated that decreased expression of HPGD by miR-146b-3p induced proliferation, migration, and anchorage-independent growth of cervical cancer cells by activating the STAT3 and AKT signaling pathways. This indicated that miRNAs may be clinically applicable as potential biomarkers and therapeutic targets.

Tumor metastasis is the leading cause of death for most cervical cancer patients. It generally involves complex processes such as extracellular matrix degradation, lymphangiogenesis, angiogenesis, clonal growth at secondary sites, etc. (Zhang et al., 2015). The metastasis and invasion of tumor cells through blood and lymph nodes are crucial processes in the progression of cervical cancer (Dai et al., 2017). Newly formed lymphatic vessels are comprised of endothelial cells that are not tightly connected. Therefore, cervical cancer cells can easily invade this endothelial layer and metastasize to lymph nodes (Zhang et al., 2015). In addition, the poor prognosis of cervical cancer patients is correlated with high invasiveness and diffuse lymph node metastasis (Wang et al., 2016). Lymph node metastasis is the main metastatic pathway and the most critical factor in the prognosis and recurrence of cervical cancer cases (Huang and Fang, 2018). However, a detailed understanding of the specific signatures and molecular mechanisms of lymph node metastasis is lacking. Therefore, the search for a reliable lymphatic signature is important in determining malignant metastasis, and it also provides information that can guide the clinical treatment of cervical tumor patients.

Many have proposed conducting a large-scale systematic analysis of miRNAs, genes, and clinical data using bioinformatics to further characterize the functions of miRNAs and genes in certain disease states, clarify their potential as disease-related signatures, and discover new disease biomarkers and drug targets (Liang et al., 2017). Researchers can obtain tumor data from public databases and conduct differential expression, survival, and prognosis analyses as well as target gene prediction and functional characterization using R language (Sepulveda, 2020), TargetScan (Agarwal et al., 2015), miRDB (Wong and Wang, 2015), DAVID (Huang et al., 2009), etc. With these tools, tumor biomarkers can be screened, and their mechanism of action can be further elucidated. For example, Liang et al. (Liang et al., 2017) identified a three-miRNA signature (miR-145, miR-200c, and miR-218-1) that is a prognostic factor of cervical tumors by conducting Cox univariate and multivariate analyses on differentially expressed miRNAs screened from clinical samples in TCGA database.

In this study, we conducted a multi-step analysis using various R language packages on clinical samples downloaded from the TCGA (Tomczak et al., 2015) and GEO (Barrett et al., 2007) databases to identify DEMs and DEGs. A Cox proportional hazard regression model was then established to determine potential prognostic biomarkers from the available DEMs. Subsequently, the target genes of the miRNAs biomarkers were predicted by the TargetScan and miRDB online tools and the consensus genes were further determined based on overlap between DEGs and these target genes. Finally, MCODE (Bader and Hogue, 2003) software in Cytoscape (Shannon et al., 2003) was used to identify key genes related to lymph node metastasis caused by early-stage cervical tumors. Together, the prognostic miRNAs and key genes mined in this study will provide new insights in elucidating the molecular mechanisms of cervical cancer and contribute to finding new therapeutic targets and prognostic biomarkers for cervical cancer patients.

## Materials and Methods

### Data Preparation and Differential Expression Analysis

The expression profiles and clinical data for 310 samples were obtained from TCGA (https://portal.gdc.cancer.gov/) database on July 27, 2018. These samples included 307 primary solid cervical cancer tissue samples and three normal tissue samples. Even though this study used many more cancer samples than normal samples, previous studies by Yang et al. (2019), Zeng et al. (2018), etc. have demonstrated that appropriate prognostic models with diagnostic indicators and clinically significant therapeutic targets can be obtained with similar unbalanced sample sets. DEMs were analyzed using the edgeR (Robinson et al., 2010), gplots (Li S. et al., 2020), and limma (Ritchie et al., 2015) R language packages (Version 3.5.1) according to the screening criteria of |log2FC| > 1 and *P_adj_* < 0.05. The GSE7410 expression profile data from 24 samples were obtained from the GEO (https://www.ncbi.nlm.nih.gov/geo/) database. The samples included 19 early-stage cervical tumor tissues with lymph node metastasis and five healthy cervical tissues. DEGs were analyzed using GEO2R based on the screening criteria of |log2FC| > 1 and *P_adj_* < 0.05.

### Establishment of a Cox Proportional Hazard Regression Model

The Cox proportional hazard regression model (George et al., 2014) was established to analyze the association between DEMs and overall survival. The survival package was used in the univariate and multivariate Cox analyses of DEMs (Liang et al., 2017). miRNAs with *P* < 0.05 as calculated by the univariate Cox analysis were considered strongly correlated with overall patient survival. The multivariate Cox analysis used stepwise regression to screen a prognostic model based on the Akaike information criterion (AIC) value (Vrieze, 2012), where the model with the smallest AIC value contains the smallest number of miRNAs that best predict cervical cancer patient prognosis. miRNAs with *P* < 0.05 as calculated by the multivariate Cox analysis were considered as independent prognostic factors.

### Prognostic Model Construction

Using the results of the multivariate Cox analysis, the risk score was calculated as follows: Risk Score = Exp (miRNA_1_) **×** β_1_ **+** Exp (miRNA_2_) **×** β_2_ **+… +** Exp (miRNA_n_) **×** β_n_. The patients were divided into low- and high-risk groups based on their median risk score. A risk score curve was plotted to demonstrate the risk score differences according to the model. A survival status map was plotted to demonstrate the survival status of each sample. A heatmap was plotted to demonstrate the expression level differences of the four prognostic miRNAs in the low- and high-risk groups. A survival curve was plotted to demonstrate the 5-year survival in high- and low-risk groups. And an ROC curve of the model was constructed to determine its predictive ability. A model with an AUC value larger than 0.7 possesses a strong prediction function.

### Target Gene Prediction

TargetScan and miRDB predicted the target genes of miRNAs obtained from the multivariate Cox analysis. Target genes that were predicted by both tools were considered target genes of those miRNAs. And the consensus genes were further obtained from the overlap between DEGs related to lymph node metastasis and candidate target genes from the prognosis miRNAs.

### KEGG Pathways and GO Function Annotation Enrichment Analysis

To further understand the underlying biological significance of DEMs and DEGs, DAVID (https://david.ncifcrf.gov/) produced KEGG pathway (Kanehisa and Goto, 2000) enrichment and GO functional annotations (Ashburner et al., 2000) of the consensus genes using *P* < 0.05 as a demarcation criterion. The pathways or annotations with the smallest *P*-value or the largest count value were considered as key KEGG pathways and GO function annotations. GO function annotations include three parts: biological process (BP), cellular component (CC), and molecular function (MF).

### PPI Network Construction and Module Analysis

STRING (http://string-db.org) (Szklarczyk et al., 2017) analyzed the consensus genes and obtained protein interaction data. Proteins with a minimum required interaction score greater than or equal to 0.400 were selected to construct the PPI (protein-protein interaction) network, and nodes with network interruption were hidden. The combined score was imported into Cytoscape software (Version 3.7.1, https://cytoscape.org/). The Molecular Complex Detection (MCODE) plug-in in Cytoscape calculated the MCODE score and selected the significant modules of key genes using the screening criteria of Degree Cut-off = 2, Haircut on, Node Score Cut-off = 0.2, k-core = 2, and Max. Depth = 100. Moreover, the logFC value of genes in the interaction network was also imported into Cytoscape to show the up/down regulation status.

### Network Visualization

Regulatory relationship data were imported into Cytoscape along with miRNAs, key genes, consensus genes, key KEGG pathways, and GO functional annotations. The visualization network was performed with Cytoscape to explore the potential regulatory relationship among them.

### Analysis Procedure

**Figure 1:**
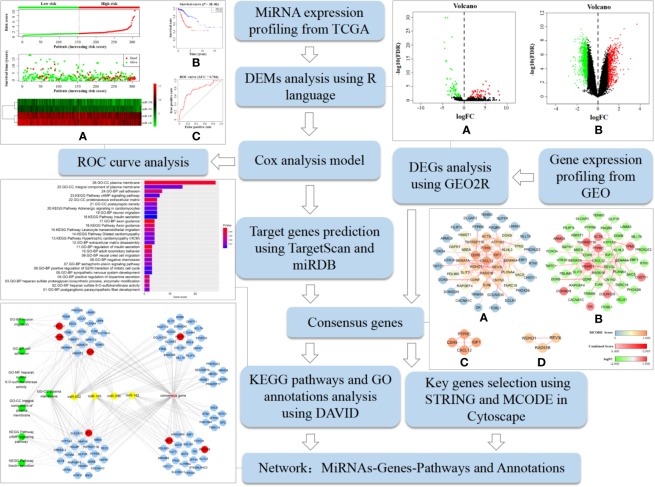
Analysis procedure of the data mining process used to screen tumor biomarkers and key genes in this study. It includes specific bioinformatics methods, data processing tools, and partial research results.

**Figure 1** shows the analysis procedure of the data mining processes that was used to screen tumor biomarkers and key genes in this study. It was based on the extensive use of R language and various online analytical tools. We first obtained DEMs and DEGs using R language and GEO2R, respectively, by analyzing cervical cancer-related miRNAs and gene expression profiles downloaded from TCGA and GEO databases. A prognostic model was constructed *via* Cox analysis to detect key miRNAs to form a best-available prognostic signature whose target genes were predicted by TargetScan and miRDB. Subsequently, we performed KEGG pathway and GO annotation analyses using DAVID to clarify the function of the consensus genes from the overlap between DEGs and the predicted target genes of key miRNAs. Furthermore, STRING and MCODE selected key genes among consensus genes. Finally, we constructed a miRNAs-Genes-Pathways and Annotations network to elucidate potential regulatory mechanisms. The approach described here is a feasible protocol to identify potential tumor biomarkers and key genes.

## Results

### miRNA and Gene Differential Expression Analyses

**Figure 2:**
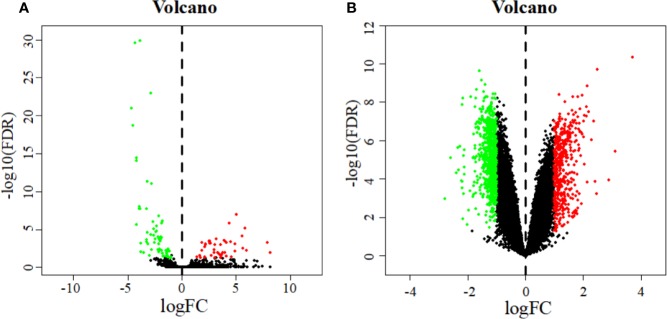
Volcano plot of DEMs **(A)** and DEGs **(B)**. The abscissa represents the log2 transformation value of the differential expression fold change between the cervical cancer samples and the normal samples. The larger the | logFC | value is, the greater the fold change is. The ordinate represents the -log10 transformation value of the FDR value. The larger the -log10 transformation value is, the more significant the difference is. Green dots represent significantly down-regulated miRNAs or genes. Red dots represent significantly up-regulated miRNAs or genes.

A total of 110 DEMs were obtained after analyzing miRNA expression profiles from TCGA with R language using *P*_adj_ < 0.05 and |log2FC| > 1 as screening criteria. Among them, 64 miRNAs were significantly down-regulated, and 46 miRNAs were significantly up-regulated (**Table S1**). At the same time, 1840 DEGs related to early cervical cancer lymph node metastasis were identified by analyzing the GSE7410 expression profile with GEO2R using *P*_adj_ < 0.05 and |log2FC| > 1 as screening criteria. Among them, 1,298 genes were significantly down-regulated, and 542 genes were significantly up-regulated (**Table S2**). The volcano map illustrates the significant differences and distribution of the fold change in DEMs and DEGs (**Figure 2**).

### miRNA-Based Signature Identification as a Prognostic Biomarker

A total of 15 miRNAs related to patient survival were obtained from 110 DEMs using univariate Cox analysis (*P* < 0.05) (**Table 1**). Four miRNAs related to patient prognosis (miR-502, miR-142, miR-145, and miR-33b) were further screened from the above 15 miRNAs by multivariate Cox analysis (**Table 2**). Among them, miR-502 and miR-145 were down-regulated, and miR-142 and miR-33b were up-regulated in cervical cancer tissues. The multivariate Cox analysis demonstrated that these four miRNAs could be used as independent prognostic factors in cervical cancer (*P* < 0.05).

**Table 1:** Univariate analysis of cervical cancer patients.

miRNA	HR	z	*P*
**miR-502**	0.47295993	−4.710714692	2.47E-06
**miR-142**	0.726460896	−3.311319458	0.000928571
miR-362	0.634317782	−3.089446858	0.002005296
miR-101-2	0.70026668	−2.832903633	0.004612729
miR-101-1	0.699830285	−2.831809241	0.004628545
**miR-145**	0.753658772	−2.628062686	0.008587269
miR-1468	0.71581814	−2.570507809	0.010154954
miR-204	0.852831645	−2.377156425	0.017446688
miR-140	0.633673302	−2.368455333	0.017862537
**miR-33b**	0.803090114	−2.212258218	0.026948828
miR-126	0.700379333	−2.186430538	0.028784121
miR-218-1	0.815625887	−2.121987729	0.033838769
miR-504	0.757023197	−2.003615093	0.045111308
miR-99a	0.879776376	−1.986775064	0.046947329
miR-331	0.691475709	−1.965107538	0.049401792

Bolded miRNAs are the prognostic miRNAs.

**Table 2:** Multivariate analysis of cervical cancer patients.

	Coef	Exp (Coef)	Se (Coef)	z	*P*
**miR-502**	−0.676	0.509	0.156	−4.33	1.50E-05
**miR-142**	−0.297	0.743	0.106	−2.82	0.0049
**miR-145**	−0.29	0.748	0.101	−2.87	0.0042
**miR-33b**	−0.209	0.811	0.101	−2.07	0.0382

### Application of a Cox Proportional Hazard Regression Model in Disease Prognosis

**Figure 3:**
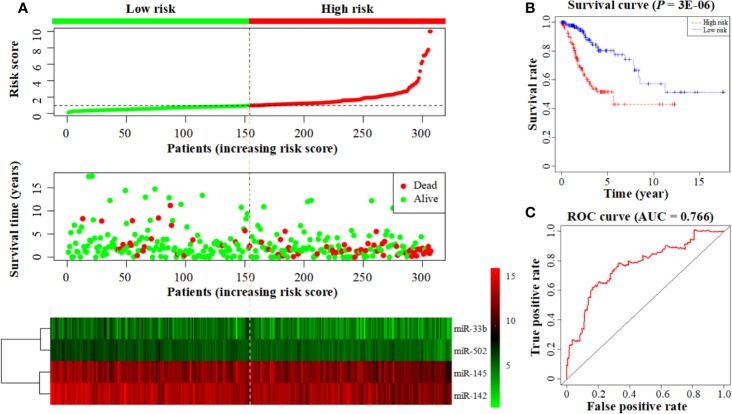
Construction of a prognostic model based on a 4-miRNA signature. **(A)** From top to bottom: the risk score curve, survival status map, and expression heatmap between the low- and high-risk groups. The color bar shows the relative miRNA expression value with red indicating high expression and green indicating low expression. **(B)** Survival curve for the low- and high-risk groups. **(C)** The ROC curve for survival predictions.

The prognostic model consisting of four miRNAs was constructed by multivariate cox analysis. The formula of the model was defined as follows: Risk score = (Exp (miR-502) × (−0.676) + Exp (miR-145) × (−0.290) + Exp (miR-142) × (−0.297) + Exp (miR-33b) × (−0.209)). The survival risk score based on the four miRNAs was calculated by the model formula and divided patients into low- (154 samples) and high-risk (153 samples) groups according to their median risk score. The risk score curve demonstrates that the risk score of individuals in the low-risk group is small and consistent while the risk score of individuals in the high-risk group is larger and rises significantly (**Figure 3A** top). The survival status map demonstrates that individuals in the low-risk group have low mortality rates, and individuals in the high-risk group have a short survival time (most survived for less than 5 years) (**Figure 3A** middle). The heatmap demonstrates that low miRNA expression levels are associated with high-risk scores, and high miRNA expression levels are associated with low-risk scores (**Figure 3A** bottom). These all demonstrate the reliability of the prognostic model in classifying high- and low-risk groups. And the survival curve demonstrates that the survival rate in the low-risk group is notably higher than in the high-risk group (*P* = 3E-06; low-risk group: 80.3%, 95% CI = 71.3–90.4%; high-risk group: 51.6%; 95% CI = 41.6–64.0%) (**Figure 3B**). The time-dependent ROC curve analysis indicates that this prognostic model has a high level of credibility, sensitivity, and specificity with an AUC value of 0.766 (**Figure 3C**).

### Target Gene Prediction for the Four Prognostic miRNAs

The prediction results combined with TargetScan and miRDB identified 267 target genes of miR-502, 423 target genes of miR-145, 370 target genes of miR-142, and 279 target genes of miR-33b (**Figure S1**). After excluding 111 repetitive target genes co-regulated by multiple miRNAs, 1,228 target genes of the four prognostic miRNAs were obtained. Moreover, 94 consensus genes (**Table 3**) were identified from the overlap between 1840 DEGs (**Table S3**) and 1228 target genes (**Table S4**).

**Table 3:** Consensus genes of four prognostic miRNAs discovered in the overlap between predicted target genes and DEGs related to cervical cancer lymph node metastasis.

miRNA	Consensus Genes				
**miR-502**	ATP1A2	DLGAP2	FILIP1L	C12orf54	TLR6
	DAPK1	DCLK1	DONSON	SLIT3	WDHD1
	FBN2	DCUN1D5	CHST11	SLC7A14	LAMA3
	BCL7A	CDH5	ZNF471	CNTN2	PLXNA4
	COL10A1	DOK6	GLP1R	PGM5	SYNPO2
	EML6	MLLT6	ACSS3	HS6ST2	NUDT10
	RCC2	PHOX2B	SAMD4B		
**miR-142**	GDNF	ZFPM2	TRIM36	NBEA	IGF1
	FOXO4	FAM199X	HDLBP	TMTC1	GK
	BNC2	REV3L	SLC2A13	PDLIM5	SP2
	TNRC18	SACS	ST6GALNAC3	EGLN3	RTN1
**miR-145**	MMP16	ITGBL1	SEMA6A	ACTB	HS6ST1
	REV3L	ZFP14	SLC38A11	TNFRSF11B	PAQR9
	ST6GALNAC3	TUFT1	ARHGAP6	CREB3L2	GATC
	HTRA1	KLHL3	TPM3	KCNA6	GXYLT1
	EBF1	HTR1F	RAD51B	ZRANB3	SLITRK4
	RAPGEF4	DGKB			
**miR-33b**	LRP8	PRAMEF17	GPR173	SEMA7A	MMP16
	ATP1A2	TENM3	CACNA1C	DSC3	SECISBP2L
	HMGB3	PTPRB	ABHD2	GDNF	PTPRC
	CMTR2	CXCL12	PRICKLE2	ARMC8	

### KEGG Pathway and GO Function Annotation Details of Consensus Genes

**Figure 4:**
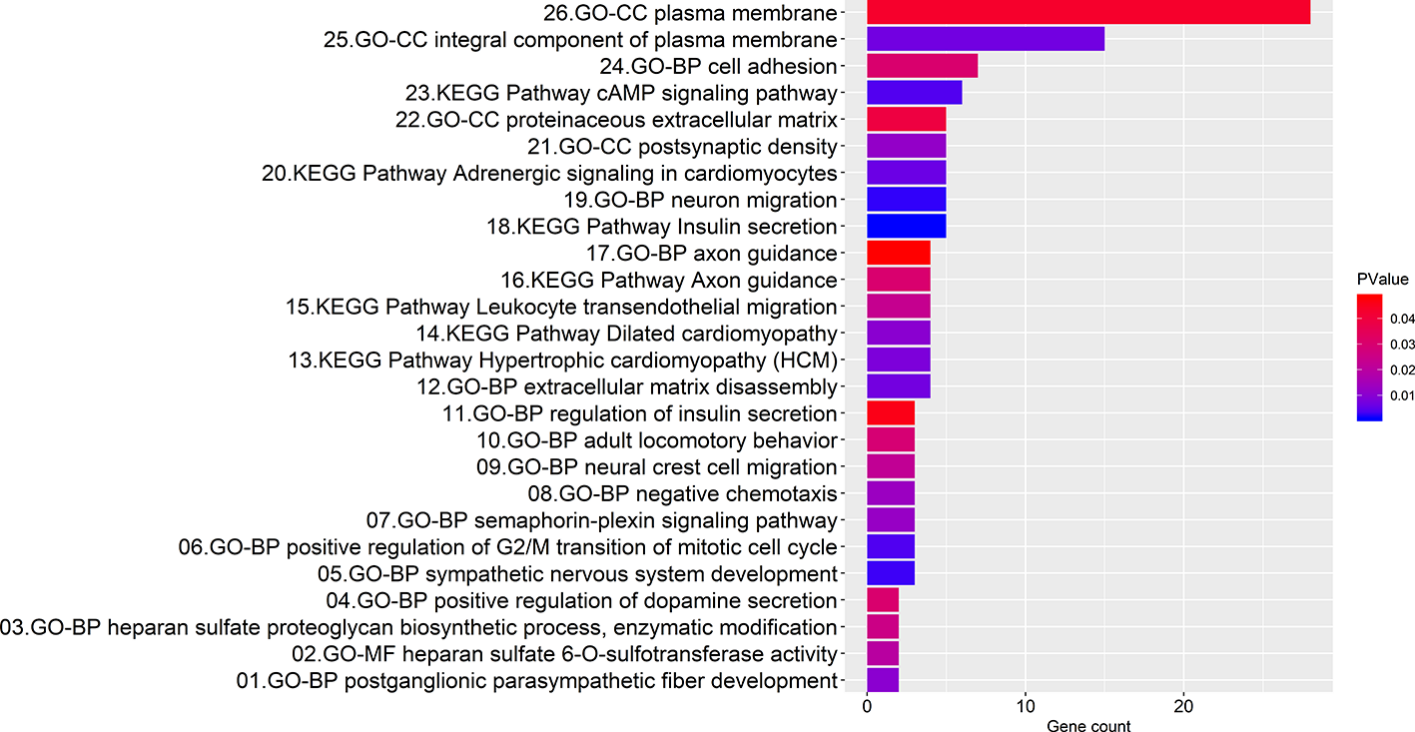
Bar graph illustrating the enrichment analysis. The abscissa represents the number of consensus genes involved in KEGG pathways or GO function annotations. The ordinate represents items of the primary KEGG pathways or GO function annotations.

Enrichment analyses produced 7 KEGG pathways and 19 GO function annotations from the consensus genes using the screening criteria of *P* < 0.05 (**Figure 4**). Consensus genes enriched in each pathway or function annotations were showed in **Table S5**. KEGG pathways involving insulin secretion, cAMP signaling, adrenergic signaling in cardiomyocytes, leukocyte transendothelial migration, etc. were mainly enriched. Among them, the insulin secretion pathway had the smallest *P*-value, and this pathway is associated with five consensus genes (*P* = 1.05E-03). The cAMP signaling pathway had the largest number (count = 6) of involved consensus genes, and its *P*-value was 3.87E-03. BP GO annotations involved in neuron migration, positive regulation of the G2/M mitotic cell cycle transition, extracellular matrix disassembly, cell adhesion, etc. were mainly enriched. The annotation with the smallest *P*-value in the BP involved neuron migration (*P* = 2.07E-03), and it correlated with five consensus genes. The largest number of related consensus genes involved cell adhesion (count = 7), and its *P*-value was 3.03E-02. And GO annotations in CC involving the plasma membrane, actin cytoskeleton, postsynaptic density, etc. were mainly enriched. The annotation with the smallest *P*-value in the CC was an integral component of the plasma membrane (*P* = 6.92E-03), and it involved 15 consensus genes. The largest number of linked consensus genes involved the plasma membrane (count = 28), and its *P*-value was 4.33E-02. Furthermore, GO annotations in MF involving heparan sulfate 6-O-sulfotransferase activity were mainly enriched and had a *P*-value of 1.98E-02 and two associated consensus genes.

### Identification of Key Modules Using a Cluster Analysis of Protein-Protein Interactions

**Figure 5:**
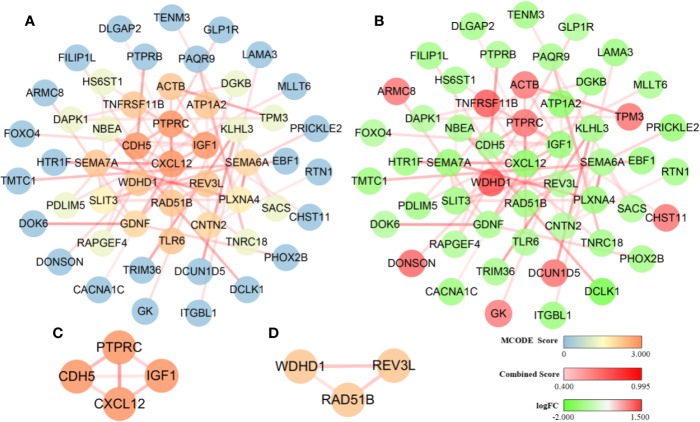
Diagram of the protein-protein interaction network. The dark and light shading of the lines between solid circles indicates high and low interaction relationships, respectively, which is represented by the Combined Score. **(A)** The color of solid circles representing protein targets is distinguished by the MCODE score value. The blue circles are protein targets with an MCODE score value of 0, and these proteins did not participate in the construction of the key module. The dark and light orange circles are protein targets with a high MCODE score value of 3.000 and 2.000, respectively, and these proteins participated in the construction of the key module. **(B)** The color of solid circles representing protein targets is distinguished by the logFC value. The green circles are protein targets with a low logFC value and represent significantly down-regulated genes. The red circles are protein targets with a high logFC value and represent significantly up-regulated genes. **(C)** A module was constructed using the four protein targets with MCODE score values of 3.000. **(D)** A module was constructed using the three protein targets with MCODE score values of 2.000.

The interaction network presents protein-protein interactions, the strength of the interactions within the protein modules (**Figure 5A**), and up/down regulation conditions of the involved genes (**Figure 5B**). It consists of 52 nodes and 59 edges. Two important modules with MCODE scores greater than 2.0 were screened by MCODE and included seven key genes: *CXCL12*, *IGF1*, *PTPRC*, and *CDH5* (**Figure 5C**); *RAD51B*, *REV3L*, and *WDHD1* (**Figure 5D**). Among them, *CXCL12*, *IGF1*, *CDH5*, *RAD51B*, and *REV3L* were significantly down-regulated while *WDHD1* and *PTPRC* were notably up-regulated. It is worth to note that we also performed topology parameters analysis of PPI and obtained the same seven key genes above (**Figure S2**). The readers who are interested in this process can read the supplementary TopologyParametersAnalysis.doc.

### Visualization Network of MiRNAs-Genes-Pathways and Annotations

**Figure 6:**
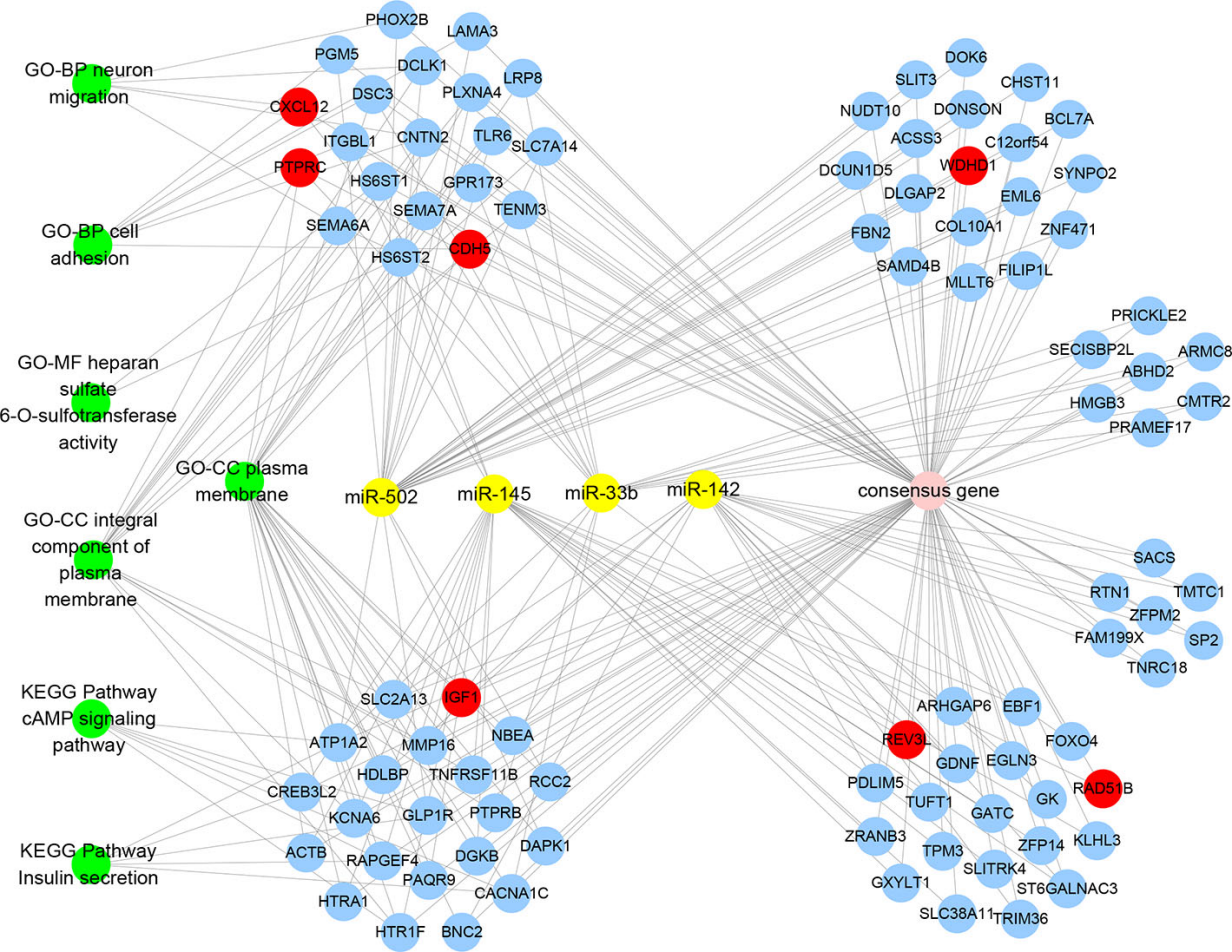
MiRNAs-Genes-Pathways and Annotations visualization network denoting the relationships between miRNAs, consensus genes, key genes, key KEGG pathways, and GO functional annotations. Yellow solid circles represent miRNAs, blue solid circles represent consensus genes, red solid circles represent key genes, and green solid circles represent KEGG pathways and GO functional annotations.

The visualization network demonstrated that seven key genes are regulated by four miRNAs and are involved in seven key KEGG pathways and GO functional annotations (**Figure 6**). *WDHD1* and *CDH5* are regulated by miR-502; *RAD51B* and *REV3L* are regulated by miR-145; *REV3L* and *IGF1* are regulated by miR-142; and *CXCL12* and *PTPRC* are regulated by miR-33b. The enrichment analysis of the consensus genes showed that there was no key gene enrichment in the key KEGG pathways, while *CXCL12*, *IGF1*, *CDH5*, and *PTPRC* were involved in the key GO functional annotations. Specifically, *CXCL12* was enriched in neuron migration and cell adhesion of BP. *CDH5* was enriched in cell adhesion of BP. *IGF1*, together with *CDH5* and *PTPRC*, were enriched in the plasma membrane of CC. *PTPRC* was enriched in the integral component of the plasma membrane of CC.

## Discussion

Cervical carcinoma is one of the most common malignancies in females. Approximately 90% of cervical cancers occur in low- and middle-income countries. Lymph node metastasis and recurrence are the main manifestations in cervical cancer patients with a poor prognosis (Cohen et al., 2019). miRNAs collectively regulate thousands of human cancer-related, protein-coding genes and regulate many important biological processes that facilitate cancer development (i.e. cell growth, invasion, and apoptosis) (Lu et al., 2005). Therefore, miRNAs have become a hotspot of tumor research in the past decade. To identify novel and reliable prognostic biomarkers and important regulatory genes related to cervical cancer and lymph node metastasis, this study first identified 110 DEMs and 1840 DEGs, separately, from expression profiling and clinical data uploaded into TCGA and GEO databases, respectively. Next, a cancer prognosis model based on four prognostic miRNAs (miR-502, miR-145, miR-142, and miR-33b) was established using univariate Cox, multivariate Cox, and survival analyses. Subsequently, 1,228 target genes were predicted using TargetScan and miRDB, and 94 consensus genes were obtained from the overlap of DEGs and predicted target genes. Finally, seven key genes related to lymph node metastasis (i.e. *CXCL12*, *IGF1*, *PTPRC*, *CDH5*, *RAD51B*, *REV3L*, and *WDHD1*) were identified using STRING and Cytoscape.

According to the prognostic model, the expression levels of miR-502 and miR-145 were down-regulated, and the expression levels of miR-142 and miR-33b were up-regulated in cervical cancer. Among these four miRNAs, miR-145 and miR-142 have been reported that they were related to cervical cancer in previous experimental studies.

Previous studies have shown that the down-regulation of miR-145 is closely related to cervical cancer and its lymph node metastasis. Azizmohammadi et al. (2017) used qRT-PCR and a multivariate Cox analysis to demonstrate that the expression of miR-145 is reduced in cervical cancer tissues. They also showed that reduced expression of miR-145 is related to lymph node metastasis (*P* = 0.02), advanced Federation International of Gynecology and Obstetrics (FIGO) stage (*P* = 0.007), and vascular invasion (*P* = 0.026), which confirms miR-145’s potential as a prognostic biomarker for the early detection of cervical cancer (Azizmohammadi et al., 2017). Ma et al. (2019) demonstrated that miR-145 is also down-regulated in cervical tumor cells, and up-regulation of miR-145 reduces cell proliferation by directly suppressing its target gene, FSCN1. Shi et al. (2012) also showed miR-145 is decreased in cervical cancer cells, and increasing miR-145 expression enhances chemo-sensitivity and inhibits invasion and migration by enhancing p53. The above experimental results are consistent with our prediction that the expression of miR-145 is suppressed in cervical cancer cells.

While our prognostic model predicted that miR-142 expression is increased, existing experimental studies illustrated that miR-142 expression is decreased in cervical cancer cells. Li et al. (2017) revealed that when compared with normal tissue, miR-142 expression is lower in cervical cancer cells and correlates with a poor prognosis. Deng et al. (2015) demonstrated that miR-142-3p is down-regulated in cervical tumors. The overregulation of miR-142-3p inhibits the expression of its target gene, *FZD7*, and further halts the proliferation and invasion of cervical cancer (Deng et al., 2015). Xia et al. (2018) reported that Metformin, an anti-cancer drug, up-regulates miR-142-3p expression in cervical cancer cells. They also showed that Metformin inhibits the invasion and migration of tumor cells by decreasing the sponge effect of MALAT1, up-regulating the expression of miR-142, and down-regulating the expression of the target gene, *HMGA2* (Xia et al., 2018). These three studies showed that miR-142 is a tumor suppressor gene. Thus, the decreased expression of miR-142 in cervical cancer cells is contrary to our results from the data mining calculation that shows an increase in its expression. We do not know the reason(s) for this contradiction, but it should be clarified in the future.

Although no experiment has demonstrated a correlation between miR-502 and miR-33b in cervical cancer, studies have shown that the down-regulation of miR-502 and up-regulation of miR-33b are involved in other types of cancer. Sun et al. (2014) demonstrated that miR-502, which is down-regulated in breast cancer cells, suppresses early apoptosis by targeting TRAF2 and restraining the NF-κB signaling pathway. Furthermore, Li et al. (2009) demonstrated that the activation of NF-κB results in a lower tumor grade, larger tumor volume, higher invasiveness, and increased metastasis in cervical cancer tissues. Therefore, miR-502 may affect cervical cancer lymph node metastasis by participating in the NF-κB signaling pathway. Zhang et al. (2019) showed that the up-regulation of miR-33b inhibits the Wnt/β-catenin signaling pathway by decreasing ZEB1 expression and promoting endometriosis. Although endometriosis is a common and benign disease, it has similar characteristics to malignancies including cell proliferation, invasion, metastasis, and recurrence. Also, Ramachandran et al. (2012) showed that abnormal activation of the Wnt/β-catenin pathway is common in cervical tumors, which may enhance proliferation and prevent apoptosis of cervical cancer cells. Therefore, up-regulation of miR-33b may affect the proliferation and apoptosis of cervical cancer cells by inhibiting the Wnt/β-catenin signaling pathway. To summarize, miR-502 and miR-33b might be involved in cervical cancer formation through distinctive ways as explained above, but such speculation needs to be experimentally validated.

To date, one computational paper exploring the relationship between cervical cancer and the expression of miRNA through data mining has been published. Liang et al. (2017) constructed a three-miRNA signature containing miR-145, miR-200c, and miR-218-1 by processing miRNA data from TCGA database. Their study demonstrated that the expression of miR-145 is significantly decreased in cervical tumor tissues. Furthermore, they found that the expression levels of miR-142 and miR-33b are up-regulated (Liang et al., 2017). This study provided results consistent with ours regarding the expression levels of miR-145, miR-142, and miR-33b. It is worth noting that although the expression of miR-142 was experimentally shown to be down-regulated in cervical cancer cells, as we mentioned previously, this earlier computational study circumstantially confirms the results of our research.

According to our predictions, among the seven key genes involved in early-stage cervical cancer lymph node metastasis, *CXCL12*, *IGF1*, *RAD51B*, *REV3L*, and *CDH5* are down-regulated, and *WDHD1* and *PTPRC* are up-regulated. **Table 4** compares the expression of these seven key genes in cervical cancer, other cancers, and lymph node metastasis as reported in previous experimental studies and our calculated results. The decreased expression of *CXCL12* in cervical cancer cells and its role in lymph node metastasis was confirmed in previous experiments (Yadav et al., 2016), and the decreased expression of *IGF1* and the increased expression of *WDHD1* were also experimentally validated in cervical cancer cells but only in lymph node metastasis of other cancers (Serrano et al., 2006; Kümmel et al., 2007; Huang et al., 2008; Zhou et al., 2016; Liu et al., 2019). Interestingly, the expression of *RAD51B* is also decreased in cervical cancer cells, but experiments showed that its expression is increased in other cancer lymph node metastases (Cheng et al., 2016; Hang et al., 2016). In addition, *REV3L* expression is increased in cervical cancer cells and lymph node metastases of other cancers in the laboratory, which is inconsistent with our prediction results (Yang et al., 2015; Zhu et al., 2016). Finally, *PTPRC* and *CDH5* have not been previously reported to be associated with cervical cancer or its lymph node metastasis. While experiments demonstrate that *PTPRC* expression is increased in other cancers and their lymph node metastases (Collette et al., 2007; Camacho et al., 2018), *CDH5* is up-regulated in other cancers but down-regulated in lymph node metastasis of colorectal cancer (Tacconi et al., 2015; Hung et al., 2016; Higuchi et al., 2017).

**Table 4:** The expression of key genes reported from previous experimental studies.

	CXCL12	IGF1	WDHD1	RAD51B	REV3L	PTPRC	CDH5
Cancer	▼	▼	▲	▼	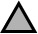	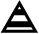	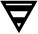 △
LNM	▼ 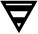	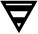	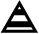	△	△	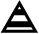	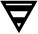

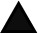
 up-regulated in cervical cancer, agreed with our calculated result.

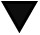
 down-regulated in cervical cancer, agreed with our calculated result.

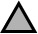
 up-regulated in cervical cancer, disagreed with our calculated result.

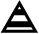
 up-regulated in other cancers, agreed with our calculated result in cervical cancer.

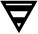
 down-regulated in other cancers, agreed with our calculated result in cervical cancer.

△ up-regulated in other cancers, disagreed with our calculated result in cervical cancer.

*CXCL12* is the ligand for the G-protein coupled receptor-like chemokine (C-X-C motif) receptors 4 and 7. It affects many cellular processes such as immune monitoring, inflammatory response, tumor growth, and metastasis (Colamussi et al., 2001; Yadav et al., 2016). Yadav et al. (2016) demonstrated that *CXCL12* expression is absent in cervical cancer. They also illustrated that *CXCL12* silencing enables cells to evade apoptosis and leads to the progression and metastasis of cervical cancer (Yadav et al., 2016). Meanwhile, Müller et al. (2001) reported that *CXCR4* is usually highly expressed in tumor tissues, and the *CXCR4*/*CXCL12* axis could prevent breast tumor lymph-node metastasis and lung metastasis when *CXCR4* is neutralized. Thus, *CXCL12* can act as a tumor suppressor in lymph node metastasis of cervical and other cancers, and it might inhibit tumor progression through the *CXCR4*/*CXCL12* axis.

*IGF1* is a cytokine that mediates cell growth and development (Macaulay, 1992). Serrano et al. (2006) reported low serum *IGF1* expression in cervical cancer cells, and reduced expression of *IGF1* is associated with an increased risk of cervical cancer. Huang et al. (2008) showed that lower levels of *IGF1* can effectively predict survival in patients with cervical cancer. Furthermore, they found that a combination of increased carcinoembryonic antigen (CEA) levels and decreased *IGF1* levels is significantly associated with an increased risk of death and could accurately predict patients with a poor prognosis. Though no experiment is available to confirm that *IGF1* is involved in cervical cancer lymph node metastasis, Kümmel et al. (2007) reported that in breast tumor patients, plasma *IGF1* expression is increased after dose-intensified chemotherapy, and they showed a more prominent increase in *IGF1* expression in patients with positive lymph nodes than other patients. *WDHD1*, also known as *AND1*, is involved in signal transduction, pre-mRNA processing, replication, transcription, cytoskeleton assembly, chromosome assembly, etc. (Park et al., 2012). Zhou et al. (2016) showed that *WDHD1* is up-regulated in primary human keratinocytes and spontaneously immortalized human foreskin keratinocytes cells expressing oncogene E7 in HPV-induced carcinogenesis. They also showed that *WDHD1* can increase E7-induced G1 checkpoint abolition and duplication. They further demonstrated that the polyploidy ratio of cells expressing E7 is significantly reduced after knocking down *WDHD1* with siRNA (Zhou et al., 2016). Although the role of *WDHD1* in cervical cancer lymph node metastasis has not been explained, Liu et al. (Liu et al., 2019) found that miR-494 can inhibit EMT and lymph node metastasis of cholangiocarcinoma (CCA) cells by targeting overexpressed *WDHD1*. In summary, *IGF1* and *WDHD1* are likely connected with cervical cancer lymph node metastasis.

*RAD51B* is one of the *RAD51* gene families involved in homologous recombination-mediated DNA repair (Thacker, 2005; Nagathihalli and Nagaraju, 2011). Hang et al. (2016) demonstrated that *RAD51B* is a tumor suppressor gene in cervical cancer cells, which is consistent with our prediction. They further showed that the miRNA binding sites of *RAD51B* genetic variants in cervical cancer cells may increase tumor susceptibility, and *RAD51B* is vital in gauging the cervical cancer risk of individuals and improving the effectiveness of preventive intervention (Hang et al., 2016). Currently, there are no reports that demonstrate *RAD51B* affects cervical cancer lymph node metastasis, but Cheng et al. (2016) showed that the overexpression of *RAD51B* in gastric cancer cells is significantly associated with lymph node metastasis (*P* = 0.001), advanced stage (*P* = 0.009), and invasive differentiation (*P* = 0.022), and it may act as a potential signature for early detection and poor prognosis. *RAD51B* expression in gastric cancer with lymph node metastasis is contrary to our prediction: it tended to represent the particularity of *RAD51B* expression in cervical cancer lymph node metastasis. The specific reasons for this contradiction need to be further clarified.

*REV3L* encodes the protein representing the catalytic sub-unit of Polζ, and inhibiting *REV3L* expression enables cancer cells to tolerate DNA damage and stunted growth (Knobel et al., 2011). Yang et al. (2015) showed that overexpression of *REV3L* promotes proliferation and colony formation and inhibits cervical cancer cell sensitivity to cisplatin. Thus, *REV3L* could be a potential therapeutic target for cervical cancer treatment. Zhu et al. (2016) also showed that *REV3L* is significantly up-regulated in esophageal squamous cell carcinoma tissues, and it positively correlates with lymph node metastasis (*P <*0.05) and clinical stage (*P* < 0.05). Additionally, overexpression of *REV3L* increases the expression levels of cyclin D1 and survivin, which work together to promote the growth and invasion of esophageal cancer cells (Zhu et al., 2016). These results demonstrate that *REV3L* is closely linked to cervical cancer lymph node metastasis. However, these *REV3L* experimental results are inconsistent with the inferences obtained from our data mining analysis. The specific reasons behind this contradiction need to be clarified.

*PTPRC*, also known as *CD45*, is a key regulator of cell growth, differentiation, mitosis, and carcinogenic transformation (Rheinländer et al., 2018). Camacho et al. (2018) showed that *PTPRC* is significantly overexpressed in head and neck squamous cell cancer cells, and tumor samples overexpressing *PTPRC* have significantly higher tumor-infiltrating lymphocytes (TIL) scores than tumor samples expressing low levels of *PTPRC*, leading to a poorer prognosis. Collette et al. (2007) noted that the expression of *PTPRC* plays a critical role in determining the signal transduction and proliferation response of human myeloma cells to growth factors such as IL-6 and IGF1. IL-6 and IGF1 separately induced CD45+ and CD45- myeloma cell colony formation through the MAPK/ERK signaling pathway in which CD45 is critical for myeloma proliferation (Collette et al., 2007). In addition, many experimental studies reported that the MAPK/ERK signaling pathway is crucial in cervical cancer formation. For example, Li et al. (2018) demonstrated that MAPK/ERK signaling pathway activation promotes cervical cancer cell proliferation. Tao et al. (2018) illustrated that miR-497 acts as a tumor suppressor by blocking the MAPK/ERK signaling pathway in cervical cancer cells. Furthermore, Wang et al. (2020) showed that the anti-cancer drug sclareol targets the MAPK/ERK signaling pathway and induces cervical cancer cell apoptosis and cell cycle arrest. Therefore, considering these observations, we can speculate that *PTPRC* and *IGF1* may negatively regulate the occurrence and development of cervical cancer and its lymph node metastasis by affecting the MAPK/ERK signaling pathway.

*CDH5*, also known as VE-cadherin, represses endothelial cell apoptosis and participates in endothelial cell growth contact inhibition (Cavallaro et al., 2006). Abnormal *CDH5* was found in cancer cells. Hung et al. (2016) and Higuchi et al. (2017) demonstrated that *CDH5* is over-expressed in lung cancer and gastric cancer, respectively. However, Tacconi et al. (2015) demonstrated that *CDH5* is down-regulated in colorectal cancer, and is negatively associated with its lymph node metastasis. They found that up-regulated *VEGFC*/*VEGFR3* reduces *CDH5* expression, enhances permeability, and increases trans-endothelial migration. Thus, it promotes lymphatic vessel density and colorectal cancer lymph node metastasis (Tacconi et al., 2015). Furthermore, Chaudary et al. (2011) demonstrated that suppressing the over-expression of *VEGFC*/*VEGFR3* in cervical cancer cells inhibits proliferation and cervical cancer lymph node metastasis. We suspect that the over-expression of *VEGFC*/*VEGFR3* restrains *CDH5* in cervical cancer cells and further promotes lymph node metastasis. But this assumption needs to be further studied.

**Table 3** shows that eight targeting relationships are available between four prognosis miRNAs and seven key genes. Specifically, miR-502 targets *WDHD1* and *CDH5*, miR-145 targets *REV3L* and *RAD51B*, miR-142 targets *REV3L* and *IGF1*, and miR-33b targets *CXCL12* and *PTPRC*. Among these relationships, miR-145-REV3L and miR-142-IGF1 have been reported previously. Chen et al. (2019) demonstrated that miR-145 can directly regulate the expression of *REV3L* in esophageal squamous cell carcinoma (ESCC) cells using a dual luciferase reporter assay and Western blot analysis. Also, low levels of miR-145 augmented *REV3L* mRNA and protein expression in ESCC. Xiong et al. (2018) showed that eight out of 12 target gene prediction programs including TargetScan and miRDB predicted the miR-142-IGF1 relationship. Subsequently, they suggested that decitabine exerts its therapeutic effect on hepatocellular carcinoma by inhibiting miR-142 DNA methylation, which enhances miRNA expression and further down-regulates the target genes of miR-142-5p such as *IGF1* (Xiong et al., 2018). To summarize, the above studies demonstrated target/regulatory relationships between miR-145-REV3L and miR-142-IGF1. The remaining six regulatory relationships have yet to be reported, which might lay the foundation for future research on cervical and other cancers with lymph node metastasis.

In the key KEGG pathways and GO functional annotations of key gene enrichment, it had been reported that cell adhesion and integral component of the plasma membrane were closely related to tumor proliferation and metastasis. For example, Mierke (2008) showed that the integrity of the endothelial barrier is preserved by a complex balance of cell adhesion factors. Once the integrity is compromised, tumor cells can metastasize through blood vessels or lymphatic channels (Mierke, 2008). More importantly, Zheng et al. (2017) suggested that the *CXCR4*/*CXCL12* axis plays a role in reducing the adhesion ability of colon cancer cells by regulating the Akt and IGF1R signaling pathways. Therefore, the key gene *CXCL12*, enriched in cell adhesion, may lead to cervical cancer lymph node metastasis by affecting cell-cell adhesion. In addition, protein components of the cell membrane are involved in cell signal transduction, cell interaction, and other important steps in the process of cancer cell metastasis (Lund et al., 2009). Most of the key molecules involved in the signaling pathway are distributed in the cell membrane. *PTPRC*, as a transmembrane tyrosine phosphatase, is expressed in all leukocytes, and it is involved in lymphocyte immunity against tumor cells (Tchilian et al., 2001). Therefore, we speculate that *PTPRC*, which is enriched in an integral component of the plasma membrane, may be an important signal molecule in cervical cancer metastasis. Moreover, it may be involved in a new approach to studying the mechanism of cervical cancer lymph node metastasis.

## Conclusion

By data-mining differentially expressed miRNAs and genes along with other clinical information in a multi-step analysis, we obtained a prognostic model of cervical cancer with lymph node metastasis containing four miRNAs and seven genes. We showed that four miRNAs (miR-502, miR-145, miR-142, and miR-33b) are independent and common prognostic biomarkers for patients with cervical cancer, and seven proteins (*CXCL12*, *IGF1*, *PTPRC CDH5*, *RAD51B*, *REV3L*, and *WDHD1*) are key genes significantly related to lymph node metastasis. Among them, miR-145, miR-142, *CXCL12*, *IGF1*, and *WDHD1* have been confirmed, while miR-502, miR-33b, *PTPRC*, and *CDH5* are reported, here, for the first time. Also, the expression levels and/or roles of miR-142, miR-33b, *RAD51B*, *REV3L*, and *CDH5* in cervical cancer lymph node metastasis need further clarification. In summary, our study may improve our understanding of the progression and lymph node metastasis mechanism of cervical cancer, and, therefore, provide a novel diagnostic indicator and therapeutic targets for future clinical treatments.

## Data Availability Statement

Publicly available datasets were analyzed in this study. These data can be found here: https://portal.gdc.cancer.gov/, https://www.ncbi.nlm.nih.gov/geo/query/acc.cgi?acc=GSE7410.

## Author Contributions

Conceptualization: SC, CG, and ZH. Methodology: SC, CG, and YW. Software: SC, CG, and YW. Validation: SC, YW, and ZH. Formal analysis: SC, CG, and ZH. Investigation: SC and YW. Data curation: SC and CG. Writing—original draft preparation: SC and CG. Writing—review and editing: ZH. Visualization: SC and CG. Supervision: ZH. Project administration: ZH. Funding acquisition: ZH. All authors have read and agreed to the published version of the manuscript.

## Funding

This work was supported by the National Natural Science Foundation of China (31770774), the Provincial Major Project of Basic or Applied Research in Natural Science, Guangdong Provincial, Education Department (2016KZDXM038) and the higher education reform project of Guangdong Province (2019268).

## Conflict of Interest

The authors declare that the research was conducted in the absence of any commercial or financial relationships that could be construed as a potential conflict of interest.

## Abbreviations

AUC, area under the curve; BP, biological process; CC, cellular component; CDH5, cadherin 5; CEA, carcinoembryonic antigen; CI, confidence interval; Coef, partial regression coefficient; CXCL12, C-X-C motif chemokine ligand 12; CXCR4, C-X-C motif chemokine receptor 4; CXCR7, C-X-C motif chemokine receptor 7; DAVID, Database for Annotation, Visualization, and Integrated Discovery; DEGs, differentially expressed genes; DEMs, differentially expressed miRNAs; EMT, epithelial-mesenchymal transition; Exp (Coef), relative risk; FDR, false discovery rate; GDF15, growth differentiation factor 15; GEO, Gene Expression Omnibus; GO, gene ontology; HMGA2, high-mobility group AT-hook 2; HR, hazard ratio; IGF1, insulin like growth factor 1; IGF1R, insulin like growth factor 1 receptor; KEGG, Kyoto Encyclopedia of Genes and Genome; LNM, lymph node metastasis; MALAT1, metastasis-associated lung adenocarcinoma transcript-1; MCODE, Molecular Complex Detection; MF, molecular function; Pol ζ, DNA translation synthesis polymerase ζ; PPI, protein-protein interaction; PTPRC, protein tyrosine phosphatase receptor type C; qRT-PCR, quantitative real-time polymerase chain reaction (reverse transcription-quantitative polymerase chain reaction (RT-qPCR)); RAD51B, RAD51 paralog B; REV3L, recovery protein 3 like; Se (Coef), standard error of regression coefficient; siRNA, small interfering RNA; STRING, Search Tool for the Retrieval of Interacting Genes; TCGA, The Cancer Genome Atlas; TNM, tumor node metastasis; WDHD1, WD repeat and HMG-box DNA binding protein 1.
