# Supplementary figures and images for: Identification of Prognostic miRNA Signature and Lymph Node Metastasis-Related Key Genes in Cervical Cancer
Source: Front Pharmacol. 2020 May 8;11:544. doi: 10.3389/fphar.2020.00544 (PMC7226536; doi:10.3389/fphar.2020.00544)

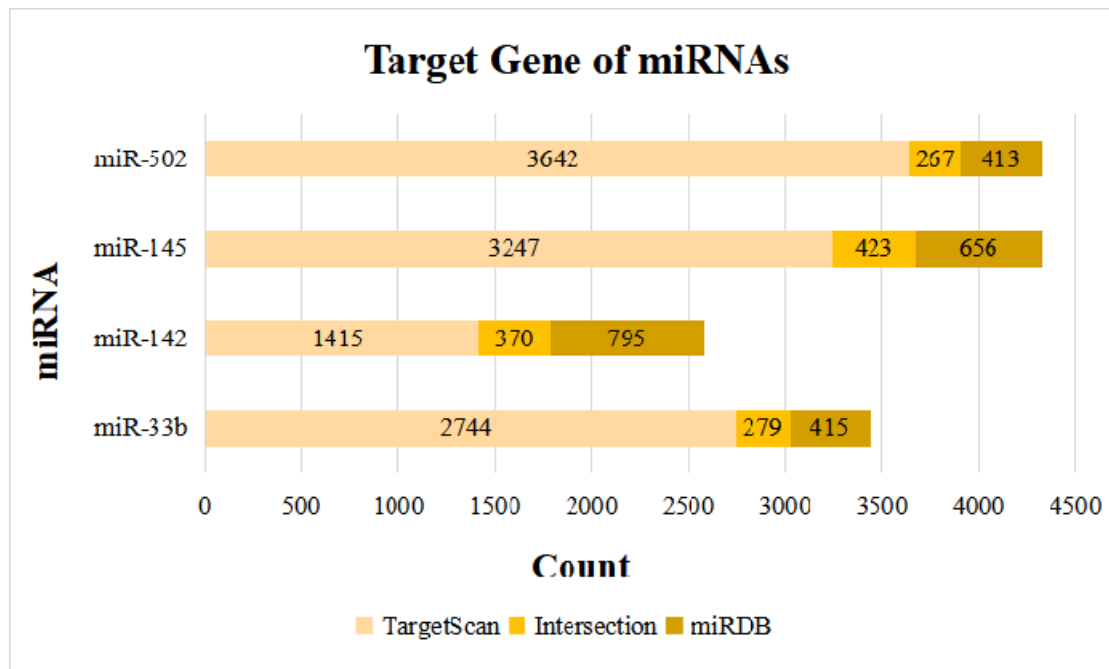

**Figure S1.** Target gene prediction of four miRNAs

Supplement: Supplementary file 1 [file Image_1.pdf]
