# Supplementary material for: Identification of Prognostic miRNA Signature and Lymph Node Metastasis-Related Key Genes in Cervical Cancer
Source: Front Pharmacol. 2020 May 8;11:544. doi: 10.3389/fphar.2020.00544 (PMC7226536; doi:10.3389/fphar.2020.00544)
Supplement: Supplementary file 2 [file DataSheet_1.doc]

**Topology parameters analysis and module analysis of protein-protein interaction network**

**Method:** CytoHubba (Chin et al., 2014), a Cytoscape plugin, was utilized to carry out topology parameters analysis of the protein-protein interaction network (PPI). The top 20 hub genes were selected for module analysis based on Maximal Clique Centrality (MCC). The Molecular Complex Detection (MCODE) plug-in in Cytoscape was used to calculate MCODE score and select the significant modules of hub genes with the screening criteria of Degree Cut-off = 2, Haircut on, Node Score Cut-off = 0.2, k-core = 2 and Max. Depth = 100.

**Result:** The top 20 hub genes in the MCC method were chosen by CytoHubba plugin and sequentially ordered as follows: CXCL12, IGF1, PTPRC, PLXNA4, CDH5, GDNF, SLIT3, SEMA6A, CNTN2, ACTB, WDHD1, RAD51B, ATP1A2, KLHL3, SEMA7A, TPM3, NBEA, TNRC18, REV3L, and TLR6 (Figure S2A). Two important modules were screened by MCODE, in which seven key genes: CXCL12, IGF1, PTPRC, CDH5, RAD51B, REV3L and WDHD1 were obtained. (Figure S2B).

| 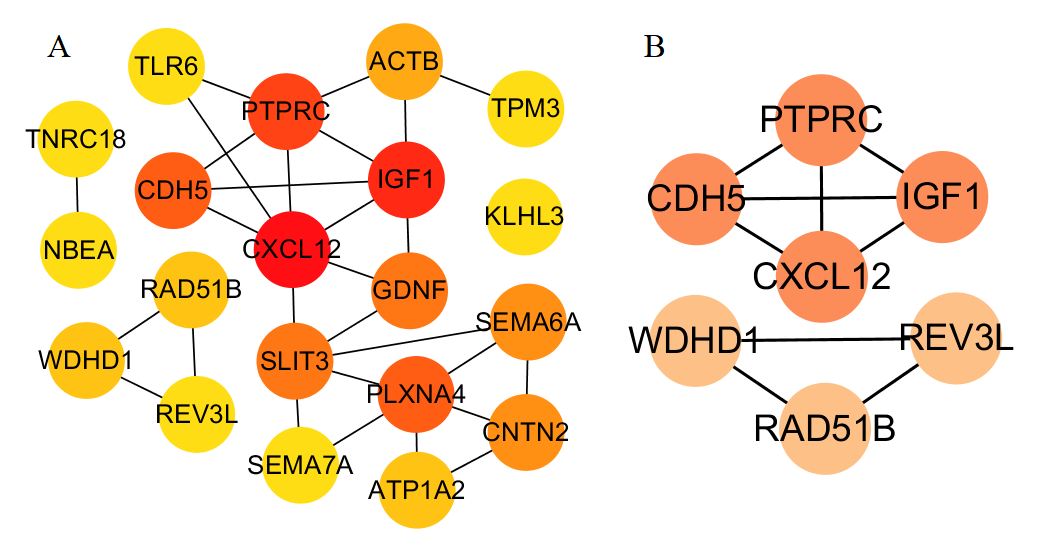 |
| --- |

**Figure S2.** The results of topology parameters analysis and module analysis in protein-protein interaction network. Note: (A) The color of the circle represents the MMC level. Red to yellow represents the MCC level of genes from high to low. (B) The shade of the circle represents MCODE score. Dark to light represents MCODE score of genes from high to low.

**Reference**

Chin, C.-H., Chen, S.-H., Wu, H.-H., Ho, C.-W., Ko, M.-T., and Lin, C.-Y. (2014). cytoHubba: identifying hub objects and sub-networks from complex interactome*. BMC systems biolo*gy 8 Suppl 4, S11. doi: 10.1186/1752-0509-8-S4-S11.
