# Supplementary material for: Identification of Prognostic miRNA Signature and Lymph Node Metastasis-Related Key Genes in Cervical Cancer
Source: Front Pharmacol. 2020 May 8;11:544. doi: 10.3389/fphar.2020.00544 (PMC7226536; doi:10.3389/fphar.2020.00544)
Supplement: Supplementary file 3 [file Table_1.pdf]

Table S1. Analysis of differential expression miRNAs from TCGA.

	logFC	logCPM	P	FDR
**down-regulated miRNAs**				
miR-133a-2	-4.61364793	2.816574556	2.87E-24	1.12E-21
miR-133a-1	-4.492068447	2.895555056	5.89E-22	1.84E-19
miR-145	-4.334337494	10.70205181	3.10E-33	2.43E-30
miR-1-2	-4.179884603	3.48548292	1.39E-17	3.64E-15
miR-204	-4.142216195	3.264312479	2.93E-08	2.29E-06
miR-1-1	-4.140116667	3.402787342	4.35E-17	9.73E-15
miR-129-1	-3.920484734	1.723719221	1.51E-10	2.07E-08
miR-129-2	-3.912979517	1.860153121	6.31E-11	9.87E-09
miR-133b	-3.874049201	1.003366624	1.60E-10	2.07E-08
miR-10b	-3.821418669	15.18902182	8.24E-34	1.29E-30
miR-873	-3.767688341	-0.118686532	2.03E-05	0.000621913
miR-383	-3.765105341	-0.705505712	0.000438357	0.009026692
miR-1298	-3.48221761	0.407313978	0.000600708	0.011191755
miR-6507	-3.244123751	-0.233965602	1.30E-05	0.000451632
miR-3622a	-3.239994247	-0.591891699	6.10E-06	0.000251109
miR-100	-3.222117051	11.57632773	1.72E-10	2.07E-08
miR-143	-3.180279723	16.30591455	2.28E-14	4.47E-12
miR-504	-3.15358165	-0.344205883	3.95E-07	2.38E-05
miR-548ba	-3.099308634	-1.057734008	3.03E-05	0.000833186
miR-876	-2.953530945	-0.694143473	0.001573612	0.02724995
miR-99a	-2.856912466	10.09533009	8.82E-07	5.11E-05
miR-140	-2.819064135	9.762050657	2.30E-26	1.20E-23
miR-1225	-2.798982152	-1.107251412	0.000211774	0.004873923
miR-139	-2.777311112	4.942253745	5.25E-14	9.13E-12
miR-125b-1	-2.655850196	8.720586852	1.11E-08	1.03E-06
miR-3199-1	-2.593719253	-0.571136681	1.17E-06	6.53E-05
miR-125b-2	-2.556238443	8.879029209	1.20E-07	8.19E-06
miR-3926-1	-2.545426097	-0.284832179	1.66E-05	0.000563434
miR-548aw	-2.439141395	-0.671727515	4.19E-06	0.000183746
miR-381	-2.357541768	5.466828175	3.01E-07	1.88E-05
miR-1468	-2.334721286	2.110849838	4.41E-08	3.28E-06
miR-6892	-2.323192347	1.078323974	2.81E-07	1.83E-05
miR-195	-2.167983731	5.10997453	1.80E-06	9.39E-05
miR-3926-2	-2.161231023	0.012858433	0.000203385	0.004750716
miR-1245a	-2.141841771	1.013286642	3.03E-05	0.000833186
hsa-let-7c	-2.1374442	11.78805406	0.000485309	0.009614041
miR-887	-2.112220648	1.600948584	2.79E-06	0.000132504
miR-125a	-2.086259124	9.216011494	1.51E-09	1.57E-07
miR-151b	-2.05694391	0.724764946	5.53E-06	0.000233986

miR-218-1	-2.044845625	4.38155652	0.000152179	0.003780328
miR-218-2	-2.040837871	4.32965091	0.000183998	0.004416019
miR-497	-1.967640433	4.117301947	9.37E-06	0.00034363
miR-362	-1.916002999	4.014265382	3.64E-06	0.000167487
miR-101-2	-1.888239979	12.39105578	2.05E-06	0.000103389
miR-379	-1.880663365	9.256038442	0.000272943	0.006016273
miR-101-1	-1.871523895	12.3776525	2.65E-06	0.000129737
miR-320a	-1.866158614	9.441986648	1.94E-08	1.60E-06
miR-28	-1.778919007	12.09536598	8.08E-09	7.90E-07
miR-299	-1.753098775	2.007373437	0.00071121	0.012942364
miR-495	-1.701195511	2.71794076	0.002200712	0.034789031
miR-127	-1.626272333	9.204805911	0.001929164	0.031125173
miR-99b	-1.454275567	14.23232252	0.000256374	0.005731785
miR-199a-1	-1.441997839	10.19481949	0.002042295	0.032614201
miR-29a	-1.413483079	12.67977396	0.0004699	0.00942812
miR-542	-1.353488336	8.282286202	0.000842767	0.015160122
miR-181d	-1.343643676	4.217224029	0.003027035	0.045117243
miR-23b	-1.338196388	10.49425843	0.000447288	0.009090975
miR-5000	-1.318957219	1.59092211	0.001630024	0.027429981
miR-26a-2	-1.258957418	10.13497051	0.00015758	0.003853334
miR-26a-1	-1.250673132	10.1264852	0.000186235	0.004416019
miR-126	-1.151535198	10.6137652	0.00038699	0.008075201
miR-328	-1.138827981	4.212564805	0.003220336	0.047545522
miR-502	-1.020972078	3.62582368	0.003399409	0.048364322
miR-30b	-1.005599643	8.470404935	0.002469588	0.038266395
**up-regulated miRNAs**				
miR-331	1.381724653	4.654088941	0.002834678	0.043070592
miR-93	1.66783919	12.85944783	0.001828361	0.029806098
miR-185	1.707980215	6.759655278	0.003511178	0.049504441
miR-106b	1.879598294	9.866589016	1.80E-05	0.000584127
miR-21	1.887731252	18.57694595	0.000293175	0.006285196
miR-425	2.111827037	8.133486597	0.002815749	0.043070592
miR-16-1	2.11825157	8.651450457	8.68E-05	0.002226962
miR-7706	2.146327909	1.456812441	0.003372009	0.048364322
miR-1307	2.167021711	10.48973699	2.92E-05	0.000831236
miR-32	2.21163609	5.536871762	2.58E-05	0.000762126
miR-16-2	2.221212836	8.713074254	4.44E-05	0.001158446
miR-1976	2.498691019	3.564961516	8.60E-06	0.000328108
miR-3613	2.597803802	4.112764306	1.98E-05	0.000620247
miR-15b	2.607368782	8.854129587	8.15E-06	0.000318873
miR-942	2.644167723	3.805376847	2.24E-05	0.000672971
miR-1277	2.701811383	0.832627077	0.001802827	0.029699195
miR-4746	2.964198693	2.996693574	0.000232009	0.005262235

miR-200b	3.009056671	10.33317564	0.000596993	0.011191755
miR-18a	3.049877851	4.789413764	3.59E-05	0.000967668
miR-877	3.079010902	1.689847334	0.003375895	0.048364322
miR-130b	3.174166874	5.518453424	4.23E-06	0.000183746
miR-940	3.3140794	2.114971862	0.000576629	0.011005179
miR-2277	3.467436689	1.035689814	0.00095293	0.016803488
miR-142	3.58878167	11.33132207	4.02E-05	0.001065237
miR-224	3.591443384	7.632134479	0.001584502	0.02724995
miR-301b	3.619752404	1.162731545	0.003306324	0.048358848
miR-106a	3.652642752	5.555032096	0.00162813	0.027429981
miR-3614	3.708769013	3.020994678	0.000955598	0.016803488
miR-33b	3.76338394	2.924870762	0.000542572	0.01049199
miR-3934	3.920011294	2.452251431	6.56E-06	0.000263305
miR-200a	3.958614276	10.6768952	9.44E-06	0.00034363
miR-135b	3.982711508	6.052031437	0.000665431	0.012251756
miR-363	4.089291427	5.947188393	0.002278582	0.035659808
miR-429	4.117058484	7.666462631	1.83E-05	0.000584127
miR-200c	4.385256579	13.46194277	1.80E-08	1.56E-06
miR-182	4.529229595	14.30949356	1.15E-05	0.000408681
miR-20b	4.583122628	6.955048778	0.002969903	0.044691324
miR-203b	4.898296657	7.280647958	0.0003255	0.006883882
miR-210	4.934176636	10.36037796	2.80E-05	0.000812183
miR-141	5.038865651	11.01981366	1.06E-09	1.18E-07
miR-183	5.546671512	13.03811056	1.52E-06	8.18E-05
miR-31	5.645612033	7.079591706	0.000124845	0.003151323
miR-96	5.833092155	4.895357213	8.93E-08	6.35E-06
miR-203a	5.951295098	15.81324122	0.000277906	0.006040589
miR-205	7.881335681	13.77042635	1.72E-05	0.0005719
miR-944	8.165937755	6.669846813	0.000543036	0.01049199
