# Supplementary material for: Identification of Prognostic miRNA Signature and Lymph Node Metastasis-Related Key Genes in Cervical Cancer
Source: Front Pharmacol. 2020 May 8;11:544. doi: 10.3389/fphar.2020.00544 (PMC7226536; doi:10.3389/fphar.2020.00544)
Supplement: Supplementary file 4 [file Table_2.pdf]

Table S2. Analysis of differential expression genes from GEO.

Gene	FDR	P	t	B	logFC
**down-regulated genes**					
PIP	3.12E-06	1.04E-07	-7.28	7.8054906	-1.00001153
MEGF10	1.80E-07	1.92E-09	-9.01	11.7434385	-1.00011802
ADCY7	7.76E-08	4.43E-10	-9.69	13.1813425	-1.00015818
REG1A	8.41E-05	8.05E-06	-5.56	3.5063975	-1.00043835
CCR4	2.71E-06	8.56E-08	-7.36	7.9963122	-1.00193296
LINC01105	3.64E-06	1.29E-07	-7.19	7.5931445	-1.00197688
GLO1	3.57E-06	1.25E-07	-7.2	7.6193038	-1.0020093
HIST1H2AG	7.35E-04	1.37E-04	-4.47	0.7233753	-1.0021019
BCAS3	1.76E-04	2.08E-05	-5.19	2.5692328	-1.00211059
ZNF471	3.26E-04	4.71E-05	-4.88	1.7665309	-1.00226604
FZR1	1.43E-05	8.20E-07	-6.45	5.7619847	-1.00234243
EGFL7	1.80E-07	1.90E-09	-9.01	11.750439	-1.00250693
PRAME	9.08E-08	5.92E-10	-9.55	12.8964452	-1.0028502
LINC01558	5.04E-08	2.20E-10	-10	13.8638771	-1.0028984
GUCY1A2	5.82E-04	1.01E-04	-4.59	1.017735	-1.00299418
ZCCHC4	1.64E-03	3.89E-04	-4.08	-0.2956998	-1.00308592
CLDN2	6.76E-05	6.10E-06	-5.66	3.779757	-1.0032232
ACSM5	9.86E-07	2.17E-08	-7.94	9.3545912	-1.0034023
CD300LG	1.69E-03	4.06E-04	-4.06	-0.3369623	-1.0035991
CDON	1.17E-06	2.74E-08	-7.84	9.1213618	-1.00385255
C7orf69	1.15E-05	6.19E-07	-6.56	6.0401007	-1.00417746
DBF4B	7.17E-08	3.72E-10	-9.77	13.3518668	-1.00433692
MMAB	9.83E-07	2.15E-08	-7.94	9.3597154	-1.00505427
KIF13A	3.46E-03	9.67E-04	-3.72	-1.1762428	-1.00528504
HNF4G	2.56E-07	3.30E-09	-8.76	11.2095923	-1.00535563
ARHGAP6	9.02E-04	1.79E-04	-4.37	0.4617841	-1.00560342
SCAMP1	7.62E-07	1.50E-08	-8.1	9.7184345	-1.00577071
CA8	9.46E-07	2.03E-08	-7.97	9.4206566	-1.0060513
MIR100HG	2.46E-05	1.62E-06	-6.18	5.087011	-1.00607162
CYP4F11	1.92E-05	1.18E-06	-6.3	5.3997715	-1.00610789
FAM86C2P	2.58E-04	3.45E-05	-5	2.0726245	-1.00656659
ITPK1	1.09E-06	2.48E-08	-7.88	9.2211112	-1.00695417
PML	7.65E-05	7.14E-06	-5.6	3.6243757	-1.00705629
AP1S2	2.80E-05	1.92E-06	-6.11	4.9204151	-1.00709019
UNC45B	1.28E-05	7.06E-07	-6.51	5.9105213	-1.00718704
DOK6	6.56E-04	1.18E-04	-4.53	0.8687763	-1.00724357
HTRA1	1.78E-03	4.34E-04	-4.03	-0.4005441	-1.00748159
EML6	6.86E-05	6.21E-06	-5.65	3.7617068	-1.0076013
RUNDC3B	1.02E-07	7.35E-10	-9.45	12.6838517	-1.00764415
KCNG2	2.44E-04	3.21E-05	-5.03	2.143721	-1.00807455
SH3BGRL2	4.64E-03	1.38E-03	-3.58	-1.5176544	-1.00812613
PAQR7	8.86E-06	4.34E-07	-6.7	6.3923316	-1.00836736
LRRC4B	8.64E-03	2.91E-03	-3.29	-2.2309001	-1.00851992
LOC101060157	6.69E-04	1.21E-04	-4.52	0.8442883	-1.00888359
PLEKHA7	4.52E-05	3.59E-06	-5.87	4.3038216	-1.00902683
FLJ32742	7.41E-07	1.44E-08	-8.12	9.758116	-1.00906979
TRHDE	1.04E-03	2.16E-04	-4.3	0.2761285	-1.00977002
ABCA6	1.95E-03	4.87E-04	-3.99	-0.5129532	-1.00978893
TMEM245	2.18E-04	2.78E-05	-5.08	2.2854183	-1.00995216

TGM2	8.42E-04	1.63E-04	-4.41	0.5511947	-1.01012771
TDH	8.23E-04	1.59E-04	-4.42	0.5788053	-1.0102131
AGER	6.20E-04	1.10E-04	-4.56	0.9395272	-1.01031432
NOP14	1.96E-04	2.39E-05	-5.14	2.432844	-1.0104853
LOC105374366	1.44E-03	3.27E-04	-4.14	-0.1263053	-1.01065586
ZNRD1ASP	3.21E-04	4.62E-05	-4.89	1.7852195	-1.01100221
SPATA13	3.92E-04	6.04E-05	-4.79	1.5231975	-1.01234774
CCR10	3.17E-06	1.06E-07	-7.27	7.7819524	-1.01240145
TSPAN14	7.09E-07	1.35E-08	-8.14	9.8232355	-1.01244445
PIGS	4.95E-07	8.18E-09	-8.36	10.3144407	-1.01266134
TERB1	1.58E-03	3.70E-04	-4.09	-0.2474635	-1.01267294
CCDC65	4.29E-04	6.75E-05	-4.74	1.4138395	-1.0130745
GLP1R	1.47E-03	3.36E-04	-4.13	-0.1535685	-1.01364203
ZNF765	6.42E-06	2.83E-07	-6.87	6.8142777	-1.01462263
TMEM92	1.03E-03	2.14E-04	-4.3	0.2883074	-1.01462993
TTTY6	3.78E-06	1.36E-07	-7.17	7.5376771	-1.01481669
SNRPN///IPW	9.38E-05	9.28E-06	-5.5	3.3658484	-1.01488276
MBD5	3.74E-06	1.34E-07	-7.18	7.5524648	-1.01509766
BCL7A	1.36E-06	3.33E-08	-7.76	8.9287045	-1.01576922
USP22	1.02E-03	2.09E-04	-4.31	0.3093367	-1.01591699
SLC6A4	3.65E-06	1.30E-07	-7.19	7.5864995	-1.01621373
FGF2	5.30E-06	2.17E-07	-6.98	7.0749979	-1.01655823
SH3BP5	1.98E-04	2.44E-05	-5.13	2.4135943	-1.01657919
ATF2	3.76E-05	2.81E-06	-5.96	4.543893	-1.0167309
DAPK1	2.89E-05	2.00E-06	-6.09	4.8788126	-1.0168308
C1orf105	4.08E-05	3.14E-06	-5.92	4.4367506	-1.01691593
DPYSL5	6.21E-07	1.13E-08	-8.22	9.9997959	-1.0172878
KIT	1.80E-06	4.99E-08	-7.59	8.5306304	-1.01732931
PRKAR2B	3.69E-05	2.75E-06	-5.97	4.5667317	-1.01741232
GREM2	3.05E-04	4.30E-05	-4.91	1.8565841	-1.01748802
CDK13	3.27E-06	1.12E-07	-7.25	7.7341376	-1.01771438
CCND3	2.43E-06	7.39E-08	-7.42	8.1421157	-1.01785347
MAGEA9	2.15E-07	2.48E-09	-8.89	11.4917296	-1.01825427
GDF11	4.97E-04	8.18E-05	-4.67	1.2260172	-1.01860088
PRAMEF8	9.15E-07	1.93E-08	-7.99	9.4681618	-1.01876635
LINC01556	5.88E-06	2.52E-07	-6.92	6.9304204	-1.01881621
ZBED5	5.76E-06	2.45E-07	-6.93	6.9583956	-1.01905144
SEZ6L	5.37E-04	9.06E-05	-4.63	1.1259775	-1.01909837
LOC284242	4.03E-08	1.44E-10	-10.2	14.2786553	-1.02053365
TMEM17	3.38E-04	4.97E-05	-4.86	1.7136023	-1.02091032
SNTG1	7.38E-07	1.43E-08	-8.12	9.7637745	-1.02104072
DAG1	4.21E-05	3.26E-06	-5.9	4.396842	-1.02127576
C16orf45	3.14E-05	2.23E-06	-6.05	4.7748911	-1.0213108
FLRT3	7.23E-03	2.35E-03	-3.37	-2.0276296	-1.0215305
FAIM2	1.03E-06	2.28E-08	-7.92	9.3026022	-1.02185674
LINC01314	1.25E-06	3.00E-08	-7.8	9.0328047	-1.02209317
NKAIN2	2.45E-04	3.23E-05	-5.02	2.1370226	-1.02216689
SNHG25///SNORA50C	8.41E-05	8.04E-06	-5.56	3.5070453	-1.02228318
KRTAP17-1	8.66E-07	1.79E-08	-8.02	9.5399575	-1.02244655
TMEM14EP	6.93E-09	8.67E-12	-11.7	17.0080122	-1.02259477
HDDC2	3.48E-07	5.06E-09	-8.57	10.7879611	-1.02320346
UNC93A	1.53E-03	3.55E-04	-4.11	-0.2067979	-1.02345536
DLK1	3.17E-04	4.54E-05	-4.89	1.8027319	-1.02414025
TMEM27	5.39E-04	9.10E-05	-4.63	1.1215284	-1.02417615

CCDC57	9.81E-06	4.97E-07	-6.65	6.2577402	-1.02432107
RGAG1	2.04E-05	1.28E-06	-6.27	5.3251052	-1.02458992
IFNA14	7.74E-07	1.53E-08	-8.09	9.6984194	-1.02564766
PCDHGB4	4.23E-06	1.59E-07	-7.11	7.3838094	-1.02567808
PRKCDBP	9.70E-06	4.90E-07	-6.65	6.2707432	-1.02590467
EPT1	1.39E-04	1.54E-05	-5.31	2.8697352	-1.0260564
PAGE1	2.12E-05	1.34E-06	-6.25	5.2765185	-1.02636999
OTP	1.53E-04	1.75E-05	-5.26	2.7405699	-1.02661418
LPAR1	1.03E-05	5.34E-07	-6.62	6.1856559	-1.02682232
CTGF	1.62E-03	3.84E-04	-4.08	-0.2834927	-1.02746033
FOXO4	2.61E-05	1.75E-06	-6.15	5.0113052	-1.02775291
RAB43	3.01E-05	2.11E-06	-6.07	4.8264326	-1.02782108
PAX3	2.47E-05	1.63E-06	-6.17	5.080765	-1.02809386
MUSTN1	2.34E-08	5.48E-11	-10.7	15.2207368	-1.02841418
CALD1	3.71E-05	2.77E-06	-5.97	4.5584177	-1.02855711
PCK1	6.91E-06	3.13E-07	-6.83	6.7157437	-1.02891053
REV3L	6.79E-05	6.13E-06	-5.66	3.7753901	-1.02891063
CNIH3	1.14E-07	8.88E-10	-9.36	12.4993985	-1.02931482
C14orf37	3.29E-06	1.12E-07	-7.25	7.7281527	-1.02940063
WFIKKN1	9.02E-08	5.85E-10	-9.56	12.9084852	-1.03052589
NKX6-2	1.10E-06	2.50E-08	-7.88	9.2143998	-1.03063232
CNTN2	2.90E-06	9.42E-08	-7.32	7.9022035	-1.0318512
TCEAL5	3.32E-04	4.84E-05	-4.87	1.7415324	-1.0318554
PLA2R1	1.21E-06	2.86E-08	-7.82	9.0802917	-1.03186062
PRICKLE4	2.20E-04	2.80E-05	-5.08	2.2767041	-1.03187437
KCTD17	5.84E-07	1.02E-08	-8.26	10.094527	-1.03206854
MRO	1.78E-04	2.11E-05	-5.19	2.5545471	-1.03214104
TMPRSS11F	1.88E-06	5.27E-08	-7.56	8.4762606	-1.03244571
PXDN	5.20E-05	4.33E-06	-5.79	4.1177321	-1.03261934
FAM175A	5.23E-06	2.13E-07	-6.99	7.0946855	-1.03282407
SPG20	3.18E-07	4.48E-09	-8.63	10.9072771	-1.03322032
CNRIP1	1.09E-05	5.76E-07	-6.59	6.1110452	-1.0336724
KIF5C	7.04E-03	2.28E-03	-3.39	-1.9969624	-1.03375177
DLC1	2.40E-05	1.57E-06	-6.19	5.1186715	-1.03376829
ZNFX1	4.12E-04	6.43E-05	-4.76	1.4622062	-1.03386273
OR2H2	2.33E-07	2.84E-09	-8.83	11.356374	-1.03450724
C4orf22	2.43E-05	1.60E-06	-6.18	5.1004564	-1.03457496
SH3PXD2A	1.27E-06	3.05E-08	-7.79	9.016371	-1.03493506
GATC	2.21E-06	6.46E-08	-7.48	8.2746454	-1.03501308
MTX3	1.84E-03	4.53E-04	-4.02	-0.442235	-1.03513894
SPON2	7.31E-03	2.38E-03	-3.37	-2.0404094	-1.03527206
FRRS1	2.38E-06	7.19E-08	-7.43	8.1687984	-1.03542141
LRRC73	9.25E-06	4.60E-07	-6.68	6.3332339	-1.03549442
WFDC1	3.06E-06	1.01E-07	-7.29	7.8297455	-1.03575774
CASZ1	9.37E-06	4.70E-07	-6.67	6.3136467	-1.03615007
PXMP2	4.77E-06	1.86E-07	-7.04	7.2291569	-1.03635928
PXK	3.17E-05	2.25E-06	-6.05	4.7625529	-1.03766902
PAPLN	1.16E-07	9.23E-10	-9.35	12.461285	-1.03795075
TMPRSS6	1.15E-05	6.19E-07	-6.56	6.0400015	-1.03814801
SRPX	1.55E-04	1.78E-05	-5.25	2.7233755	-1.03821598
RBPJL	1.51E-07	1.46E-09	-9.13	12.0098527	-1.03875032
ZMAT4	2.42E-07	3.02E-09	-8.8	11.2967281	-1.03876326
C4BPA	2.36E-04	3.08E-05	-5.04	2.1860648	-1.03927686
PDE6G	9.05E-06	4.48E-07	-6.69	6.360063	-1.04003403

ANKMY1	3.06E-04	4.32E-05	-4.91	1.8523627	-1.04014315
NT5C1B	3.63E-06	1.28E-07	-7.19	7.5972899	-1.04036835
MEOX2	3.21E-08	9.51E-11	-10.4	14.683772	-1.04093164
HYOU1	2.84E-04	3.91E-05	-4.95	1.9506157	-1.04094123
CFHR4	8.41E-05	8.04E-06	-5.56	3.5067233	-1.04161874
ZNF175	2.14E-03	5.44E-04	-3.95	-0.6202091	-1.04176999
ATOH8	6.00E-06	2.59E-07	-6.91	6.9020787	-1.04188655
STXBP5	4.73E-06	1.84E-07	-7.05	7.2391001	-1.0419726
USP51	6.23E-06	2.72E-07	-6.89	6.8547785	-1.04229755
CMTR2	3.77E-05	2.82E-06	-5.96	4.5402989	-1.04243924
KCNN3	6.42E-07	1.18E-08	-8.2	9.9531207	-1.04346914
ACOX2	2.50E-06	7.67E-08	-7.41	8.1051433	-1.04444159
MYL1	7.75E-04	1.46E-04	-4.45	0.6564007	-1.04512557
LOC441178	2.37E-07	2.92E-09	-8.82	11.3306797	-1.04536859
BAAT	8.41E-05	8.05E-06	-5.56	3.5060505	-1.04579214
GCRG224	1.31E-04	1.42E-05	-5.34	2.9449622	-1.04594516
LINC01111	3.37E-04	4.96E-05	-4.86	1.7173969	-1.04610275
SPI1	4.62E-05	3.69E-06	-5.86	4.2745815	-1.04610666
POLR3A	2.22E-05	1.42E-06	-6.23	5.2161495	-1.0461758
STAB1	3.65E-09	1.54E-12	-12.6	18.671314	-1.04639877
HDLBP	2.16E-05	1.38E-06	-6.24	5.2484146	-1.04647638
SEZ6	2.15E-07	2.49E-09	-8.89	11.486238	-1.04650739
LINC00327	4.05E-04	6.27E-05	-4.77	1.4860996	-1.04679333
CFAP43	1.19E-06	2.80E-08	-7.83	9.1009397	-1.04694341
FRAS1	1.47E-02	5.52E-03	-3.03	-2.8371873	-1.0469473
DNAH5	1.15E-05	6.14E-07	-6.56	6.0480502	-1.04734523
CA5B	1.75E-05	1.06E-06	-6.34	5.5098855	-1.04749536
EIF3L	2.96E-05	2.07E-06	-6.08	4.847636	-1.04765647
SEPT8	1.54E-05	8.96E-07	-6.41	5.6743736	-1.0477985
ERBB2	1.16E-04	1.22E-05	-5.4	3.0997801	-1.04780518
ZFP14	5.54E-05	4.69E-06	-5.76	4.0381074	-1.04809861
KNG1	5.66E-04	9.72E-05	-4.6	1.0573966	-1.04815222
IL10RA	8.71E-05	8.43E-06	-5.54	3.4600359	-1.04853531
DUSP8	4.64E-04	7.47E-05	-4.7	1.3147177	-1.0485869
LTBP2	3.68E-06	1.31E-07	-7.19	7.5764971	-1.04888093
KMT5A	3.37E-04	4.96E-05	-4.86	1.7172273	-1.04888294
CFC1	6.04E-07	1.07E-08	-8.24	10.0469509	-1.0489511
C16orf89	2.26E-05	1.46E-06	-6.22	5.1928515	-1.04919939
ZNF76	8.58E-05	8.28E-06	-5.54	3.4777772	-1.04946054
KRTAP9-8	6.92E-06	3.14E-07	-6.83	6.712697	-1.04957469
SYTL4	2.15E-04	2.73E-05	-5.09	2.3048199	-1.04983556
ZSCAN21	1.28E-03	2.82E-04	-4.2	0.0164251	-1.05025793
TCAF2	8.02E-07	1.62E-08	-8.06	9.6407619	-1.05068978
AGTR1	1.22E-05	6.71E-07	-6.53	5.9613693	-1.05101637
MAP2	7.65E-05	7.14E-06	-5.6	3.6247547	-1.05108961
SLC25A42	4.21E-05	3.27E-06	-5.9	4.3966279	-1.05164328
OLFML1	3.34E-08	1.04E-10	-10.4	14.5965466	-1.05186982
ESM1	1.45E-07	1.35E-09	-9.17	12.0862174	-1.05206664
FBXO22	1.74E-05	1.05E-06	-6.35	5.5207088	-1.05220165
PRSS37	4.49E-06	1.72E-07	-7.08	7.3091084	-1.05227273
PTGS1	3.82E-03	1.09E-03	-3.68	-1.2907496	-1.05227816
CHRNE	1.01E-07	7.12E-10	-9.47	12.7153563	-1.05250426
GRIA2	6.00E-05	5.22E-06	-5.72	3.9340622	-1.05254068
PCDHB13	5.93E-06	2.55E-07	-6.91	6.9175459	-1.05340787

KCNN1	2.00E-05	1.25E-06	-6.28	5.3480582	-1.05358792
FENDRR	2.73E-04	3.72E-05	-4.97	2.0000169	-1.05364187
GSTK1	2.14E-07	2.46E-09	-8.9	11.4971357	-1.05412555
CDH18	1.49E-07	1.41E-09	-9.15	12.045899	-1.05421839
MLXIPL	3.25E-05	2.33E-06	-6.04	4.7320965	-1.054644
FAM81A	1.00E-06	2.21E-08	-7.93	9.3343552	-1.05513438
CAPN8	2.06E-03	5.19E-04	-3.96	-0.5747239	-1.05513656
GADD45G	1.14E-06	2.65E-08	-7.85	9.1551574	-1.05569195
GUCA1C	2.08E-05	1.31E-06	-6.26	5.3001002	-1.05578632
DKK4	5.37E-04	9.05E-05	-4.63	1.1270734	-1.05580111
AFF3	1.28E-05	7.08E-07	-6.5	5.9079124	-1.0564339
PDE11A	6.11E-04	1.08E-04	-4.57	0.956582	-1.05655264
SOX10	2.37E-08	5.73E-11	-10.7	15.1780503	-1.05659504
C2orf54	1.58E-02	5.99E-03	-2.99	-2.9137978	-1.05739748
TMEM132B	2.87E-05	1.99E-06	-6.1	4.8868433	-1.05767756
PLEKHB1	6.47E-06	2.87E-07	-6.87	6.8014874	-1.05776254
UGT2A3	3.43E-05	2.50E-06	-6.01	4.6605752	-1.05780403
CPB1	1.67E-04	1.94E-05	-5.22	2.6380559	-1.05782529
SH2D5	1.63E-07	1.65E-09	-9.08	11.8886372	-1.05793558
ATP1A1-AS1	8.97E-05	8.76E-06	-5.52	3.423004	-1.05813323
RAPGEF4	3.74E-08	1.25E-10	-10.3	14.4171547	-1.05813486
RORC	2.60E-08	6.50E-11	-10.6	15.0544121	-1.05822357
LCA5L	2.34E-04	3.04E-05	-5.05	2.1958023	-1.05857071
MATN4	1.04E-07	7.51E-10	-9.44	12.6630822	-1.05887485
WBSCR28	2.32E-07	2.80E-09	-8.84	11.3694614	-1.05903927
FAM150A	4.60E-06	1.77E-07	-7.06	7.2788946	-1.0591007
RAPGEF3	8.45E-06	4.07E-07	-6.73	6.454204	-1.05938899
LVCAT5	3.88E-06	1.41E-07	-7.16	7.5019833	-1.0596334
MBLAC2	2.54E-04	3.37E-05	-5.01	2.0965357	-1.05963479
SLC4A4	1.60E-02	6.09E-03	-2.99	-2.9289961	-1.05997545
LOC284244	1.08E-03	2.27E-04	-4.28	0.2306775	-1.06001407
SLC6A2	6.81E-04	1.24E-04	-4.51	0.8209507	-1.06013444
ANKH	2.77E-04	3.80E-05	-4.96	1.9793369	-1.06021417
RBBP5	4.24E-07	6.65E-09	-8.45	10.519684	-1.06083023
PRICKLE1	2.68E-06	8.41E-08	-7.37	8.0148174	-1.06091436
FAM19A5	6.67E-06	2.99E-07	-6.85	6.7614676	-1.06100144
GPR173	2.84E-07	3.84E-09	-8.7	11.0599102	-1.061007
SMIM2	1.80E-04	2.14E-05	-5.18	2.5428852	-1.06107265
TM4SF5	3.87E-08	1.30E-10	-10.3	14.3765673	-1.06178625
SLC19A3	1.85E-04	2.22E-05	-5.17	2.5075017	-1.06184422
PLPPR2	1.20E-08	1.97E-11	-11.2	16.2132579	-1.06203447
ABRA	9.54E-07	2.05E-08	-7.96	9.4087841	-1.06211742
PHACTR3	1.63E-07	1.63E-09	-9.08	11.9019828	-1.06262166
ESR2	5.53E-06	2.31E-07	-6.95	7.0160628	-1.06267135
XYLT1	1.02E-06	2.27E-08	-7.92	9.3071212	-1.0629675
WDR19	4.36E-05	3.43E-06	-5.89	4.3492214	-1.06317717
LOC102723694	1.95E-04	2.39E-05	-5.14	2.4354932	-1.06355319
KNDC1	1.01E-07	7.18E-10	-9.46	12.7074963	-1.06395668
TPCN1	1.49E-07	1.41E-09	-9.15	12.0436788	-1.06402436
ROPN1	6.20E-07	1.12E-08	-8.22	10.0054044	-1.06428295
TAS2R13	3.23E-07	4.58E-09	-8.62	10.8864791	-1.06534186
GLIS3	1.06E-04	1.08E-05	-5.44	3.2152206	-1.06575649
PLEKHS1	2.86E-02	1.22E-02	-2.7	-3.5757498	-1.06584366
CASP2	9.38E-04	1.88E-04	-4.35	0.4129852	-1.06587935

LYG2	1.14E-05	6.09E-07	-6.56	6.0564961	-1.06610382
BTNL9	8.79E-08	5.60E-10	-9.58	12.9512882	-1.06613719
HIST1H2AC	4.05E-05	3.10E-06	-5.92	4.4473957	-1.06637074
CCL25	9.84E-08	6.76E-10	-9.49	12.7664395	-1.06643475
WDR27	6.23E-06	2.72E-07	-6.89	6.8553754	-1.06648459
TMEM204	5.47E-04	9.28E-05	-4.62	1.1027083	-1.06656457
HOXD13	3.45E-05	2.52E-06	-6.01	4.6536707	-1.06657938
LONRF1	5.23E-04	8.77E-05	-4.64	1.157801	-1.06662431
CDH2	4.57E-06	1.75E-07	-7.07	7.2909613	-1.06696456
TAS2R50	7.66E-07	1.51E-08	-8.1	9.7104489	-1.06700095
ITIH6	4.57E-07	7.29E-09	-8.41	10.4284399	-1.06702962
PCDHGC3	7.72E-05	7.22E-06	-5.6	3.6126194	-1.06809143
SLC38A3	1.16E-06	2.69E-08	-7.85	9.1391497	-1.06823018
ORAI2	5.88E-06	2.52E-07	-6.92	6.9279515	-1.06836397
LOC101927598	8.56E-05	8.26E-06	-5.55	3.4802644	-1.06841394
TECTA	3.10E-07	4.34E-09	-8.64	10.9384684	-1.06848489
CELA1	1.77E-06	4.84E-08	-7.6	8.5602148	-1.0688882
DUSP18	4.92E-05	4.03E-06	-5.82	4.1897536	-1.06902799
SP2	1.46E-05	8.42E-07	-6.44	5.7367121	-1.06932399
PAPPA	4.43E-04	7.04E-05	-4.73	1.3735828	-1.06936571
ERAP2	1.60E-03	3.76E-04	-4.09	-0.2622705	-1.069383
PHF23	6.83E-04	1.24E-04	-4.51	0.8152168	-1.06939709
HEXA	1.07E-05	5.57E-07	-6.6	6.1439461	-1.06940595
DBNDD1	3.17E-05	2.26E-06	-6.05	4.7613289	-1.06944289
CCDC121	2.80E-04	3.84E-05	-4.96	1.968038	-1.06947899
IQSEC3	7.55E-08	4.16E-10	-9.72	13.2430556	-1.06976328
CLU	2.18E-03	5.58E-04	-3.94	-0.6442501	-1.07016726
SCN2B	7.15E-04	1.32E-04	-4.49	0.7576916	-1.0702892
LSAMP	4.98E-05	4.10E-06	-5.82	4.1714503	-1.07059894
ADGRF5	2.87E-05	1.99E-06	-6.1	4.8864715	-1.07066353
CLDN10	1.46E-04	1.64E-05	-5.28	2.8030863	-1.07085606
SPEF2	6.53E-05	5.84E-06	-5.68	3.8234476	-1.07169635
PLEKHA5	6.82E-04	1.24E-04	-4.51	0.8179884	-1.07176372
MS4A2	8.34E-08	5.13E-10	-9.62	13.0378004	-1.07195356
DMBX1	3.44E-04	5.10E-05	-4.85	1.6894027	-1.07199087
ZNF214	1.15E-04	1.21E-05	-5.4	3.1045167	-1.0727823
GLDN	1.52E-04	1.73E-05	-5.26	2.7529013	-1.07279174
CPA4	1.33E-04	1.46E-05	-5.33	2.9183497	-1.07285937
MAN2C1	4.19E-06	1.56E-07	-7.11	7.4006895	-1.07303745
TIMP3	2.85E-04	3.93E-05	-4.95	1.9454991	-1.0732039
TUFT1	4.57E-05	3.64E-06	-5.86	4.2891471	-1.07389322
FAM65C	2.48E-07	3.13E-09	-8.79	11.2613379	-1.07389489
TMSB4Y	2.60E-06	8.04E-08	-7.39	8.0583246	-1.07414388
SFTA2	2.82E-06	9.05E-08	-7.34	7.9415329	-1.0743935
ARMCX1	3.97E-05	3.02E-06	-5.93	4.4724051	-1.07479679
ICA1L	1.80E-05	1.10E-06	-6.33	5.475916	-1.07484609
NPAS1	5.00E-06	1.98E-07	-7.02	7.1669187	-1.07486991
CD200R1	1.05E-04	1.07E-05	-5.45	3.2275332	-1.07532756
F11-AS1	8.23E-05	7.80E-06	-5.57	3.5365172	-1.07573839
MAGEH1	1.20E-05	6.56E-07	-6.53	5.9835501	-1.07579741
DNAJC28	2.39E-05	1.57E-06	-6.19	5.122425	-1.07581891
HPCA	1.11E-07	8.46E-10	-9.39	12.5467499	-1.07589213
BBOF1	1.19E-04	1.26E-05	-5.38	3.0627764	-1.07592493
ZNF586	7.56E-06	3.52E-07	-6.78	6.5973315	-1.07673128

GABRQ	6.14E-09	6.40E-12	-11.8	17.3018183	-1.07754042
GRIP2	1.42E-05	8.11E-07	-6.45	5.7731593	-1.07785721
SCARF2	3.06E-06	1.01E-07	-7.29	7.834538	-1.07806423
LOC100996455	2.13E-04	2.69E-05	-5.09	2.3193313	-1.07849571
NXPH3	2.62E-05	1.76E-06	-6.14	5.0055645	-1.07853309
AMOT	1.78E-07	1.86E-09	-9.02	11.7741774	-1.07884184
KANSL1L	2.08E-05	1.31E-06	-6.26	5.2981492	-1.07939244
PMEL	4.57E-06	1.75E-07	-7.07	7.2890209	-1.07970925
CNTLN	3.21E-04	4.62E-05	-4.89	1.7856052	-1.08012263
CARNS1	2.00E-04	2.47E-05	-5.13	2.4030408	-1.08012487
ST8SIA4	6.20E-06	2.70E-07	-6.89	6.8605551	-1.08030424
CD160	5.69E-04	9.79E-05	-4.6	1.0495976	-1.08034957
FGFRL1	1.16E-05	6.27E-07	-6.55	6.0275127	-1.08086018
IFNA21	4.98E-05	4.09E-06	-5.82	4.1744595	-1.08121723
CDK18	1.27E-07	1.09E-09	-9.27	12.2969884	-1.08129843
SLC6A6	3.55E-06	1.24E-07	-7.21	7.6295283	-1.08178585
PRUNE1	1.27E-05	7.02E-07	-6.51	5.9165775	-1.08224249
BMP6	3.66E-06	1.30E-07	-7.19	7.5852861	-1.08244064
BMP1	4.41E-05	3.47E-06	-5.88	4.3356325	-1.08370689
CDH5	2.71E-06	8.55E-08	-7.36	7.9981396	-1.08381393
GDPD1	3.59E-05	2.65E-06	-5.98	4.6023195	-1.08381688
WBSCR17	3.03E-06	1.00E-07	-7.3	7.8433826	-1.08394465
DKK3	2.55E-04	3.40E-05	-5	2.0880075	-1.08415867
ERVW-1	1.04E-07	7.60E-10	-9.44	12.6515962	-1.08510554
MS4A6E	1.42E-06	3.61E-08	-7.72	8.8513072	-1.08525895
BEND6	1.93E-05	1.19E-06	-6.3	5.3938834	-1.08529345
C20orf197	3.66E-05	2.72E-06	-5.98	4.5781598	-1.08550993
POU3F1	5.13E-04	8.53E-05	-4.65	1.1848013	-1.08637075
DNALI1	5.52E-07	9.49E-09	-8.3	10.1688514	-1.08662182
UBQLN3	1.28E-05	7.09E-07	-6.5	5.9060232	-1.08788235
CDKN1C	4.91E-03	1.48E-03	-3.56	-1.581817	-1.08826503
CRYBA2	6.32E-07	1.15E-08	-8.21	9.976881	-1.08829028
FUT9	3.28E-06	1.12E-07	-7.25	7.7304722	-1.08849988
SACS	5.90E-05	5.10E-06	-5.73	3.9572156	-1.08851365
REG3A	1.44E-06	3.68E-08	-7.71	8.8307181	-1.08875308
ZNF414	4.92E-08	2.07E-10	-10.1	13.9237928	-1.0890865
RBMS3	3.51E-03	9.83E-04	-3.72	-1.1920185	-1.09020584
RPL23AP32	2.30E-06	6.91E-08	-7.45	8.2081808	-1.09052653
LRRC17	2.96E-04	4.11E-05	-4.93	1.9001668	-1.09167002
GIPC2	2.80E-05	1.92E-06	-6.11	4.9218944	-1.09187443
CEP170	1.89E-06	5.31E-08	-7.56	8.4696563	-1.09194659
CCDC102B	1.10E-04	1.14E-05	-5.42	3.1655506	-1.09204082
LRRC7	3.04E-05	2.14E-06	-6.07	4.8123731	-1.09206924
ASCL1	1.24E-07	1.06E-09	-9.28	12.32542	-1.09316658
FBXL7	1.18E-07	9.50E-10	-9.33	12.4323065	-1.09334405
METTL21C	1.62E-06	4.29E-08	-7.65	8.6800392	-1.0934879
DNAAF3	2.90E-06	9.41E-08	-7.32	7.9030047	-1.09376866
LIPF	5.56E-06	2.32E-07	-6.95	7.0093775	-1.09377911
TMEM26	7.06E-07	1.34E-08	-8.15	9.8293794	-1.09473121
CCDC81	9.93E-04	2.03E-04	-4.33	0.339676	-1.09480577
DCTN1-AS1	2.66E-05	1.80E-06	-6.14	4.9861668	-1.09502308
C15orf32	3.27E-05	2.34E-06	-6.03	4.7256897	-1.09532487
NOP9	2.22E-07	2.64E-09	-8.86	11.4288111	-1.09540231
KRTAP13-1	3.26E-07	4.67E-09	-8.61	10.8662141	-1.09554375

TLR6	1.14E-06	2.64E-08	-7.85	9.1571448	-1.09579877
FBN2	9.59E-07	2.07E-08	-7.96	9.3989338	-1.0965314
SYNRG	6.54E-05	5.84E-06	-5.68	3.8221332	-1.09692396
SMIM6	6.82E-05	6.16E-06	-5.66	3.7703404	-1.09706417
FOXL2	2.39E-04	3.13E-05	-5.04	2.1696578	-1.09780731
SEPP1	9.63E-03	3.32E-03	-3.24	-2.3573644	-1.09799067
HLA-DPB2	6.49E-06	2.88E-07	-6.86	6.7971889	-1.09799464
TSPYL5	2.43E-04	3.20E-05	-5.03	2.1472156	-1.09800782
TIE1	1.01E-07	7.06E-10	-9.47	12.7235139	-1.0985295
PPP1R14A	1.98E-03	4.94E-04	-3.98	-0.5277567	-1.09924999
OR1L4	9.57E-05	9.53E-06	-5.49	3.3392452	-1.09970599
AOC1	3.47E-05	2.53E-06	-6	4.646979	-1.100025
SP5	8.45E-03	2.83E-03	-3.3	-2.2051831	-1.10042274
VIM	4.23E-05	3.29E-06	-5.9	4.3890595	-1.10045292
ACADL	1.64E-04	1.90E-05	-5.23	2.6581717	-1.10070117
SLPI	3.87E-02	1.75E-02	-2.54	-3.9085215	-1.10111292
TST	2.42E-04	3.17E-05	-5.03	2.1547432	-1.10142328
SCUBE1	7.52E-05	6.98E-06	-5.61	3.647064	-1.1014293
NRXN3	5.60E-06	2.35E-07	-6.95	7.0002131	-1.10182337
SPTBN5	1.33E-03	2.96E-04	-4.18	-0.0286526	-1.10198548
COQ2	1.14E-06	2.63E-08	-7.86	9.1634977	-1.10207226
TMEM155	2.78E-06	8.83E-08	-7.35	7.9660038	-1.10244472
CYP2A7	8.65E-06	4.20E-07	-6.71	6.4249447	-1.10276979
KCND3	2.00E-04	2.46E-05	-5.13	2.4036005	-1.10298235
OR1N2	2.40E-07	3.00E-09	-8.81	11.3029365	-1.10308113
LOC105379252///FAM95B1	2.81E-05	1.93E-06	-6.11	4.9145022	-1.10336817
LHX5	5.92E-06	2.54E-07	-6.92	6.9207662	-1.1034307
SCG3	2.05E-04	2.56E-05	-5.11	2.3675105	-1.10364673
KRTAP13-2	3.27E-05	2.34E-06	-6.03	4.7260845	-1.10366949
FAM110D	1.80E-07	1.90E-09	-9.01	11.753168	-1.10381376
OR6K2	2.86E-08	8.02E-11	-10.5	14.8501032	-1.10388527
EPS8	9.84E-04	2.00E-04	-4.33	0.3515174	-1.10410045
OTUD5	2.17E-03	5.55E-04	-3.94	-0.6388206	-1.10412472
MFSD6L	1.46E-05	8.41E-07	-6.44	5.7374605	-1.10520138
LOC100134317///LOC284412	5.34E-05	4.48E-06	-5.78	4.0841026	-1.10526714
PMP22	1.41E-04	1.56E-05	-5.3	2.8529937	-1.10573336
ZNF257	1.09E-05	5.77E-07	-6.59	6.109212	-1.10707609
CNTN6	3.12E-05	2.21E-06	-6.06	4.7802637	-1.10724294
SLC36A2	1.74E-06	4.72E-08	-7.61	8.5849738	-1.1074871
ECSCR	5.49E-08	2.45E-10	-9.97	13.7601801	-1.10766879
NPW	2.31E-04	3.00E-05	-5.05	2.2114012	-1.10770808
FAM149B1	1.01E-07	7.07E-10	-9.47	12.723028	-1.10798872
SPRY2	1.71E-05	1.02E-06	-6.36	5.5437539	-1.10853833
PGM5	2.27E-06	6.76E-08	-7.46	8.2299571	-1.10913751
GIF	1.63E-07	1.65E-09	-9.08	11.8913695	-1.10939577
KIAA1211	1.81E-07	1.94E-09	-9	11.7297612	-1.10939772
RHPN1	2.34E-05	1.53E-06	-6.2	5.1467941	-1.10958694
PDZD4	1.36E-07	1.23E-09	-9.21	12.1803683	-1.10961284
MAP9	4.62E-06	1.78E-07	-7.06	7.2707368	-1.10985053
GPR37L1	1.34E-04	1.47E-05	-5.32	2.9141513	-1.11018458
PARVA	6.74E-05	6.07E-06	-5.66	3.7847986	-1.11047424
CAPN3	8.21E-05	7.78E-06	-5.57	3.5400434	-1.11048553
PPM1K	2.09E-04	2.63E-05	-5.1	2.3399505	-1.11093029
SIRPD	9.16E-05	8.99E-06	-5.51	3.3968232	-1.11114489

GJC1	7.17E-04	1.32E-04	-4.49	0.755588	-1.11115456
GSTM4	9.51E-06	4.78E-07	-6.66	6.2970816	-1.11253532
MYH1	3.38E-05	2.45E-06	-6.02	4.6805606	-1.11265347
THNSL2	4.36E-05	3.43E-06	-5.89	4.3491191	-1.11278196
LOC105377924	1.22E-03	2.65E-04	-4.22	0.0774016	-1.11298196
DNAH1	8.22E-06	3.94E-07	-6.74	6.4869571	-1.11299626
CYP46A1	1.65E-06	4.39E-08	-7.64	8.6559933	-1.11320135
LOC780529	4.31E-06	1.63E-07	-7.1	7.3598844	-1.11337188
CLIC5	1.58E-07	1.55E-09	-9.11	11.9506277	-1.11392958
CLIC3	9.21E-03	3.15E-03	-3.26	-2.3047807	-1.11409823
UPK1B	2.44E-07	3.05E-09	-8.8	11.2868906	-1.11411985
FAM162B	1.53E-05	8.95E-07	-6.41	5.6763232	-1.11415989
ZNF280D	1.04E-05	5.43E-07	-6.61	6.1705476	-1.1144562
LTBP4	1.70E-04	1.98E-05	-5.21	2.6177869	-1.11449468
F10	2.23E-06	6.56E-08	-7.47	8.2599238	-1.11476373
GATS	1.70E-06	4.60E-08	-7.62	8.6101283	-1.11638258
PIK3R6	2.59E-07	3.36E-09	-8.76	11.1910442	-1.11657553
SAXO1	4.13E-05	3.19E-06	-5.91	4.420565	-1.11679514
ERCC2	4.10E-07	6.36E-09	-8.47	10.5633934	-1.11685792
DNAH14	1.35E-07	1.21E-09	-9.22	12.1958877	-1.11721434
SERPINF1	4.42E-04	7.03E-05	-4.73	1.3748107	-1.11724426
PTGER3	4.46E-06	1.70E-07	-7.08	7.3159506	-1.1173897
RBPMS	2.34E-05	1.53E-06	-6.2	5.1485063	-1.11746254
S1PR1	1.71E-04	2.00E-05	-5.21	2.6075079	-1.11780611
CYBRD1	3.34E-05	2.41E-06	-6.02	4.6962185	-1.11791102
SYT10	8.79E-06	4.28E-07	-6.7	6.4042753	-1.11793723
CDH19	7.32E-04	1.36E-04	-4.48	0.729232	-1.11801082
KCNIP3	9.66E-04	1.95E-04	-4.34	0.3752461	-1.11839395
F2	4.63E-08	1.90E-10	-10.1	14.0096938	-1.11849549
MMP16	1.20E-03	2.59E-04	-4.23	0.1001107	-1.1193329
SERPIND1	8.10E-06	3.87E-07	-6.75	6.506227	-1.11960107
GPM6A	3.16E-04	4.51E-05	-4.9	1.8088618	-1.12026185
LGR5	7.11E-06	3.25E-07	-6.82	6.678407	-1.12079617
ZNF23	3.29E-04	4.77E-05	-4.88	1.7549202	-1.12136327
BTC	1.62E-03	3.85E-04	-4.08	-0.2838963	-1.12166196
GAL3ST4	3.65E-09	1.63E-12	-12.6	18.6161902	-1.12274477
KLK15	3.49E-06	1.22E-07	-7.22	7.6502494	-1.12295973
CCNO	2.54E-07	3.24E-09	-8.77	11.2257284	-1.12313212
INSL4	4.34E-06	1.65E-07	-7.09	7.3503338	-1.12313267
SLC22A8	1.73E-05	1.04E-06	-6.35	5.5297929	-1.12438959
ADRA2A	6.43E-05	5.72E-06	-5.69	3.8424283	-1.12459948
OR51E2	4.42E-06	1.68E-07	-7.08	7.3278609	-1.1246065
RBM23	2.29E-05	1.48E-06	-6.21	5.1795661	-1.12461889
PDLIM7	3.55E-04	5.31E-05	-4.83	1.6500383	-1.12476515
C6orf58	5.80E-05	4.98E-06	-5.74	3.980518	-1.12477391
LCT	4.33E-05	3.39E-06	-5.89	4.3601279	-1.12539984
KRT37	1.38E-06	3.47E-08	-7.74	8.8884216	-1.12573497
ECM2	2.04E-06	5.88E-08	-7.52	8.3681325	-1.12577677
CCDC187	8.83E-04	1.74E-04	-4.38	0.4896385	-1.1257982
LOC100130256	3.35E-04	4.92E-05	-4.86	1.725177	-1.12594291
GATA1	1.35E-06	3.31E-08	-7.76	8.9354214	-1.1260269
PHKA1	1.12E-07	8.69E-10	-9.37	12.5205977	-1.12648861
PDCD11	8.21E-08	4.92E-10	-9.64	13.078712	-1.12673996
MGAM	2.78E-05	1.90E-06	-6.12	4.9321688	-1.12724244

AQP1	3.62E-05	2.68E-06	-5.98	4.5900994	-1.12739684
GLRB	5.90E-04	1.03E-04	-4.58	1.0020413	-1.12750095
ERBB3	6.16E-05	5.39E-06	-5.71	3.9012127	-1.12798582
RAB28	1.92E-04	2.34E-05	-5.15	2.4564489	-1.12831546
ZNF711	1.37E-04	1.50E-05	-5.32	2.8909694	-1.12851377
HOXA11-AS	4.59E-04	7.37E-05	-4.71	1.3278362	-1.12855595
MICAL3	1.78E-05	1.08E-06	-6.34	5.4897885	-1.12912592
GDAP1L1	4.77E-07	7.82E-09	-8.38	10.3591827	-1.12928922
AGO4	3.76E-05	2.82E-06	-5.96	4.5423061	-1.12931161
SNX21	4.24E-08	1.60E-10	-10.2	14.1780489	-1.12933837
C20orf85	4.14E-05	3.20E-06	-5.91	4.4180734	-1.12935326
MLLT6	1.88E-04	2.27E-05	-5.16	2.4833872	-1.12953568
NCAM1	9.64E-09	1.40E-11	-11.4	16.5424858	-1.12967399
NBEA	2.89E-03	7.81E-04	-3.81	-0.9692926	-1.12997063
C8orf34	4.65E-05	3.72E-06	-5.85	4.2673486	-1.13062303
HOGA1	4.40E-06	1.67E-07	-7.09	7.3344267	-1.13114689
STK32B	5.28E-06	2.16E-07	-6.98	7.0819393	-1.13138361
ASRGL1	1.01E-02	3.51E-03	-3.21	-2.408946	-1.13161598
ENDOU	1.24E-04	1.32E-05	-5.36	3.0175198	-1.13166794
NTNG1	6.14E-09	6.96E-12	-11.8	17.2203332	-1.13199686
ADAM22	7.28E-06	3.35E-07	-6.8	6.6470638	-1.13205346
GRIA3	1.79E-04	2.13E-05	-5.18	2.545868	-1.13209067
PDGFRB	3.55E-04	5.31E-05	-4.83	1.6496643	-1.13233573
SMOC2	1.09E-02	3.84E-03	-3.18	-2.494628	-1.13258855
C3orf18	1.20E-06	2.84E-08	-7.83	9.087909	-1.13259371
C3orf49	1.14E-04	1.19E-05	-5.41	3.121332	-1.13296224
NUDCD3	5.87E-06	2.51E-07	-6.92	6.9323057	-1.13296673
ZNF491	8.59E-05	8.30E-06	-5.54	3.4755004	-1.13405107
FXYD6	1.50E-07	1.44E-09	-9.14	12.0265891	-1.13473592
GPRC5B	2.50E-04	3.31E-05	-5.01	2.1151159	-1.13475857
EVC2	9.31E-06	4.65E-07	-6.67	6.3239281	-1.13557594
TRDMT1	1.22E-04	1.29E-05	-5.37	3.0410133	-1.13563082
CTSK	3.44E-03	9.61E-04	-3.73	-1.1694014	-1.13614413
HS6ST1	1.63E-06	4.35E-08	-7.64	8.6657863	-1.13635638
ATP8B3	2.82E-05	1.94E-06	-6.11	4.9116144	-1.13642666
TCTE3	1.14E-07	8.92E-10	-9.36	12.4949671	-1.13722651
C15orf59	4.02E-03	1.16E-03	-3.65	-1.3514541	-1.1373445
IFT46	5.54E-05	4.68E-06	-5.76	4.0408689	-1.13814099
TDGF1	1.13E-04	1.17E-05	-5.41	3.1341073	-1.13841569
SLITRK4	6.05E-06	2.62E-07	-6.9	6.8899109	-1.13854019
APEH	1.37E-06	3.41E-08	-7.75	8.9048811	-1.13863524
RGS11	1.36E-06	3.37E-08	-7.75	8.9174088	-1.13877437
HLX	7.87E-04	1.49E-04	-4.44	0.6373668	-1.13925146
C21orf2	2.60E-07	3.39E-09	-8.75	11.1812734	-1.13948631
DENND4A	9.77E-07	2.13E-08	-7.95	9.3712528	-1.13948866
C17orf64	6.44E-04	1.15E-04	-4.54	0.8912358	-1.14019915
GVINP1	1.39E-05	7.86E-07	-6.46	5.804033	-1.14119455
SDPR	7.80E-07	1.55E-08	-8.08	9.6844525	-1.14125873
GMDS-AS1	1.42E-04	1.58E-05	-5.3	2.8405265	-1.1412994
ATP8A2	6.08E-07	1.09E-08	-8.24	10.0313796	-1.14223056
NPY5R	1.07E-06	2.40E-08	-7.9	9.2534738	-1.14259921
THSD7A	8.17E-08	4.85E-10	-9.65	13.0915079	-1.14264852
SLC38A11	1.28E-05	7.12E-07	-6.5	5.902193	-1.14284347
LOC100129503	7.89E-07	1.58E-08	-8.08	9.6675951	-1.14360347

EPM2A	5.38E-04	9.08E-05	-4.63	1.123491	-1.14368641
ZNF585A	3.34E-08	1.03E-10	-10.4	14.6065731	-1.1445007
ZNF445	2.65E-05	1.79E-06	-6.14	4.9885364	-1.1448316
RYR3	2.43E-04	3.20E-05	-5.03	2.1469375	-1.14554112
GALNT16	1.09E-05	5.77E-07	-6.59	6.1097474	-1.14574294
PGLYRP3	3.61E-07	5.35E-09	-8.55	10.7325677	-1.14595801
CKB	4.71E-04	7.65E-05	-4.7	1.2921177	-1.14605589
DSPP	2.17E-04	2.75E-05	-5.08	2.2943305	-1.14637673
SRY	1.75E-07	1.81E-09	-9.04	11.7997401	-1.14677957
RAB3C	1.21E-04	1.29E-05	-5.38	3.0444525	-1.14721563
GRM5	6.21E-09	7.35E-12	-11.8	17.1679913	-1.14735743
SLC6A3	1.28E-04	1.38E-05	-5.35	2.9769023	-1.14764908
ST6GALNAC3	4.35E-06	1.65E-07	-7.09	7.3479583	-1.1483179
RS1	5.62E-06	2.36E-07	-6.95	6.9956721	-1.14832075
RAMP1	5.37E-03	1.64E-03	-3.52	-1.6844872	-1.14880735
KCNA7	2.86E-04	3.95E-05	-4.95	1.9403065	-1.14881967
SORCS3-AS1	7.71E-05	7.22E-06	-5.6	3.6138664	-1.14933123
SLFNL1	3.54E-07	5.22E-09	-8.56	10.7581775	-1.14954148
TMEM47	2.28E-05	1.47E-06	-6.21	5.1836677	-1.14955765
RIC8B	3.06E-06	1.01E-07	-7.29	7.8304293	-1.14994478
IGFBP6	1.01E-02	3.54E-03	-3.21	-2.4160289	-1.15002082
UPK2	5.85E-06	2.50E-07	-6.92	6.9381951	-1.15015604
REM2	1.51E-04	1.71E-05	-5.27	2.7654246	-1.15016835
MXRA7	3.27E-03	9.04E-04	-3.75	-1.1104545	-1.15021402
FAM228A	2.03E-06	5.81E-08	-7.52	8.3805159	-1.15039621
IGDCC4	1.39E-05	7.91E-07	-6.46	5.7985905	-1.15068996
RTN4RL1	1.28E-07	1.12E-09	-9.26	12.2711006	-1.15081083
KRT38	5.93E-09	5.80E-12	-11.9	17.3960573	-1.15096821
BTD	1.04E-05	5.40E-07	-6.61	6.1762397	-1.15101606
LRTOMT	7.62E-06	3.57E-07	-6.78	6.5848646	-1.15143288
SH2D3C	3.32E-04	4.85E-05	-4.87	1.7380809	-1.15161067
TAPT1-AS1	1.52E-04	1.72E-05	-5.26	2.7558345	-1.15177967
CDH22	1.01E-03	2.07E-04	-4.32	0.3188716	-1.15179045
ITGA11	4.05E-06	1.50E-07	-7.13	7.4444915	-1.15205857
SPERT	3.96E-04	6.11E-05	-4.78	1.5111791	-1.15216491
HSPA12B	3.65E-09	1.80E-12	-12.5	18.5230194	-1.1533443
LHFP	1.82E-03	4.46E-04	-4.02	-0.4275771	-1.15345871
MUC5B	1.80E-06	4.97E-08	-7.59	8.5345343	-1.15356266
BARHL1	2.86E-08	7.94E-11	-10.5	14.860188	-1.15364817
GLRA1	1.32E-04	1.44E-05	-5.33	2.9355625	-1.15366206
PAQR9	1.80E-07	1.91E-09	-9.01	11.7473795	-1.15371028
THRA	5.98E-05	5.19E-06	-5.72	3.9391462	-1.15393694
NBL1	2.60E-07	3.41E-09	-8.75	11.1767207	-1.15413975
KIR2DS1	4.15E-05	3.21E-06	-5.91	4.4137714	-1.15468109
TAF4B	2.00E-06	5.68E-08	-7.53	8.4028624	-1.15487305
POFUT1	1.40E-04	1.55E-05	-5.3	2.8616773	-1.15510169
ABI3BP	1.08E-03	2.26E-04	-4.28	0.2322015	-1.15524436
VGLL3	6.61E-06	2.95E-07	-6.86	6.77334	-1.15526093
FOXF1	1.08E-08	1.75E-11	-11.3	16.3276175	-1.15560359
LOC158863	2.17E-05	1.38E-06	-6.24	5.2465216	-1.15673895
C11orf86	1.03E-06	2.30E-08	-7.91	9.2946518	-1.15767382
GABRG2	5.53E-04	9.40E-05	-4.62	1.0899735	-1.157734
KCNS2	2.60E-07	3.39E-09	-8.75	11.1823029	-1.15822288
KRTAP7-1	7.42E-08	3.96E-10	-9.74	13.2913252	-1.15841936

KIAA1107	5.51E-04	9.35E-05	-4.62	1.095197	-1.15842928
MEGF8	1.29E-05	7.20E-07	-6.5	5.8903387	-1.15918167
LINC00466	7.91E-05	7.45E-06	-5.58	3.5826448	-1.15931335
C1QL2	8.88E-06	4.37E-07	-6.7	6.3850735	-1.15955631
LOC153682	2.19E-04	2.79E-05	-5.08	2.2807221	-1.16024283
ARHGDIG	2.51E-05	1.67E-06	-6.17	5.0584993	-1.16027683
PLB1	3.47E-04	5.14E-05	-4.85	1.6814954	-1.16107348
DIXDC1	6.09E-06	2.64E-07	-6.9	6.8817926	-1.16115932
NAT6	7.93E-07	1.59E-08	-8.07	9.6609988	-1.16132216
MYL3	9.77E-07	2.13E-08	-7.95	9.3726162	-1.16195182
LGALS8-AS1	2.57E-05	1.73E-06	-6.15	5.0268674	-1.16212082
RLN1	5.80E-06	2.47E-07	-6.93	6.9500354	-1.16283352
SLC7A14	4.63E-08	1.88E-10	-10.1	14.0188214	-1.16318577
CARD18	3.09E-07	4.32E-09	-8.64	10.9428204	-1.16339303
TMCC2	1.25E-08	2.07E-11	-11.2	16.1645084	-1.16402161
CPNE6	5.14E-05	4.27E-06	-5.8	4.1312534	-1.16450519
RALGAPA1	8.28E-08	5.03E-10	-9.63	13.0557597	-1.16490199
CDX4	1.55E-06	4.05E-08	-7.67	8.7357237	-1.1662749
SLC2A6	1.31E-04	1.43E-05	-5.34	2.9410638	-1.16661933
PKI55	2.45E-04	3.23E-05	-5.02	2.1388843	-1.16679603
MMACHC	1.58E-03	3.71E-04	-4.09	-0.2491077	-1.16687561
DLL4	4.22E-05	3.28E-06	-5.9	4.391669	-1.16688737
CCDC26	9.77E-07	2.13E-08	-7.95	9.3722645	-1.16695008
GSTM3	2.25E-05	1.45E-06	-6.22	5.2015543	-1.16713738
ZNF135	2.53E-04	3.36E-05	-5.01	2.0986269	-1.1671865
RAB17	1.10E-04	1.13E-05	-5.42	3.1684244	-1.16739506
HBG1	2.96E-05	2.07E-06	-6.08	4.8492471	-1.16833576
SUN3	3.00E-04	4.21E-05	-4.92	1.878075	-1.16884789
BNC2	5.47E-04	9.28E-05	-4.62	1.1026189	-1.16913978
PBX1	7.87E-03	2.61E-03	-3.33	-2.1264155	-1.16985395
FAM101A	5.64E-06	2.37E-07	-6.94	6.9899794	-1.17054161
GDNF	9.34E-08	6.24E-10	-9.53	12.8456102	-1.17064046
LINC00663	7.27E-06	3.35E-07	-6.8	6.6481714	-1.17089428
LOC101927770	2.57E-05	1.72E-06	-6.15	5.0299027	-1.17096202
MSC	6.74E-05	6.07E-06	-5.66	3.7851406	-1.17133785
LOC101927021	3.30E-05	2.37E-06	-6.03	4.7113252	-1.17168549
C3orf36	9.52E-04	1.92E-04	-4.35	0.3937517	-1.17204883
UBE2A	1.80E-04	2.14E-05	-5.18	2.5423513	-1.17207494
FHL1	5.40E-06	2.22E-07	-6.97	7.0526116	-1.1721482
PRL	7.76E-08	4.42E-10	-9.69	13.1829125	-1.17237456
ZFPM2	8.39E-05	8.00E-06	-5.56	3.5117596	-1.17280178
ZBTB40	1.57E-06	4.10E-08	-7.67	8.7240093	-1.17286296
SECISBP2L	9.67E-05	9.66E-06	-5.48	3.325789	-1.17337347
TCEB3B	1.80E-07	1.90E-09	-9.01	11.7508302	-1.17343323
KCNIP2	1.40E-07	1.27E-09	-9.2	12.1490748	-1.17347158
COMT///ARVCF	4.16E-06	1.55E-07	-7.12	7.4108694	-1.17350715
ZBTB18	7.22E-05	6.63E-06	-5.63	3.6974772	-1.17398807
LINC00944	8.54E-05	8.23E-06	-5.55	3.4843793	-1.17399329
REG3G	2.22E-05	1.42E-06	-6.23	5.2177984	-1.17447532
LOC101928087	4.23E-06	1.59E-07	-7.11	7.3852525	-1.17447654
SHC3	4.95E-03	1.49E-03	-3.55	-1.5920555	-1.17479634
GRAP2	3.91E-07	5.90E-09	-8.51	10.6366631	-1.17510901
TEX33	1.85E-06	5.14E-08	-7.57	8.5009421	-1.17514521
TEK	1.59E-07	1.56E-09	-9.1	11.9464114	-1.17563473

POU3F4	2.52E-05	1.68E-06	-6.16	5.0538068	-1.1764264
TGM4	1.00E-06	2.21E-08	-7.93	9.3347812	-1.17661497
PTPRB	3.78E-04	5.76E-05	-4.8	1.5692602	-1.17667276
SEMA6A	5.60E-05	4.77E-06	-5.76	4.0229514	-1.17683836
GABRA4	1.85E-05	1.13E-06	-6.32	5.4432221	-1.17688711
C5orf66-AS2	7.62E-06	3.56E-07	-6.78	6.5864202	-1.17691015
CD248	4.98E-04	8.20E-05	-4.67	1.2237601	-1.17711501
CSN1S1	1.80E-08	3.44E-11	-11	15.6729775	-1.1773609
NUTM1	1.50E-04	1.70E-05	-5.27	2.7713708	-1.17757507
KCNJ5	5.25E-05	4.39E-06	-5.79	4.1038626	-1.17761351
GTF3C2	6.42E-07	1.18E-08	-8.2	9.9545816	-1.17786941
GP1BB	2.04E-06	5.87E-08	-7.52	8.3702828	-1.17821909
TRIM36	9.46E-07	2.02E-08	-7.97	9.421534	-1.1782383
OR4K1	5.74E-08	2.68E-10	-9.93	13.672904	-1.17831824
RASGRP1	3.02E-07	4.15E-09	-8.66	10.9843534	-1.17842054
LRRC4C	8.06E-06	3.84E-07	-6.75	6.5130577	-1.17883655
COX4I2	6.20E-07	1.12E-08	-8.22	10.0026446	-1.17885729
SLC34A2	1.00E-03	2.05E-04	-4.32	0.3259194	-1.17913075
SPACA5	6.54E-06	2.91E-07	-6.86	6.788166	-1.17929108
GABRB3	7.10E-05	6.50E-06	-5.64	3.7174451	-1.17978918
PRKAR2A	1.82E-04	2.17E-05	-5.18	2.5296121	-1.1799187
SPATA8	1.19E-06	2.79E-08	-7.83	9.1048405	-1.17993038
TPH2	7.90E-04	1.50E-04	-4.44	0.6337535	-1.18086511
ZNF473	1.19E-05	6.43E-07	-6.54	6.0029554	-1.18098344
PLCXD3	1.20E-05	6.56E-07	-6.53	5.9835821	-1.18115326
TCEAL1	3.49E-06	1.21E-07	-7.22	7.6517195	-1.18210781
CA3	3.05E-04	4.30E-05	-4.91	1.8559554	-1.18228681
GLT1D1	1.07E-04	1.10E-05	-5.44	3.197744	-1.18268189
NAXD	4.65E-08	1.93E-10	-10.1	13.9956861	-1.18284761
PLPP7	2.58E-07	3.34E-09	-8.76	11.1973598	-1.18285533
PFKFB3	3.31E-06	1.13E-07	-7.25	7.7190138	-1.18325299
KLHDC4	1.95E-04	2.38E-05	-5.14	2.4366723	-1.18333171
CDH7	6.88E-07	1.30E-08	-8.16	9.8618071	-1.1835365
KIF1A	8.35E-07	1.71E-08	-8.04	9.5903341	-1.18358654
P2RX7	6.01E-06	2.60E-07	-6.91	6.8998145	-1.18366261
CACNG2	2.81E-05	1.93E-06	-6.11	4.9181293	-1.18382341
CBX7	6.66E-06	2.98E-07	-6.85	6.7628102	-1.18453407
CYP4F22	4.97E-08	2.12E-10	-10	13.9027865	-1.18473962
ZNF311	6.46E-06	2.85E-07	-6.87	6.8068856	-1.18526435
PAX7	6.35E-08	3.05E-10	-9.87	13.546201	-1.18535597
CPED1	1.44E-06	3.67E-08	-7.72	8.8336991	-1.18560109
GABRD	7.24E-06	3.33E-07	-6.81	6.6535085	-1.18596188
LINC01101	3.65E-09	1.73E-12	-12.6	18.557395	-1.18599753
TTI1	6.70E-05	6.02E-06	-5.67	3.7933533	-1.18601676
USP54	1.44E-08	2.45E-11	-11.1	16.0026255	-1.18610127
SH3GL2	3.19E-06	1.07E-07	-7.27	7.7734487	-1.1865399
VAT1L	1.16E-03	2.49E-04	-4.25	0.1399121	-1.18686779
MGC24103	1.10E-03	2.32E-04	-4.27	0.2092902	-1.1868696
PIK3C2G	6.62E-06	2.96E-07	-6.85	6.7700464	-1.18694543
NR2F1-AS1	9.46E-07	2.02E-08	-7.97	9.4229193	-1.18701153
BTBD18	3.77E-04	5.73E-05	-4.81	1.5742996	-1.18715843
ATP6V0D2	6.63E-05	5.94E-06	-5.67	3.8061351	-1.18755402
TEX26	3.07E-07	4.26E-09	-8.65	10.9579663	-1.18757367
EMP1	1.90E-04	2.30E-05	-5.15	2.4716614	-1.18765177

HNRNPM	6.42E-05	5.71E-06	-5.69	3.8449942	-1.18825873
FAM222A-AS1	1.35E-05	7.58E-07	-6.48	5.8398692	-1.18923549
ANO4	6.76E-05	6.10E-06	-5.66	3.7800991	-1.19026221
LINC00261	6.41E-05	5.70E-06	-5.69	3.8466473	-1.19026633
CHIT1	1.25E-03	2.74E-04	-4.21	0.0466611	-1.19040815
TMEM198	2.34E-08	5.48E-11	-10.7	15.2206863	-1.19042274
BAIAP2L2	6.09E-07	1.10E-08	-8.23	10.0268637	-1.19118094
AMY1C	1.58E-03	3.71E-04	-4.09	-0.2480644	-1.19118906
MYO3A	2.26E-06	6.72E-08	-7.46	8.235806	-1.19136885
WDR78	9.81E-06	4.97E-07	-6.65	6.2569636	-1.19238846
ZNF177	5.18E-07	8.74E-09	-8.33	10.2492584	-1.1929287
DNAH12	7.23E-04	1.34E-04	-4.48	0.7454861	-1.19297112
WHSC1	2.07E-04	2.58E-05	-5.11	2.3569305	-1.19309918
CDH13	4.96E-03	1.49E-03	-3.55	-1.5941148	-1.19347401
DTX1	8.57E-06	4.14E-07	-6.72	6.4384533	-1.19389859
BEST3	2.96E-06	9.68E-08	-7.31	7.8749379	-1.1943306
ZNF521	8.28E-08	5.04E-10	-9.63	13.0549379	-1.19455289
ZNF346	1.36E-06	3.36E-08	-7.75	8.9211759	-1.19505401
PDE9A	5.82E-07	1.02E-08	-8.27	10.1001669	-1.19589514
NR3C2	2.75E-03	7.36E-04	-3.83	-0.9121827	-1.19619232
LOC101929680	6.45E-05	5.74E-06	-5.68	3.8388104	-1.19700031
SRMS	3.66E-06	1.30E-07	-7.19	7.5828527	-1.19739055
FGA	4.07E-06	1.51E-07	-7.13	7.4380924	-1.1975393
CPQ	3.70E-06	1.32E-07	-7.18	7.5692499	-1.19869065
HS3ST5	3.48E-07	5.12E-09	-8.57	10.777516	-1.1987476
MYO7B	1.65E-04	1.91E-05	-5.22	2.6535016	-1.19899659
RASD1	2.07E-03	5.23E-04	-3.96	-0.5814585	-1.19899769
ADD2	1.08E-07	8.01E-10	-9.41	12.6001642	-1.19931875
TUBB1	1.59E-04	1.83E-05	-5.24	2.6956137	-1.19970355
ACTN2	2.64E-05	1.78E-06	-6.14	4.9966598	-1.2001384
DNAJB2	1.86E-06	5.19E-08	-7.57	8.4912864	-1.20036435
LOC284112	1.59E-09	3.61E-13	-13.5	20.0552888	-1.20091324
KLHL3	4.16E-06	1.55E-07	-7.12	7.4077763	-1.20197644
SSC5D	7.10E-07	1.35E-08	-8.14	9.8207736	-1.20222787
DLGAP2	4.14E-04	6.48E-05	-4.76	1.4549974	-1.20273255
ANTXR2	7.10E-05	6.48E-06	-5.64	3.7195641	-1.20292079
PCDHGA3	2.48E-05	1.65E-06	-6.17	5.0737411	-1.20376355
GNAO1	3.25E-06	1.10E-07	-7.26	7.7461505	-1.20440828
CPO	2.08E-08	4.46E-11	-10.8	15.4216169	-1.20479855
RASSF8-AS1	1.78E-06	4.91E-08	-7.59	8.546159	-1.20487789
SLITRK5	1.86E-04	2.23E-05	-5.16	2.501233	-1.20545452
ZRANB3	2.05E-04	2.56E-05	-5.11	2.3671993	-1.20547826
PMEPA1	1.04E-03	2.14E-04	-4.3	0.2840796	-1.20557963
CA5BP1	4.66E-07	7.54E-09	-8.4	10.3951169	-1.20586997
FERD3L	3.74E-04	5.68E-05	-4.81	1.5829719	-1.20640474
IGF2	4.37E-03	1.29E-03	-3.61	-1.4502814	-1.20665998
SGTB	1.88E-07	2.04E-09	-8.98	11.6843272	-1.20742989
INSM2	5.31E-09	4.35E-12	-12	17.6735538	-1.20831704
EFHC1	5.37E-07	9.17E-09	-8.31	10.2017946	-1.20840872
CREB3L3	1.19E-03	2.56E-04	-4.24	0.110047	-1.20884296
TMEM135	1.33E-05	7.45E-07	-6.48	5.8576245	-1.21024594
TRUB1	2.22E-08	4.96E-11	-10.8	15.3173362	-1.21037324
PCSK6	1.37E-07	1.24E-09	-9.21	12.1708632	-1.21057531
CYP1A1	1.72E-06	4.65E-08	-7.62	8.5993442	-1.21084155

RTN1	1.82E-05	1.11E-06	-6.33	5.4634064	-1.21086458
ZNF853	3.70E-06	1.32E-07	-7.18	7.5669582	-1.21185713
PHOX2B	8.32E-09	1.14E-11	-11.5	16.7465728	-1.21187485
LOC100132356	2.37E-07	2.91E-09	-8.82	11.3335141	-1.21188619
JAZF1	3.45E-06	1.20E-07	-7.22	7.6658096	-1.21201554
HES2	4.34E-04	6.87E-05	-4.74	1.397252	-1.21213293
TPD52L1	8.31E-05	7.90E-06	-5.56	3.5243883	-1.2125138
ATP2A1	3.27E-05	2.35E-06	-6.03	4.7221031	-1.21368415
DKK1	1.43E-02	5.32E-03	-3.04	-2.8023037	-1.21375192
UST	3.25E-06	1.10E-07	-7.26	7.7482894	-1.21414167
TEKT3	1.29E-05	7.20E-07	-6.5	5.8908833	-1.2142499
PDLIM5	1.26E-04	1.36E-05	-5.35	2.9911597	-1.21432808
PMP2	2.38E-07	2.95E-09	-8.82	11.3202414	-1.21436084
RELN	1.11E-05	5.91E-07	-6.58	6.08646	-1.21453797
NDUFAF7	3.23E-05	2.31E-06	-6.04	4.7390049	-1.21453972
SSPN	4.61E-05	3.68E-06	-5.86	4.2777289	-1.21552985
OGG1	8.28E-08	5.04E-10	-9.63	13.0535113	-1.21570908
ANGPTL1	1.92E-04	2.34E-05	-5.15	2.4559696	-1.2159775
SCN7A	6.19E-06	2.70E-07	-6.89	6.8622216	-1.21621388
DENND2A	1.22E-07	1.02E-09	-9.3	12.3671324	-1.21632414
ARC	1.22E-06	2.89E-08	-7.82	9.0709416	-1.21638367
HCN1	2.56E-07	3.30E-09	-8.76	11.2084067	-1.21692302
CCDC8	8.43E-07	1.74E-08	-8.03	9.5719155	-1.21726499
RPRM	1.09E-04	1.12E-05	-5.43	3.1794132	-1.21805414
COL1A2	9.15E-03	3.12E-03	-3.26	-2.297181	-1.21850746
DTNA	2.92E-06	9.48E-08	-7.32	7.8961269	-1.21873747
SOCS2	8.53E-04	1.66E-04	-4.4	0.533392	-1.21932714
ITGA8	2.80E-04	3.84E-05	-4.96	1.9678337	-1.21986296
C8orf4	1.94E-05	1.20E-06	-6.29	5.3838226	-1.22014731
BDNF	3.17E-07	4.45E-09	-8.63	10.9155361	-1.2206788
TNRC18	3.03E-08	8.73E-11	-10.5	14.7678783	-1.22099883
ITGA9	1.80E-06	4.97E-08	-7.59	8.5349826	-1.22127478
SLC2A13	3.74E-08	1.25E-10	-10.3	14.4177018	-1.22138536
SYT15	7.96E-07	1.60E-08	-8.07	9.6516984	-1.22142613
TPCN2	2.33E-07	2.86E-09	-8.83	11.3503762	-1.22162455
LOC102606465	2.19E-08	4.84E-11	-10.8	15.3413727	-1.22181581
TPM1	1.99E-07	2.21E-09	-8.95	11.6029746	-1.22201038
ADRA1B	3.78E-06	1.37E-07	-7.17	7.5343052	-1.22218252
BSN-AS2	1.50E-04	1.69E-05	-5.27	2.7752395	-1.22234587
WDR25	8.34E-08	5.12E-10	-9.62	13.0385152	-1.22236499
FRRS1L	9.37E-06	4.69E-07	-6.67	6.3157438	-1.22309013
BLID	3.21E-08	9.44E-11	-10.4	14.6909112	-1.22317793
FILIP1L	7.79E-04	1.47E-04	-4.45	0.6504325	-1.22412871
CD79B	5.30E-06	2.17E-07	-6.98	7.0749622	-1.22418465
FABP12	2.56E-04	3.41E-05	-5	2.0845165	-1.22449769
ROBO2	1.71E-07	1.75E-09	-9.05	11.8350963	-1.22454391
GPR141	9.77E-07	2.13E-08	-7.95	9.3707096	-1.22485465
UPK1A-AS1	1.24E-04	1.32E-05	-5.37	3.0179388	-1.22530339
SCTR	1.20E-06	2.84E-08	-7.83	9.0882568	-1.22565952
FAM107A	7.55E-08	4.15E-10	-9.72	13.2455321	-1.22579827
SHANK3	3.92E-08	1.35E-10	-10.3	14.343686	-1.22622729
CBLN2	5.01E-05	4.13E-06	-5.81	4.1649848	-1.22641939
LRCH3	2.80E-07	3.77E-09	-8.7	11.0781347	-1.22642931
TEX12	4.46E-07	7.04E-09	-8.43	10.4624159	-1.22644366

OR7D2	1.28E-05	7.07E-07	-6.5	5.9091493	-1.22647075
ATAT1	2.62E-06	8.14E-08	-7.38	8.047115	-1.22800946
MAGOHB	1.33E-06	3.23E-08	-7.77	8.9587015	-1.22926143
PABPC4L	9.32E-09	1.34E-11	-11.4	16.5905357	-1.22929755
DIO3OS	1.34E-06	3.28E-08	-7.76	8.9458632	-1.22945787
C9orf47	2.96E-06	9.66E-08	-7.31	7.8768957	-1.23112259
KIAA1661	1.02E-05	5.25E-07	-6.62	6.2037058	-1.23271053
TSPEAR-AS2	1.08E-04	1.11E-05	-5.43	3.1917335	-1.23419989
KCNB2	8.40E-05	8.01E-06	-5.56	3.5108472	-1.23477714
C10orf126	2.17E-07	2.57E-09	-8.88	11.4565542	-1.23504639
BPIFB3	3.95E-08	1.38E-10	-10.3	14.3185129	-1.23523899
LINC01354	6.09E-05	5.30E-06	-5.72	3.9178251	-1.23599381
GRK1	1.51E-07	1.46E-09	-9.13	12.0111896	-1.2363248
CDK5R1	7.32E-06	3.38E-07	-6.8	6.6375879	-1.23743867
ZNF772	7.97E-08	4.68E-10	-9.67	13.1272243	-1.23770433
ULK2	1.73E-08	3.12E-11	-11	15.7694566	-1.23770795
SLC4A2	7.66E-07	1.51E-08	-8.1	9.7106014	-1.23801806
LOC642852	6.37E-08	3.07E-10	-9.87	13.539086	-1.23849889
ID4	7.35E-05	6.78E-06	-5.62	3.674873	-1.23908614
SEMA3D	6.54E-07	1.21E-08	-8.19	9.9270061	-1.24010034
LOC100505549	1.56E-04	1.80E-05	-5.25	2.7156038	-1.24046289
SLC17A1	1.24E-04	1.32E-05	-5.37	3.0193305	-1.24058032
CHRNA9	1.57E-04	1.80E-05	-5.25	2.711429	-1.24151961
GDPD4	1.70E-06	4.58E-08	-7.62	8.6146271	-1.24162658
ASIC2	3.33E-02	1.46E-02	-2.62	-3.7443689	-1.24273815
ITIH5	2.21E-04	2.83E-05	-5.07	2.2680373	-1.24284384
CCT8L2	4.99E-06	1.98E-07	-7.02	7.16933	-1.24285563
FRMPD4	2.91E-05	2.02E-06	-6.09	4.8705307	-1.24325496
HYDIN	2.07E-07	2.34E-09	-8.92	11.5490011	-1.24326997
AVPR1A	8.87E-08	5.70E-10	-9.57	12.9342464	-1.24372637
LINC01095	1.32E-05	7.39E-07	-6.49	5.8653321	-1.24379047
RPS4Y2	1.83E-06	5.08E-08	-7.58	8.512864	-1.24429315
ZNF704	1.78E-03	4.31E-04	-4.04	-0.3954947	-1.24477185
CACNA1G	1.80E-08	3.42E-11	-11	15.6803209	-1.24555257
PDZRN4	1.52E-04	1.72E-05	-5.26	2.7564	-1.24578768
CHRM3	5.48E-07	9.40E-09	-8.3	10.1782867	-1.24578796
FAM234B	1.78E-05	1.08E-06	-6.34	5.4926392	-1.24616076
SLC7A9	1.01E-07	7.18E-10	-9.46	12.7077943	-1.24670878
MXD1	1.17E-03	2.51E-04	-4.24	0.1291956	-1.24744422
U2AF1L4	7.50E-07	1.46E-08	-8.11	9.7441371	-1.24749578
SULT1E1	2.42E-03	6.30E-04	-3.89	-0.7625056	-1.24825283
PP13439	1.37E-04	1.50E-05	-5.32	2.8905775	-1.24847025
ACPP	4.14E-05	3.20E-06	-5.91	4.4153013	-1.24890203
SALL2	5.29E-03	1.61E-03	-3.52	-1.6666166	-1.24894272
RIMBP3	3.59E-05	2.65E-06	-5.99	4.6032966	-1.24915838
SOCS7	8.52E-08	5.28E-10	-9.61	13.0092342	-1.24953106
CCDC80	1.77E-03	4.30E-04	-4.04	-0.3914182	-1.2496537
HTR1F	8.62E-05	8.34E-06	-5.54	3.4715395	-1.25042731
ZNF396	1.00E-05	5.15E-07	-6.63	6.2225761	-1.25078703
ACE	7.18E-08	3.74E-10	-9.77	13.3460823	-1.25139412
DPYSL4	1.19E-05	6.44E-07	-6.54	6.0015842	-1.25166693
TCEANC2	3.38E-05	2.45E-06	-6.02	4.6804246	-1.25249871
FAM129C	7.77E-06	3.66E-07	-6.77	6.5607798	-1.25496989
TMOD1	1.87E-04	2.26E-05	-5.16	2.4889383	-1.2550598

SSX7	1.36E-06	3.34E-08	-7.76	8.9278163	-1.25561762
LTBP1	4.62E-05	3.69E-06	-5.86	4.2751804	-1.25723274
P2RY14	7.82E-04	1.48E-04	-4.44	0.6452696	-1.25807028
FGFR2	1.39E-04	1.54E-05	-5.31	2.8691159	-1.25811308
KAZALD1	1.69E-06	4.56E-08	-7.62	8.6198024	-1.25914846
FZD10-AS1	3.63E-06	1.28E-07	-7.19	7.5969304	-1.25936105
NF2	1.28E-07	1.11E-09	-9.26	12.279603	-1.25958624
C12orf66	3.28E-06	1.12E-07	-7.25	7.7302394	-1.26033229
FAM198A	1.63E-07	1.62E-09	-9.09	11.9057956	-1.26103783
DNM3OS	2.29E-06	6.87E-08	-7.45	8.2145112	-1.26120197
CYP3A43	3.41E-06	1.18E-07	-7.23	7.6813164	-1.26182274
KRTAP4-8	3.66E-06	1.30E-07	-7.19	7.5828452	-1.2632372
NFKBIB	1.94E-06	5.45E-08	-7.55	8.4424269	-1.26324286
ATP2B2	7.74E-06	3.64E-07	-6.77	6.5660303	-1.26502846
ZSCAN18	3.39E-05	2.47E-06	-6.01	4.6738666	-1.26515297
DOK1	4.62E-07	7.43E-09	-8.4	10.4098799	-1.26544239
ABL1	4.63E-07	7.47E-09	-8.4	10.4044873	-1.26585796
MAS1	6.87E-05	6.23E-06	-5.65	3.7592	-1.2669213
FZD4	4.27E-07	6.73E-09	-8.45	10.5067808	-1.26696706
ZNF570	1.55E-06	4.03E-08	-7.68	8.7409764	-1.26726854
ARHGEF10	8.81E-04	1.73E-04	-4.38	0.4925527	-1.26751902
LGI4	2.42E-10	2.76E-14	-15.1	22.4808191	-1.26818081
RCOR1	3.19E-06	1.08E-07	-7.27	7.7703562	-1.26929601
TSPYL6	1.55E-05	9.03E-07	-6.41	5.6666766	-1.2698964
PAX2	6.40E-06	2.82E-07	-6.87	6.8172594	-1.26990207
TCEAL2	2.83E-06	9.10E-08	-7.34	7.936165	-1.27026162
OR8D2	4.72E-07	7.67E-09	-8.39	10.3787703	-1.27040902
CEMIP	1.92E-06	5.40E-08	-7.55	8.451695	-1.27189041
VIPR1	1.09E-04	1.12E-05	-5.43	3.1795248	-1.27206987
PDE1A	3.22E-07	4.57E-09	-8.62	10.8894194	-1.2731569
SFTPA2	3.24E-06	1.10E-07	-7.26	7.7528281	-1.27386023
RSPH10B	1.98E-04	2.44E-05	-5.13	2.4127366	-1.27605212
RNF150	1.70E-05	1.02E-06	-6.36	5.5497665	-1.27655315
TAS2R43	9.99E-08	6.91E-10	-9.48	12.744581	-1.27665635
RPS6KA6	8.79E-08	5.60E-10	-9.58	12.9502006	-1.27699485
MRAP2	1.23E-05	6.72E-07	-6.53	5.9594836	-1.27775872
NR2F2-AS1	1.23E-05	6.77E-07	-6.52	5.9514323	-1.27875903
TRPM3	2.25E-06	6.67E-08	-7.47	8.2429355	-1.27895898
UBXN10	4.81E-05	3.90E-06	-5.83	4.220406	-1.27921105
RPE65	9.10E-06	4.52E-07	-6.68	6.3520379	-1.27964838
MYO15B	4.58E-09	2.92E-12	-12.3	18.0582322	-1.27980948
CT55	4.49E-04	7.18E-05	-4.72	1.3532488	-1.27997832
PRAMEF17	2.67E-07	3.53E-09	-8.73	11.141627	-1.28108673
DNAJC22	2.01E-08	4.11E-11	-10.9	15.5007436	-1.28146893
SLIT3	8.99E-07	1.87E-08	-8	9.4983336	-1.28266305
RAD51B	2.96E-04	4.12E-05	-4.93	1.8984504	-1.28273355
CLDN5	2.78E-08	7.42E-11	-10.6	14.9264733	-1.28303764
PIF1	3.00E-06	9.84E-08	-7.3	7.8593805	-1.28386983
KCNA6	2.62E-06	8.12E-08	-7.38	8.0493046	-1.28421753
LOC100506191	4.49E-05	3.55E-06	-5.87	4.3132464	-1.28474362
ROBO4	2.47E-07	3.11E-09	-8.79	11.2662217	-1.28506628
KLC1	3.93E-06	1.44E-07	-7.15	7.4831236	-1.28524608
KCNQ4	3.27E-06	1.11E-07	-7.25	7.7364663	-1.28570627
PCOLCE2	4.88E-04	7.99E-05	-4.68	1.2490474	-1.28610064

MASP1	6.62E-05	5.92E-06	-5.67	3.8086302	-1.28667868
ADCY4	1.20E-07	9.94E-10	-9.31	12.3880253	-1.28692746
KLHL17	1.24E-07	1.06E-09	-9.28	12.3252531	-1.28697856
RASL12	4.27E-07	6.73E-09	-8.45	10.5073613	-1.28725339
NFIC	1.87E-06	5.23E-08	-7.57	8.4845295	-1.28885619
ACSS3	6.32E-07	1.15E-08	-8.21	9.9754488	-1.28908184
SLC9B1	3.95E-08	1.37E-10	-10.3	14.3245722	-1.28947123
MIR99AHG	2.06E-05	1.29E-06	-6.27	5.3126209	-1.28950091
SOBP	2.96E-07	4.04E-09	-8.67	11.0100215	-1.29004891
OSR2	4.97E-04	8.19E-05	-4.67	1.2247844	-1.29007336
CRYAB	3.92E-04	6.04E-05	-4.79	1.5227128	-1.29014533
TMEM202	2.73E-05	1.86E-06	-6.12	4.9524997	-1.29019812
UGT2B7	2.10E-04	2.64E-05	-5.1	2.3372484	-1.29026542
LY6G6F	3.37E-06	1.16E-07	-7.24	7.6976012	-1.29214483
FBXL22	2.46E-06	7.52E-08	-7.42	8.1254952	-1.29218672
ZCCHC3	8.41E-07	1.73E-08	-8.04	9.5770949	-1.29337905
TGFBR3	1.09E-06	2.46E-08	-7.88	9.2267209	-1.29359749
OR2T8	3.47E-07	5.02E-09	-8.58	10.79595	-1.29360634
RSPO1	6.55E-05	5.86E-06	-5.68	3.8192453	-1.2940588
GABRA2	7.41E-04	1.38E-04	-4.47	0.7139898	-1.29408018
ZXDC	4.73E-04	7.67E-05	-4.69	1.2884421	-1.29423733
CRYGS	1.08E-06	2.44E-08	-7.89	9.2366922	-1.29554826
C11orf88	3.26E-05	2.34E-06	-6.03	4.7276128	-1.29574496
SAMD4B	5.05E-05	4.17E-06	-5.81	4.1552474	-1.29654216
UNC5C	2.80E-05	1.92E-06	-6.11	4.9219295	-1.29766762
UACA	3.98E-04	6.16E-05	-4.78	1.504411	-1.29793153
FMOD	5.13E-04	8.53E-05	-4.65	1.1845955	-1.29796952
HOPX	1.29E-03	2.86E-04	-4.19	0.0045141	-1.29926765
DGKB	8.76E-08	5.50E-10	-9.59	12.9681777	-1.29993973
ZNF439	9.96E-06	5.09E-07	-6.64	6.234795	-1.29999934
KCNIP4	5.19E-06	2.11E-07	-6.99	7.1060652	-1.30003638
TLL1	1.11E-05	5.87E-07	-6.58	6.0927797	-1.30039024
MATN2	1.88E-05	1.15E-06	-6.31	5.4240028	-1.30073507
OLFML3	1.98E-09	4.96E-13	-13.3	19.7529715	-1.30083892
BCHE	1.99E-07	2.22E-09	-8.94	11.6001238	-1.30096935
DDX17	1.79E-04	2.12E-05	-5.18	2.5517591	-1.30304033
PRKCE	1.32E-04	1.44E-05	-5.33	2.9304253	-1.30325855
SLURP1	3.97E-05	3.02E-06	-5.93	4.4724205	-1.3043694
SMAD9	1.41E-07	1.29E-09	-9.19	12.1361091	-1.30465415
CRYBB1	1.58E-07	1.54E-09	-9.11	11.9591799	-1.30517442
C22orf24	1.16E-04	1.22E-05	-5.4	3.0985517	-1.3075507
C7orf50	2.07E-07	2.33E-09	-8.92	11.5514686	-1.30838846
CPXM2	2.17E-07	2.57E-09	-8.88	11.4545759	-1.30850407
FBXO27	2.10E-05	1.32E-06	-6.26	5.2886854	-1.30870346
NT5E	3.03E-04	4.25E-05	-4.92	1.8680807	-1.30875315
FOCAD	8.67E-07	1.80E-08	-8.02	9.5378837	-1.30893191
CYYR1	7.77E-08	4.45E-10	-9.69	13.1752492	-1.30955696
NPY	2.86E-08	7.83E-11	-10.5	14.8732231	-1.31025199
LAMA3	6.32E-07	1.15E-08	-8.21	9.9781619	-1.31193343
NACAD	2.17E-07	2.55E-09	-8.88	11.464081	-1.31226537
CHD2	4.65E-04	7.51E-05	-4.7	1.3093245	-1.31316441
GPER1	3.64E-06	1.29E-07	-7.19	7.5919031	-1.31332549
GXYLT1	8.14E-07	1.65E-08	-8.06	9.6206411	-1.31387284
GLI1	7.28E-07	1.39E-08	-8.13	9.7892945	-1.31410628

HPGDS	1.55E-05	9.05E-07	-6.41	5.6644975	-1.31419778
LOC400655	3.53E-05	2.60E-06	-5.99	4.6228153	-1.31473085
CXorf36	5.11E-05	4.24E-06	-5.8	4.1386853	-1.31528862
TRPC1	5.03E-05	4.16E-06	-5.81	4.1585349	-1.31537346
SHISA6	1.18E-05	6.36E-07	-6.55	6.0130236	-1.31701951
RBM45	2.70E-08	6.87E-11	-10.6	15.0003727	-1.31848019
TMIGD3	9.31E-06	4.64E-07	-6.67	6.3249069	-1.31963549
KLK6	3.48E-07	5.10E-09	-8.57	10.7803217	-1.32057779
LOC100287166	1.53E-04	1.74E-05	-5.26	2.7489273	-1.32059257
CPS1	3.66E-05	2.72E-06	-5.97	4.5772281	-1.32107838
KCNF1	3.90E-08	1.33E-10	-10.3	14.3581037	-1.32157272
DPEP1	1.09E-05	5.73E-07	-6.59	6.1166698	-1.32289832
LTF	2.05E-03	5.17E-04	-3.97	-0.5716919	-1.32355742
LCN12	6.60E-08	3.32E-10	-9.83	13.4640776	-1.32371394
COL6A3	2.26E-07	2.71E-09	-8.85	11.4018551	-1.32420669
CYP1B1	2.24E-03	5.75E-04	-3.93	-0.6737282	-1.32470337
NANP	6.79E-07	1.27E-08	-8.17	9.8790351	-1.32638209
LINC01197	9.08E-06	4.50E-07	-6.69	6.3552082	-1.32640007
PLEKHG4B	1.63E-05	9.64E-07	-6.38	5.6027184	-1.3265915
CNNM1	5.57E-05	4.74E-06	-5.76	4.0287556	-1.32731986
C9orf135	2.37E-04	3.09E-05	-5.04	2.1813906	-1.32813051
LINC00635	1.30E-04	1.41E-05	-5.34	2.9528342	-1.32857734
SENCR	5.81E-07	1.01E-08	-8.27	10.1049894	-1.32948024
LINC01094	2.16E-04	2.74E-05	-5.09	2.3004715	-1.33030051
NR0B1	6.85E-06	3.10E-07	-6.84	6.7255206	-1.33117103
ADAM20	3.99E-06	1.47E-07	-7.14	7.4628678	-1.33118542
RFX1	7.93E-08	4.64E-10	-9.67	13.1359452	-1.33248839
GPR20	2.89E-08	8.16E-11	-10.5	14.8331857	-1.33398651
BICC1	5.32E-09	4.90E-12	-12	17.5585615	-1.33483662
EPHA3	1.27E-06	3.08E-08	-7.79	9.0073813	-1.33511983
PRR15	1.40E-02	5.17E-03	-3.06	-2.7748437	-1.33548035
KCNA5	8.80E-08	5.63E-10	-9.58	12.9455026	-1.33574537
CACNA1C	5.74E-06	2.42E-07	-6.93	6.9681883	-1.33626874
APOM	1.03E-04	1.04E-05	-5.46	3.2493698	-1.33681707
HSPB7	2.32E-07	2.81E-09	-8.84	11.3671189	-1.33685885
CHRDL2	1.88E-02	7.39E-03	-2.91	-3.1107779	-1.33778213
TYRP1	3.20E-08	9.31E-11	-10.4	14.7043606	-1.33786615
TENM3	6.85E-06	3.10E-07	-6.84	6.725621	-1.33933819
RSPO2	1.10E-07	8.37E-10	-9.39	12.5571746	-1.33979026
SOX17	1.12E-07	8.62E-10	-9.38	12.5285604	-1.34078533
MICU3	9.95E-06	5.08E-07	-6.64	6.2361154	-1.3441795
LEF1-AS1	5.31E-08	2.36E-10	-9.99	13.7977684	-1.34432773
CACNG5	4.57E-07	7.31E-09	-8.41	10.4251944	-1.3451838
C17orf51	1.32E-07	1.18E-09	-9.23	12.2230846	-1.34524565
PHC2	5.58E-06	2.33E-07	-6.95	7.0062365	-1.34537274
LOC101927958	1.61E-05	9.47E-07	-6.39	5.6199223	-1.34539241
ERICH3	7.62E-06	3.56E-07	-6.78	6.5870986	-1.34560964
PCDHGB2	5.50E-08	2.46E-10	-9.97	13.754351	-1.34569173
HAND2-AS1	3.71E-06	1.33E-07	-7.18	7.5648857	-1.34679311
EFHB	1.01E-05	5.21E-07	-6.63	6.2106812	-1.34724424
LOC105371352	6.91E-08	3.55E-10	-9.8	13.3968299	-1.34727676
CCDC74B	5.04E-03	1.52E-03	-3.55	-1.6111411	-1.34750111
RHOF	3.27E-07	4.69E-09	-8.61	10.8620592	-1.3477008
DACT3	4.43E-05	3.50E-06	-5.88	4.3283195	-1.34868011

FUT7	3.04E-04	4.28E-05	-4.92	1.8618337	-1.34887395
ZNF516	4.96E-05	4.07E-06	-5.82	4.1787161	-1.34941677
COL6A2	4.96E-06	1.96E-07	-7.02	7.1799819	-1.35043438
MATN3	2.24E-05	1.44E-06	-6.22	5.2063052	-1.35068393
HTR2C	6.14E-09	6.77E-12	-11.8	17.247811	-1.35092376
MAPK8IP1	7.68E-08	4.25E-10	-9.71	13.2223641	-1.35124066
PIP5K1B	1.15E-04	1.21E-05	-5.4	3.1046539	-1.35217082
SPHKAP	1.89E-05	1.16E-06	-6.31	5.4192583	-1.35259392
LINC00308	3.00E-06	9.81E-08	-7.31	7.8619505	-1.35352721
ADRA1A	7.93E-07	1.59E-08	-8.07	9.6586842	-1.35381737
DQX1	2.05E-05	1.29E-06	-6.27	5.3171711	-1.35419524
ADGRA2	4.92E-07	8.12E-09	-8.36	10.3216722	-1.35436711
SATB2	2.82E-05	1.94E-06	-6.11	4.9117789	-1.35562173
PLXNA4	1.28E-05	7.13E-07	-6.5	5.9001547	-1.35590588
SNTG2	1.40E-04	1.55E-05	-5.3	2.8618055	-1.35591915
C14orf79	2.47E-06	7.55E-08	-7.41	8.1214773	-1.35615453
SLC28A1	1.74E-06	4.72E-08	-7.61	8.5845365	-1.35632543
CTNNA2	2.44E-06	7.43E-08	-7.42	8.1370181	-1.35641252
DKK2	3.53E-08	1.16E-10	-10.3	14.4933947	-1.35734063
SOX1	2.25E-07	2.69E-09	-8.86	11.4090497	-1.35783559
C8orf22	1.10E-07	8.36E-10	-9.39	12.5580608	-1.35839439
NAP1L3	5.75E-06	2.43E-07	-6.93	6.9650066	-1.3606704
CYP2C9	2.06E-05	1.29E-06	-6.27	5.3113511	-1.36082759
MTBP	2.04E-05	1.28E-06	-6.27	5.3233009	-1.36089212
ITGBL1	3.15E-04	4.49E-05	-4.9	1.8135128	-1.36231273
LRRC2-AS1	1.68E-06	4.51E-08	-7.63	8.6302406	-1.36253502
TMTC1	2.55E-07	3.26E-09	-8.77	11.2207757	-1.36259373
CHST8	3.74E-06	1.34E-07	-7.18	7.5522958	-1.36267683
SARDH	1.07E-07	7.85E-10	-9.42	12.6196137	-1.36381535
FOXJ1	3.54E-06	1.24E-07	-7.21	7.6332059	-1.36420302
C8orf33	1.33E-06	3.25E-08	-7.77	8.9552152	-1.36423233
SMIM10L2B	6.05E-07	1.08E-08	-8.24	10.0435492	-1.3672372
FOXA2	4.10E-05	3.15E-06	-5.92	4.4308062	-1.36748068
DIO3	1.58E-05	9.27E-07	-6.4	5.6411456	-1.36840361
GARNL3	2.62E-06	8.16E-08	-7.38	8.0447274	-1.37017563
RERGL	7.65E-06	3.59E-07	-6.78	6.5791171	-1.37103235
CADPS	5.28E-09	3.91E-12	-12.1	17.7765383	-1.37205547
DPY19L2	5.13E-06	2.06E-07	-7	7.1271964	-1.37289013
ZFP41	2.01E-04	2.49E-05	-5.12	2.3948135	-1.37300685
PCOLCE	5.28E-06	2.16E-07	-6.98	7.0809303	-1.3733169
SPINK6	7.56E-03	2.48E-03	-3.35	-2.079398	-1.37477171
NUDT10	2.55E-07	3.27E-09	-8.77	11.2176026	-1.37643849
SERP2	2.72E-06	8.61E-08	-7.36	7.9912523	-1.37704837
PTPRS	5.79E-05	4.97E-06	-5.74	3.9812216	-1.37920628
FXYD1	7.80E-07	1.55E-08	-8.08	9.6823638	-1.38011894
RAMP2-AS1	2.89E-06	9.35E-08	-7.32	7.9091854	-1.38054787
PPP1R9A	6.56E-06	2.92E-07	-6.86	6.7842807	-1.3820064
TEX28	4.78E-06	1.87E-07	-7.04	7.2263929	-1.38317901
KCNMA1	5.15E-06	2.08E-07	-7	7.119148	-1.38468575
HAND2	2.29E-06	6.85E-08	-7.45	8.2177039	-1.3846925
MIR503HG	8.61E-05	8.32E-06	-5.54	3.4729353	-1.38525521
SEMA6D	6.59E-08	3.28E-10	-9.83	13.4735237	-1.38866378
TMEM169	8.87E-06	4.35E-07	-6.7	6.3895244	-1.38918494
ANKRD35	3.04E-04	4.27E-05	-4.92	1.862439	-1.38955269

LILRA5	1.75E-06	4.77E-08	-7.61	8.5752784	-1.39011875
OXGR1	9.57E-06	4.82E-07	-6.66	6.2881881	-1.39160584
EPX	1.49E-05	8.58E-07	-6.43	5.7177642	-1.39267098
HTN3	4.23E-06	1.59E-07	-7.11	7.383551	-1.3942055
BMPER	5.88E-06	2.52E-07	-6.92	6.9288913	-1.39445298
CAPN11	1.20E-06	2.81E-08	-7.83	9.0957146	-1.39452592
PDE2A	1.68E-05	9.99E-07	-6.37	5.5667083	-1.3971112
LOC100507311	8.84E-06	4.32E-07	-6.7	6.3953686	-1.39740597
REN	1.17E-06	2.73E-08	-7.84	9.1255435	-1.39905237
GLIPR1L2	4.52E-05	3.59E-06	-5.87	4.3019919	-1.40048355
GULP1	1.07E-04	1.10E-05	-5.44	3.202273	-1.40123022
NKPD1	5.44E-06	2.25E-07	-6.96	7.0406749	-1.40128729
CD34	8.64E-06	4.18E-07	-6.71	6.4279464	-1.40246864
GRIN2A	1.60E-04	1.84E-05	-5.24	2.6900891	-1.40252473
PKDCC	5.32E-09	4.97E-12	-12	17.5460534	-1.4038562
HMCN1	3.00E-06	9.86E-08	-7.3	7.8572562	-1.40399681
FAM227B	7.46E-06	3.46E-07	-6.79	6.6146618	-1.40474555
HBE1	3.00E-06	9.81E-08	-7.31	7.8621764	-1.40494583
AGT	2.23E-07	2.66E-09	-8.86	11.4218613	-1.40516868
SEMA7A	4.84E-05	3.94E-06	-5.83	4.2110255	-1.40759665
LRCH2	2.70E-06	8.51E-08	-7.36	8.0027291	-1.40938257
HSPA12A	2.47E-07	3.11E-09	-8.79	11.2688649	-1.40941553
DNAJB3	4.89E-07	8.07E-09	-8.37	10.3281449	-1.40945805
PHACTR2-AS1	5.87E-07	1.03E-08	-8.26	10.0854785	-1.40952254
TSPYL1	2.44E-05	1.61E-06	-6.18	5.0961627	-1.40982459
CACNB2	6.14E-09	6.72E-12	-11.8	17.25387	-1.41002469
LOR	5.14E-04	8.56E-05	-4.65	1.1810919	-1.41027828
DST	2.90E-06	9.39E-08	-7.32	7.9056476	-1.41113105
VSTM4	1.13E-05	6.00E-07	-6.57	6.0707351	-1.41459305
EBF1	9.91E-06	5.05E-07	-6.64	6.2416345	-1.42060513
LYPD2	1.28E-07	1.12E-09	-9.26	12.2743254	-1.42171426
ADIRF	1.91E-04	2.32E-05	-5.15	2.4622409	-1.42211769
CCL23	3.09E-07	4.31E-09	-8.65	10.9473108	-1.42312351
PRSS23	1.99E-05	1.24E-06	-6.28	5.3498215	-1.42395415
PRICKLE2	6.42E-07	1.18E-08	-8.2	9.9554758	-1.42404111
HTR6	4.06E-07	6.26E-09	-8.48	10.5785109	-1.42585732
ADAMTS5	2.68E-06	8.43E-08	-7.37	8.0124738	-1.42649459
ADAMTS19	1.06E-05	5.56E-07	-6.6	6.1473832	-1.4273697
RECK	2.80E-05	1.92E-06	-6.11	4.9238284	-1.42941312
NDN	5.94E-05	5.14E-06	-5.73	3.9477518	-1.42970637
RCAN2	1.45E-06	3.71E-08	-7.71	8.8218423	-1.42978892
KIAA1755	3.24E-05	2.31E-06	-6.04	4.7372787	-1.42999195
ZNF44	8.96E-05	8.75E-06	-5.52	3.4241809	-1.43041719
EYA1	2.77E-09	8.84E-13	-12.9	19.2018896	-1.43214776
DPT	2.57E-06	7.93E-08	-7.39	8.0727789	-1.43569914
LENEP	3.25E-06	1.10E-07	-7.26	7.748994	-1.43603278
KIAA0408	5.32E-09	5.09E-12	-12	17.522395	-1.43657782
COL21A1	2.14E-05	1.36E-06	-6.25	5.2609271	-1.43744187
POPDC2	6.39E-07	1.17E-08	-8.21	9.9622466	-1.43837118
GRPR	8.03E-08	4.73E-10	-9.66	13.1155217	-1.43890321
DACH1	5.69E-06	2.39E-07	-6.94	6.9799622	-1.43977903
HP	8.36E-04	1.62E-04	-4.41	0.5588222	-1.44099178
MSX1	1.07E-07	7.81E-10	-9.42	12.62462	-1.44100986
NDP	1.47E-06	3.79E-08	-7.7	8.8007371	-1.4430389

FOXP4	9.24E-05	9.10E-06	-5.51	3.3849569	-1.4433345
KIAA1456	7.53E-07	1.47E-08	-8.11	9.7353692	-1.44460592
CLEC1A	1.85E-05	1.13E-06	-6.32	5.4455774	-1.44637043
PI16	7.55E-08	4.15E-10	-9.72	13.2440702	-1.44637638
SERPINA11	2.78E-08	7.39E-11	-10.6	14.9296079	-1.44715934
PCYT1B	2.96E-07	4.05E-09	-8.67	11.0078157	-1.44775104
SSR1	4.10E-05	3.16E-06	-5.92	4.430234	-1.44861737
CRISP3	2.17E-07	2.55E-09	-8.88	11.4628224	-1.44961516
LOC84843	7.25E-08	3.79E-10	-9.76	13.3322223	-1.45010532
FAM9C	8.53E-09	1.18E-11	-11.5	16.7067818	-1.45183573
ROR1	4.55E-05	3.63E-06	-5.86	4.2922164	-1.4527026
TNS2	2.22E-08	5.00E-11	-10.8	15.3106291	-1.45388898
RAI2	1.38E-06	3.48E-08	-7.74	8.886908	-1.45996195
NCAM2	4.02E-07	6.15E-09	-8.49	10.5965018	-1.46191348
LRTM2	1.41E-06	3.55E-08	-7.73	8.8674452	-1.46236435
ABCG2	1.73E-08	3.11E-11	-11	15.7721998	-1.46244169
GNG11	7.10E-06	3.24E-07	-6.82	6.6807235	-1.46249422
GJA1	9.19E-04	1.83E-04	-4.36	0.438625	-1.4634062
TMEM231	7.63E-04	1.43E-04	-4.46	0.6779406	-1.4642316
MYLK	6.63E-04	1.20E-04	-4.53	0.8547401	-1.4671184
GPRASP1	1.81E-06	5.01E-08	-7.58	8.5252725	-1.46754061
NECTIN3	5.44E-05	4.58E-06	-5.77	4.062745	-1.46803123
CARMIL2	9.31E-06	4.65E-07	-6.67	6.324072	-1.46824332
SLC15A1	4.30E-08	1.64E-10	-10.2	14.1499691	-1.46885146
KANK2	1.80E-07	1.88E-09	-9.02	11.7612232	-1.47675224
RNF165	5.60E-08	2.53E-10	-9.96	13.7305332	-1.47968424
LOXL4	7.60E-04	1.43E-04	-4.46	0.6822279	-1.4810295
PRUNE2	1.36E-06	3.38E-08	-7.75	8.9137082	-1.48289735
RIT2	6.40E-08	3.10E-10	-9.86	13.5296037	-1.4849946
CHRM2	2.75E-05	1.88E-06	-6.12	4.9434443	-1.48552685
JAM2	6.42E-08	3.15E-10	-9.85	13.5129656	-1.48950869
NR2F2	3.50E-05	2.57E-06	-6	4.6328467	-1.49097771
HIF3A	6.09E-08	2.91E-10	-9.89	13.5919265	-1.50225004
CDR1	2.45E-08	6.07E-11	-10.7	15.1220339	-1.5035602
TRH	3.34E-08	1.03E-10	-10.4	14.6056999	-1.50895246
FGF13	4.22E-06	1.58E-07	-7.11	7.3885652	-1.50967652
GDF7	7.22E-10	9.86E-14	-14.3	21.2846846	-1.50996889
PIGR	4.58E-02	2.15E-02	-2.45	-4.0956138	-1.51150248
LOC100128164	1.82E-07	1.96E-09	-9	11.719922	-1.51236275
COX7A1	5.44E-06	2.25E-07	-6.97	7.0431184	-1.5210145
SPON1	2.08E-04	2.60E-05	-5.11	2.3493998	-1.52189721
ENPP1	8.79E-08	5.55E-10	-9.58	12.9604682	-1.52214903
FGF9	8.55E-08	5.33E-10	-9.6	12.999341	-1.52435172
NPFFR2	1.50E-07	1.42E-09	-9.15	12.0350992	-1.52444299
LRFN5	3.03E-08	8.77E-11	-10.5	14.763589	-1.5249176
PNMT	6.24E-07	1.14E-08	-8.22	9.9920101	-1.52898023
FGF7	1.23E-07	1.03E-09	-9.3	12.3530777	-1.5360342
THSD4	5.69E-05	4.87E-06	-5.75	4.0025123	-1.53692029
NOSTRIN	3.65E-09	1.83E-12	-12.5	18.5071324	-1.53968984
HIGD1B	2.60E-06	8.07E-08	-7.39	8.0556316	-1.5400094
ACTA2	5.69E-04	9.78E-05	-4.6	1.0512094	-1.54218688
HDX	6.78E-06	3.05E-07	-6.84	6.7409665	-1.55335751
FLRT2	8.71E-05	8.44E-06	-5.54	3.4590156	-1.55449712
MGAT3	4.33E-05	3.39E-06	-5.89	4.3610538	-1.55518154

PCDH11Y	4.25E-06	1.60E-07	-7.1	7.3784472	-1.55614601
PTGDS	6.95E-05	6.32E-06	-5.65	3.7445565	-1.55791128
ADAMTS9-AS2	7.14E-06	3.27E-07	-6.81	6.6727471	-1.56073471
CKMT2	2.34E-08	5.44E-11	-10.7	15.2273673	-1.56359115
GATA4	1.69E-06	4.57E-08	-7.62	8.6180864	-1.56366843
CYR61	3.68E-04	5.56E-05	-4.82	1.6047574	-1.56677783
PSMD5-AS1	7.55E-08	4.13E-10	-9.72	13.2494564	-1.57158971
PDK4	1.31E-04	1.42E-05	-5.34	2.9433223	-1.5746302
CILP	2.76E-04	3.77E-05	-4.96	1.9848799	-1.57471555
RDH12	1.05E-05	5.49E-07	-6.61	6.1597536	-1.58362761
ATP1A2	1.24E-07	1.05E-09	-9.29	12.334246	-1.58403849
KCNB1	3.91E-07	5.89E-09	-8.51	10.6386793	-1.58459961
MFAP4	1.12E-03	2.37E-04	-4.27	0.1881205	-1.58577427
ATP1B2	2.42E-10	2.49E-14	-15.1	22.5767844	-1.58688839
PAMR1	2.02E-05	1.27E-06	-6.27	5.3326052	-1.58778619
C1QTNF7	7.68E-08	4.31E-10	-9.7	13.2078431	-1.58855761
SLC12A3	5.74E-08	2.68E-10	-9.93	13.6728947	-1.58961296
MITF	4.10E-07	6.36E-09	-8.47	10.5635558	-1.59234844
LONRF2	1.58E-04	1.82E-05	-5.24	2.7031368	-1.60090403
ADAM6	3.19E-06	1.07E-07	-7.27	7.7747792	-1.60145891
SLC16A9	2.78E-06	8.84E-08	-7.35	7.9646483	-1.6101685
SCUBE2	6.35E-05	5.62E-06	-5.69	3.8608107	-1.61287942
RADIL	5.26E-06	2.15E-07	-6.98	7.0882512	-1.6139724
DDIT4L	1.10E-07	8.22E-10	-9.4	12.5747271	-1.61789966
TLCD2	2.52E-07	3.19E-09	-8.78	11.2417797	-1.6184121
TPRG1	7.30E-03	2.38E-03	-3.37	-2.0383434	-1.62060883
PDZRN3	1.42E-05	8.09E-07	-6.45	5.7756031	-1.62318383
PTN	1.03E-03	2.13E-04	-4.31	0.2908674	-1.63045664
EDNRA	1.97E-07	2.19E-09	-8.95	11.6143224	-1.63341576
ATP6V1C2	1.14E-04	1.19E-05	-5.41	3.1230423	-1.64140716
PID1	4.86E-07	8.01E-09	-8.37	10.3351275	-1.64835782
FBLN1	7.51E-05	6.97E-06	-5.61	3.6487534	-1.65675603
FAM189A2	4.06E-07	6.24E-09	-8.48	10.5810407	-1.66037309
MRGPRF	1.81E-07	1.95E-09	-9	11.7277903	-1.66307163
DCN	1.59E-04	1.83E-05	-5.24	2.6955979	-1.67317849
ASPA	2.31E-09	6.83E-13	-13.1	19.448066	-1.68533688
LMO3	2.64E-06	8.24E-08	-7.38	8.034631	-1.68709047
LIFR	2.81E-07	3.79E-09	-8.7	11.0730614	-1.68946792
TUBB2B	6.90E-06	3.12E-07	-6.83	6.7181258	-1.69093608
PDE8B	9.84E-04	2.00E-04	-4.33	0.3520498	-1.69764835
KDM4D	3.75E-06	1.35E-07	-7.17	7.5462576	-1.69815064
SLIT2	7.37E-07	1.42E-08	-8.12	9.7682267	-1.70108744
HOXA13	5.47E-04	9.26E-05	-4.62	1.1047893	-1.70658638
SYNPO2	1.37E-05	7.70E-07	-6.47	5.8242509	-1.7077608
ALOX12	2.14E-07	2.45E-09	-8.9	11.5015536	-1.7103634
RBM24	6.14E-09	6.26E-12	-11.8	17.322461	-1.71342068
TNXB	1.44E-07	1.33E-09	-9.18	12.1002881	-1.71498556
CXCL14	3.70E-03	1.05E-03	-3.69	-1.2562768	-1.72395305
ADRA2C	5.36E-04	9.03E-05	-4.63	1.1289921	-1.72425285
CLEC3B	2.60E-06	8.06E-08	-7.39	8.0560893	-1.72867282
ABCC8	4.17E-05	3.23E-06	-5.91	4.4066609	-1.74002737
ADCY1	1.80E-08	3.48E-11	-10.9	15.6622062	-1.74187965
CARMN	1.82E-06	5.05E-08	-7.58	8.5188085	-1.74220241
AOC3	8.32E-06	4.00E-07	-6.73	6.4734779	-1.74310157

PTGIS	9.81E-05	9.85E-06	-5.48	3.3064753	-1.74470199
KIAA1683	3.31E-08	1.00E-10	-10.4	14.6345448	-1.74981426
MYRIP	2.10E-06	6.06E-08	-7.5	8.3374528	-1.75546414
DES	1.51E-04	1.71E-05	-5.27	2.7649719	-1.76527818
NEFM	1.17E-02	4.18E-03	-3.14	-2.5748582	-1.77627789
PHF21B	2.12E-07	2.42E-09	-8.91	11.5159718	-1.78083891
SPARCL1	2.29E-04	2.95E-05	-5.06	2.225599	-1.78247946
C2orf40	7.92E-06	3.75E-07	-6.76	6.5364128	-1.78273616
HLF	7.19E-06	3.29E-07	-6.81	6.6640568	-1.78688211
FCGBP	6.50E-04	1.17E-04	-4.54	0.8797125	-1.78695568
MYOT	1.20E-07	9.91E-10	-9.31	12.3912493	-1.78767638
EPDR1	4.66E-07	7.53E-09	-8.4	10.3963809	-1.78810367
MRVI1	8.50E-08	5.24E-10	-9.61	13.0157869	-1.79181086
MEG3	2.70E-05	1.83E-06	-6.13	4.9668575	-1.80185698
EDN3	8.11E-04	1.55E-04	-4.43	0.599493	-1.80433144
SBSPON	2.27E-07	2.73E-09	-8.85	11.3970014	-1.80999265
SFRP1	6.47E-04	1.16E-04	-4.54	0.885852	-1.8130655
GFRA2	7.91E-06	3.74E-07	-6.76	6.5391882	-1.81440226
PLPP3	8.28E-06	3.97E-07	-6.74	6.4790825	-1.82235748
HSPB2	1.97E-07	2.18E-09	-8.95	11.6170633	-1.85065224
PLN	1.09E-05	5.75E-07	-6.59	6.1141096	-1.86062
NR2F1	3.34E-04	4.89E-05	-4.87	1.7304179	-1.86927223
MYH11	2.13E-04	2.69E-05	-5.09	2.3158009	-1.87556461
DCLK1	5.28E-09	3.68E-12	-12.1	17.8336325	-1.88351929
RUNX1T1	7.74E-07	1.53E-08	-8.09	9.697652	-1.88826542
TMPRSS11B	4.08E-04	6.34E-05	-4.77	1.4758561	-1.91674421
SCARA5	3.65E-09	1.26E-12	-12.7	18.8618478	-1.92194043
PCP4	1.07E-05	5.57E-07	-6.6	6.144424	-1.9480622
AGAP11	4.42E-03	1.30E-03	-3.61	-1.4635363	-1.95774658
LCE3D	2.84E-03	7.65E-04	-3.81	-0.9495532	-1.96294424
IGF1	2.97E-04	4.13E-05	-4.93	1.8953098	-1.97142387
KRTDAP	5.78E-03	1.80E-03	-3.48	-1.7706125	-1.9844887
CRCT1	2.44E-02	1.01E-02	-2.78	-3.4016374	-2.02023957
PGR	8.23E-07	1.67E-08	-8.05	9.6082463	-2.06938789
SCGB3A1	2.75E-06	8.75E-08	-7.35	7.9746253	-2.09974196
EMX2	3.14E-06	1.05E-07	-7.28	7.7967257	-2.10209359
CXCL12	5.58E-05	4.75E-06	-5.76	4.027557	-2.11389547
ESR1	2.42E-05	1.59E-06	-6.18	5.105945	-2.11441872
ACTG2	5.78E-04	1.00E-04	-4.59	1.0283945	-2.12571415
MAL	4.43E-04	7.03E-05	-4.73	1.374028	-2.13766544
COL14A1	6.29E-06	2.75E-07	-6.88	6.8423242	-2.14518687
SPINK5	1.21E-02	4.39E-03	-3.12	-2.620372	-2.16080846
EMCN	6.30E-09	7.60E-12	-11.7	17.135916	-2.17155446
CGNL1	1.32E-08	2.23E-11	-11.2	16.0947763	-2.18323761
TFF3	1.41E-04	1.56E-05	-5.3	2.8528662	-2.19151201
NRG2	2.91E-07	3.96E-09	-8.68	11.0289101	-2.19704986
CNN1	1.23E-04	1.32E-05	-5.37	3.020965	-2.21244779
SFRP4	1.66E-03	3.95E-04	-4.07	-0.3093034	-2.2234596
PPP1R3C	1.93E-06	5.42E-08	-7.55	8.4481889	-2.25424282
KRT1	2.01E-03	5.06E-04	-3.97	-0.5501693	-2.31096051
AR	3.40E-05	2.48E-06	-6.01	4.6699078	-2.32174239
EMX2OS	2.29E-06	6.84E-08	-7.46	8.2192487	-2.33969782
CFD	4.86E-05	3.96E-06	-5.83	4.2064165	-2.38535045
KLK12	9.62E-03	3.32E-03	-3.24	-2.3559719	-2.48793409

IGFBP5	3.62E-05	2.68E-06	-5.98	4.591508	-2.49244679
SCGB1D2	3.27E-06	1.11E-07	-7.25	7.7365221	-2.51049294
SCGB2A1	1.09E-04	1.13E-05	-5.42	3.1709872	-2.54488006
WISP2	7.88E-06	3.72E-07	-6.76	6.5448347	-2.58168919
OGN	1.80E-07	1.92E-09	-9.01	11.7422314	-2.5955891
CRNN	1.06E-03	2.20E-04	-4.29	0.2582782	-2.77977032
SCGB1D1	3.90E-06	1.42E-07	-7.15	7.4949348	-2.91215389
**up-regulated genes**					
OSGIN2	1.42E-06	3.58E-08	7.73	8.8569373	1.00033603
CTSL	1.07E-03	2.23E-04	4.29	0.2454458	1.00045196
PAICS	2.77E-04	3.79E-05	4.96	1.9795416	1.0007526
EXOC6B	9.90E-08	6.83E-10	9.49	12.7564436	1.00087547
CLEC2B	4.62E-03	1.37E-03	3.59	-1.5137571	1.00093496
ZNF165	1.02E-03	2.10E-04	4.31	0.303018	1.00141671
TRBV5-4	3.04E-03	8.29E-04	3.78	-1.0269414	1.00144438
NUP155	7.29E-06	3.36E-07	6.8	6.6447052	1.00153484
TAGAP	2.09E-05	1.32E-06	6.26	5.2901015	1.00194797
FAM199X	3.25E-06	1.10E-07	7.26	7.7450696	1.00257974
CHEK1	1.18E-04	1.24E-05	5.39	3.0769782	1.00420697
MYO1B	3.49E-03	9.77E-04	3.72	-1.1859871	1.00457154
CBX3	2.29E-05	1.48E-06	6.21	5.1782621	1.00471753
LIN9	3.71E-06	1.33E-07	7.18	7.5638864	1.00708498
CPSF3	3.34E-05	2.41E-06	6.02	4.6960967	1.00721667
KLHL6	2.02E-04	2.50E-05	5.12	2.3878397	1.00736194
VPS29	1.50E-03	3.46E-04	4.12	-0.1825484	1.00807183
AMPD3	2.31E-04	2.99E-05	5.05	2.2138573	1.01153425
PSMC4	3.53E-04	5.27E-05	4.84	1.6577993	1.01154222
REL	2.02E-04	2.50E-05	5.12	2.3902153	1.01288986
SKA1	2.46E-06	7.51E-08	7.42	8.1259361	1.01335893
CYB5R1	2.05E-04	2.56E-05	5.11	2.3665943	1.01382392
HSPD1	5.18E-03	1.57E-03	3.53	-1.6418014	1.01398849
HIST1H2BK	1.57E-04	1.81E-05	5.24	2.7072985	1.01448604
PNPLA3	1.25E-03	2.74E-04	4.21	0.0449621	1.01453953
HIST1H2BB	3.74E-05	2.80E-06	5.96	4.549823	1.01531778
TUBA1C	4.73E-04	7.67E-05	4.69	1.2887522	1.01615595
ADGRG1	4.38E-05	3.44E-06	5.88	4.3438158	1.01673101
GK	4.77E-06	1.86E-07	7.04	7.2284056	1.01698539
RNASEH2A	2.47E-05	1.63E-06	6.17	5.0828713	1.01723271
RRM1	9.43E-05	9.36E-06	5.5	3.357774	1.01857421
ARHGAP11A	3.61E-06	1.27E-07	7.2	7.6046057	1.018888
ATP13A3	6.49E-06	2.88E-07	6.86	6.796233	1.01951844
ZWILCH	2.40E-05	1.58E-06	6.19	5.1161395	1.01953246
CORT	3.95E-05	3.00E-06	5.94	4.4817142	1.01999765
TOPBP1	6.05E-06	2.62E-07	6.9	6.8900048	1.02000655
NCBP2	5.94E-06	2.56E-07	6.91	6.9125681	1.02026125
HES1	2.14E-03	5.44E-04	3.95	-0.6204451	1.02032102
IGSF9	1.14E-03	2.42E-04	4.26	0.1674258	1.02103426
IRF5	4.01E-03	1.16E-03	3.65	-1.3494022	1.02211917
POU2AF1	4.57E-02	2.14E-02	2.45	-4.0931835	1.02271953
HIGD1A	7.67E-03	2.53E-03	3.35	-2.0958812	1.02355984
KRT18	2.91E-02	1.24E-02	2.69	-3.5954499	1.02362408
FOXM1	2.31E-05	1.50E-06	6.21	5.1669843	1.02375719
DENND1A	5.23E-04	8.76E-05	4.64	1.158551	1.02407555

RGS1	1.77E-02	6.88E-03	2.94	-3.042909	1.02428585
ARL11	1.32E-03	2.93E-04	4.18	-0.0185148	1.02541254
SDC1	3.45E-03	9.66E-04	3.72	-1.1743552	1.02555029
ZNF101	4.00E-06	1.48E-07	7.14	7.4577536	1.02604444
GNLY	1.95E-03	4.87E-04	3.99	-0.513181	1.02749974
PSMB2	1.34E-06	3.28E-08	7.76	8.9455266	1.02881176
POLR2H	6.60E-06	2.94E-07	6.86	6.7758206	1.02888082
TMPO	9.77E-07	2.12E-08	7.95	9.3737062	1.03055045
THOC3	6.83E-04	1.24E-04	4.51	0.8159991	1.03170718
MARVELD3	4.04E-06	1.49E-07	7.13	7.4469486	1.03197664
CXorf65	1.79E-04	2.13E-05	5.18	2.5492458	1.03253557
LMNB2	3.70E-05	2.76E-06	5.97	4.5640494	1.03309561
B3GNT3	9.82E-03	3.40E-03	3.23	-2.3794756	1.03344497
KIF15	7.80E-07	1.55E-08	8.08	9.6833338	1.03369406
GRP	3.61E-03	1.02E-03	3.7	-1.2276265	1.03383757
HIST2H2AC	3.70E-06	1.32E-07	7.18	7.566865	1.0339664
HTATIP2	1.97E-05	1.23E-06	6.29	5.3634835	1.03466198
SEH1L	8.39E-05	7.99E-06	5.56	3.5127896	1.03576292
BCL2L12	8.99E-06	4.44E-07	6.69	6.3691346	1.03738403
SPICE1	2.60E-07	3.38E-09	8.75	11.1848811	1.03833701
VRK1	5.01E-06	1.98E-07	7.02	7.1655705	1.03906317
MDH1B	5.65E-04	9.69E-05	4.61	1.0606123	1.03925736
ABCC5	1.15E-02	4.11E-03	3.15	-2.5593575	1.03933412
ITGA2	8.33E-04	1.61E-04	4.41	0.5631354	1.03940136
FUBP1	1.22E-05	6.67E-07	6.53	5.9672399	1.0398468
SECTM1	1.07E-03	2.25E-04	4.29	0.2390753	1.04013347
LOC100507639	3.02E-03	8.22E-04	3.79	-1.0189154	1.04112507
APOBEC3B	1.80E-03	4.40E-04	4.03	-0.4146233	1.04365056
CD247	1.48E-03	3.41E-04	4.13	-0.1673877	1.04405797
SAMD9	1.19E-03	2.58E-04	4.23	0.1047138	1.04559786
HLA-A	9.13E-05	8.95E-06	5.51	3.4010721	1.04661708
ACTB	5.73E-04	9.88E-05	4.6	1.0409549	1.04862211
PSMB3	2.25E-06	6.67E-08	7.47	8.2433037	1.04920192
TMPRSS4	2.75E-02	1.16E-02	2.72	-3.5330303	1.05136547
ALYREF	4.92E-05	4.03E-06	5.82	4.1896538	1.05140655
FOXE1	9.23E-05	9.08E-06	5.51	3.3869828	1.05169438
BMS1P6	2.65E-03	7.05E-04	3.85	-0.8703351	1.05311319
DHFR	9.78E-04	1.99E-04	4.33	0.3589869	1.05473626
PSAT1	6.67E-04	1.20E-04	4.52	0.8475695	1.0551173
SNRPA1	2.14E-04	2.71E-05	5.09	2.3111323	1.05542671
HIST1H2BE	5.69E-05	4.87E-06	5.75	4.0024517	1.05587681
EDARADD	2.20E-04	2.80E-05	5.08	2.2776962	1.05598659
ARMC8	3.19E-05	2.28E-06	6.04	4.7531402	1.05619044
ZNF683	4.11E-02	1.88E-02	2.51	-3.9745403	1.05639733
KIF18A	6.72E-07	1.26E-08	8.17	9.8920193	1.05757487
CBWD5	1.86E-03	4.59E-04	4.01	-0.455931	1.0580693
GPR68	2.95E-03	7.99E-04	3.8	-0.9920806	1.05812988
SDC4	1.20E-05	6.56E-07	6.53	5.9824191	1.0584517
TROAP	1.16E-06	2.70E-08	7.85	9.1384079	1.05900288
KIFC1	1.72E-05	1.03E-06	6.36	5.5360469	1.05978149
BCL3	7.17E-04	1.32E-04	4.49	0.7549873	1.06098332
SPINT2	2.04E-03	5.14E-04	3.97	-0.5656825	1.06104122
ELF4	4.07E-07	6.28E-09	8.48	10.5755983	1.06126785
HSPA1A	5.90E-03	1.84E-03	3.47	-1.7931631	1.06173794

PCK2	4.42E-05	3.49E-06	5.88	4.3312942	1.06257871
GPD2	5.54E-07	9.55E-09	8.29	10.1626713	1.0636099
TLR2	8.00E-07	1.61E-08	8.07	9.6448853	1.06505175
LAPTM5	1.13E-03	2.41E-04	4.26	0.1712273	1.06553997
ZNF90	2.44E-05	1.61E-06	6.18	5.0944189	1.06619363
HAUS8	1.28E-07	1.11E-09	9.26	12.2819235	1.06656215
RFC5	1.26E-05	6.97E-07	6.51	5.9228497	1.06656799
TTTY3	1.68E-04	1.96E-05	5.21	2.6297067	1.06661592
CCT5	1.18E-04	1.24E-05	5.39	3.0795474	1.06662591
PNP	5.54E-05	4.70E-06	5.76	4.0372228	1.06704381
KDF1	9.84E-08	6.77E-10	9.49	12.7656362	1.06726077
CENPQ	2.16E-05	1.37E-06	6.24	5.2525282	1.06746035
RPL39L	5.20E-04	8.69E-05	4.65	1.1662958	1.06751783
HIST1H3B	1.34E-05	7.54E-07	6.48	5.8452723	1.06798973
KIF4A	6.40E-08	3.12E-10	9.86	13.5243734	1.06829735
RELB	4.15E-05	3.21E-06	5.91	4.4142636	1.06917793
HIST3H2A	5.31E-06	2.18E-07	6.98	7.0713168	1.06971022
HLA-K	2.94E-05	2.05E-06	6.08	4.8553703	1.07001522
CD86	3.80E-05	2.86E-06	5.96	4.5281109	1.07075539
EPPK1	4.63E-03	1.38E-03	3.59	-1.5146184	1.0726458
S100A11	2.43E-04	3.19E-05	5.03	2.151201	1.07282505
FZD6	8.33E-05	7.92E-06	5.56	3.5217889	1.07325052
NDC80	2.26E-06	6.73E-08	7.46	8.2342327	1.07457145
DDX39A	3.87E-06	1.40E-07	7.16	7.5087056	1.07703354
BIRC3	4.98E-02	2.37E-02	2.4	-4.186771	1.07735652
DSC3	2.38E-02	9.78E-03	2.79	-3.3718637	1.07791399
PGK1	1.44E-06	3.66E-08	7.72	8.8350944	1.07853989
CDC7	1.00E-05	5.12E-07	6.63	6.2272696	1.08096659
LOC100128840	3.37E-05	2.44E-06	6.02	4.6840475	1.08256812
SNRPB	4.85E-06	1.90E-07	7.03	7.2065329	1.08327528
ACTL6A	1.52E-05	8.83E-07	6.42	5.6888107	1.08330844
NPL	1.92E-05	1.18E-06	6.3	5.4000896	1.08359518
IFI44	1.32E-02	4.84E-03	3.08	-2.7118694	1.08393872
FXYD3	2.54E-03	6.68E-04	3.87	-0.8194378	1.08410766
CHST11	2.82E-05	1.94E-06	6.11	4.9126513	1.08600531
HSPA6	4.46E-02	2.08E-02	2.46	-4.0660936	1.08622197
H2AFV	8.66E-07	1.79E-08	8.02	9.5404281	1.08672747
LIG1	2.05E-05	1.29E-06	6.27	5.3147936	1.08710945
HENMT1	1.06E-04	1.08E-05	5.44	3.2175778	1.08749005
NPVF	1.68E-04	1.95E-05	5.22	2.6333681	1.08774469
BST2	3.40E-05	2.47E-06	6.01	4.6726671	1.08799296
BBC3	1.47E-06	3.78E-08	7.7	8.8044252	1.08801254
DEFA6	4.41E-05	3.48E-06	5.88	4.3337572	1.08863633
C12orf54	6.80E-03	2.18E-03	3.4	-1.95727	1.0886931
PROSER2	7.51E-03	2.46E-03	3.36	-2.0710062	1.08944125
PTPRC	2.24E-03	5.76E-04	3.92	-0.6752395	1.09068692
PLOD2	1.07E-03	2.25E-04	4.29	0.2383361	1.09354693
ZC3H12D	1.11E-05	5.90E-07	6.58	6.0874591	1.09360339
RAB3IP	4.71E-05	3.80E-06	5.85	4.247186	1.09518901
GNB4	2.49E-06	7.62E-08	7.41	8.1122532	1.0959892
MAGOH	3.61E-06	1.27E-07	7.2	7.6070805	1.09612831
SERPINB5	7.77E-03	2.57E-03	3.34	-2.1112323	1.09651066
HIST1H2BG	5.22E-06	2.12E-07	6.99	7.0986051	1.09666619
PMAIP1	2.78E-06	8.86E-08	7.35	7.9623895	1.0973181

SLC31A2	3.78E-04	5.76E-05	4.8	1.5701145	1.09744927
MX2	1.15E-03	2.47E-04	4.25	0.148397	1.09784212
C2	5.79E-04	1.00E-04	4.59	1.0254738	1.09801333
PARP9	3.55E-04	5.31E-05	4.83	1.6488179	1.09971873
RCC2	7.34E-07	1.41E-08	8.13	9.778861	1.10018076
TNFAIP2	1.33E-04	1.46E-05	5.33	2.9195568	1.10060834
POLQ	2.70E-06	8.50E-08	7.36	8.0039706	1.10151015
DCUN1D5	2.51E-02	1.04E-02	2.76	-3.431317	1.10189134
DENND1C	2.21E-06	6.47E-08	7.48	8.2735607	1.10253423
CXCL13	3.43E-02	1.51E-02	2.6	-3.7741482	1.10261017
C1orf112	4.85E-06	1.90E-07	7.03	7.2082615	1.10493292
EPSTI1	2.48E-04	3.28E-05	5.02	2.1221003	1.10524637
FAAP24	1.07E-05	5.59E-07	6.6	6.1410105	1.10567254
RASSF4	4.04E-03	1.17E-03	3.65	-1.3589606	1.10583028
CKAP2	1.48E-05	8.54E-07	6.43	5.7216424	1.11128442
CD53	1.04E-03	2.15E-04	4.3	0.279487	1.11171669
FEN1	1.80E-07	1.89E-09	9.02	11.7581771	1.1132429
ACOT7	2.69E-04	3.64E-05	4.98	2.0196624	1.11418979
FCGR3B	7.57E-05	7.03E-06	5.61	3.6398704	1.11806916
SPOCD1	1.08E-04	1.12E-05	5.43	3.1830368	1.11863127
MGST1	4.97E-02	2.36E-02	2.41	-4.1828636	1.12050431
COL10A1	5.01E-04	8.27E-05	4.67	1.2158427	1.1213704
RMI1	6.89E-05	6.26E-06	5.65	3.7540416	1.12248761
VEGFA	7.47E-04	1.40E-04	4.47	0.7024059	1.12537174
PCNA	2.80E-06	8.95E-08	7.34	7.9530709	1.1263973
LGALS8	1.95E-06	5.51E-08	7.55	8.432142	1.12709154
PARP1	8.21E-08	4.90E-10	9.64	13.082484	1.12720168
KRT19	3.23E-02	1.41E-02	2.63	-3.7109456	1.12908016
CD96	3.49E-04	5.18E-05	4.84	1.6735138	1.12950324
CYTIP	2.24E-03	5.75E-04	3.93	-0.6737217	1.13037852
RBP1	7.57E-03	2.48E-03	3.35	-2.0800526	1.13111927
IL32	1.04E-04	1.06E-05	5.45	3.2311282	1.13114174
FOXD1	6.34E-04	1.13E-04	4.55	0.9109416	1.13298063
CD19	1.70E-04	1.99E-05	5.21	2.6129254	1.13444023
HS6ST2	1.19E-02	4.28E-03	3.13	-2.5955596	1.13448076
CHAF1A	2.47E-06	7.54E-08	7.41	8.1216436	1.13458714
UHRF1	1.23E-05	6.78E-07	6.52	5.9507491	1.13614075
TPM3	6.42E-07	1.18E-08	8.2	9.9557453	1.13729318
DEPDC1	1.20E-06	2.82E-08	7.83	9.0935651	1.13941432
MMP21	5.52E-05	4.66E-06	5.77	4.0456704	1.14016514
IRF6	2.84E-04	3.91E-05	4.95	1.9500665	1.14023189
LINC00152	5.09E-07	8.55E-09	8.34	10.2711537	1.14122848
HIST1H2BM	7.67E-06	3.60E-07	6.77	6.5764399	1.14158904
PSME2	1.33E-05	7.42E-07	6.49	5.8609534	1.14366225
CARNMT1	1.92E-05	1.19E-06	6.3	5.397966	1.14520622
HIST1H2BI	1.63E-06	4.33E-08	7.65	8.6713377	1.1452804
MCM3	2.23E-05	1.43E-06	6.23	5.2106837	1.14552219
CD300A	3.55E-04	5.32E-05	4.83	1.6477304	1.14591398
TIGAR	1.65E-05	9.75E-07	6.38	5.5913437	1.14770888
TUBB3	2.59E-04	3.46E-05	5	2.0699996	1.14940094
HRK	2.00E-03	5.00E-04	3.98	-0.5390118	1.15009657
MIR4435-2HG	6.49E-06	2.88E-07	6.86	6.79716	1.15083866
CCNE1	1.09E-06	2.45E-08	7.89	9.2326129	1.15107842
TNFRSF21	7.06E-05	6.44E-06	5.64	3.7268295	1.15190582

MRPL47	2.55E-05	1.70E-06	6.16	5.0389521	1.15473732
ARNTL2	1.59E-04	1.84E-05	5.24	2.6927103	1.15506045
HLA-G	3.06E-06	1.01E-07	7.29	7.8318871	1.15510944
G0S2	4.12E-03	1.20E-03	3.64	-1.3802804	1.15539263
E2F1	1.95E-05	1.21E-06	6.29	5.3779069	1.15635693
LDHA	4.75E-05	3.84E-06	5.84	4.2357438	1.15868579
MTHFD2	3.38E-06	1.16E-07	7.23	7.6931131	1.15963005
NMB	1.01E-02	3.50E-03	3.22	-2.4047462	1.16362657
MMP3	2.04E-02	8.12E-03	2.87	-3.1987902	1.16436312
SLC17A8	7.03E-07	1.33E-08	8.15	9.836422	1.16446697
ENO1	1.10E-04	1.14E-05	5.42	3.1622587	1.16656163
TNFRSF12A	2.00E-03	5.00E-04	3.98	-0.5390333	1.16793703
NCAPG	1.96E-06	5.55E-08	7.54	8.4254862	1.16902522
NCAPG2	4.15E-06	1.55E-07	7.12	7.4115562	1.1691711
MTHFD1	5.15E-06	2.09E-07	7	7.1164845	1.17184477
NFE2L3	5.69E-05	4.86E-06	5.75	4.0029555	1.17207448
KIF20A	1.96E-07	2.15E-09	8.96	11.6287886	1.17223428
PHF19	1.47E-06	3.78E-08	7.7	8.8053843	1.17225604
SGO1	6.52E-08	3.22E-10	9.84	13.4923019	1.17289419
HELLS	7.68E-08	4.28E-10	9.71	13.2132798	1.17340152
BATF	3.48E-04	5.16E-05	4.85	1.6773089	1.17476804
LAP3	8.16E-05	7.73E-06	5.57	3.5456719	1.17606177
TIPIN	1.73E-05	1.04E-06	6.35	5.5286919	1.17613468
CCL18	1.76E-02	6.85E-03	2.94	-3.0388377	1.17686288
MX1	3.89E-03	1.12E-03	3.67	-1.3134612	1.17715112
SPRY3	9.00E-07	1.88E-08	8	9.4925282	1.17904485
CIITA	2.28E-06	6.81E-08	7.46	8.2224956	1.18029516
IL23A	6.26E-04	1.11E-04	4.55	0.9264608	1.18305926
PRIM1	1.76E-06	4.82E-08	7.6	8.5650818	1.18881998
CDKN2D	5.58E-05	4.75E-06	5.76	4.0267487	1.1897405
HAVCR2	5.77E-04	9.98E-05	4.59	1.0311417	1.19007147
HIST2H3A///HIST2H3C	2.29E-05	1.48E-06	6.21	5.1764619	1.19057011
HCAR3	1.30E-03	2.89E-04	4.19	-0.0051658	1.19285951
BMS1P20	1.46E-02	5.44E-03	3.03	-2.8229909	1.19299658
DHCR24	1.28E-03	2.81E-04	4.2	0.0196238	1.19513435
DERL1	6.66E-07	1.24E-08	8.18	9.9038702	1.19618483
UGT1A6	6.43E-03	2.04E-03	3.43	-1.8932369	1.19699126
GMPS	3.66E-05	2.71E-06	5.98	4.5791204	1.19715001
LILRB3	1.16E-03	2.48E-04	4.25	0.1442294	1.1978996
TRAF3	5.74E-08	2.65E-10	9.94	13.682283	1.19796838
MCM10	1.74E-06	4.75E-08	7.61	8.5779185	1.19870644
HIST1H2BF	6.10E-06	2.66E-07	6.9	6.8775005	1.199058
DNAJC9	1.01E-07	7.23E-10	9.46	12.6999788	1.19935526
RECQL4	7.12E-07	1.36E-08	8.14	9.8163523	1.20010288
ADAT1	8.63E-06	4.18E-07	6.72	6.4295842	1.20065145
HIST1H2BN	5.10E-06	2.04E-07	7.01	7.1388599	1.20202299
CENPE	4.30E-08	1.64E-10	10.2	14.1534044	1.20286313
C20orf24	2.71E-05	1.84E-06	6.13	4.962825	1.20336675
C1QB	4.02E-03	1.16E-03	3.65	-1.3514269	1.20489655
SLC25A5	6.90E-06	3.12E-07	6.83	6.7174256	1.20550989
DUSP7	2.14E-02	8.63E-03	2.84	-3.2552293	1.20731868
IRF7	4.25E-05	3.31E-06	5.9	4.3829332	1.20873134
SLC16A3	9.28E-06	4.62E-07	6.67	6.3294275	1.20954366
IFT80	1.23E-06	2.95E-08	7.81	9.0495859	1.21021458

FAM24A	2.77E-03	7.42E-04	3.83	-0.9199722	1.21064785
BUB1	2.05E-06	5.92E-08	7.52	8.3615181	1.21263538
STK17A	3.00E-05	2.11E-06	6.07	4.8295657	1.21299869
CREB3L2	1.44E-04	1.61E-05	5.29	2.8253772	1.21324805
PBK	1.83E-04	2.19E-05	5.17	2.5179185	1.21655584
MARCH5	2.69E-04	3.65E-05	4.98	2.018837	1.21830027
EBP	2.96E-05	2.07E-06	6.08	4.8484678	1.21967622
CKS1B	1.28E-04	1.37E-05	5.35	2.980138	1.22124532
SLAMF7	1.42E-03	3.21E-04	4.15	-0.1098229	1.22203536
RCCD1	2.13E-05	1.35E-06	6.25	5.269165	1.22864955
IL21R	1.01E-04	1.02E-05	5.46	3.2694613	1.22878815
DONSON	9.14E-07	1.93E-08	7.99	9.470785	1.22937792
UBE2S	5.29E-05	4.43E-06	5.79	4.0947516	1.23045486
HILPDA	1.11E-02	3.92E-03	3.17	-2.513182	1.23245113
ADAM8	2.15E-04	2.72E-05	5.09	2.3065549	1.23288561
HMGB3P1	1.36E-03	3.05E-04	4.17	-0.0574516	1.23354584
KNSTRN	7.19E-06	3.30E-07	6.81	6.6637417	1.23499011
HK2	7.93E-04	1.51E-04	4.44	0.6292404	1.23635446
MCM5	7.78E-08	4.49E-10	9.68	13.1672951	1.2380667
IL4I1	3.91E-04	6.02E-05	4.79	1.5261023	1.23953188
TMEM132A	4.90E-06	1.93E-07	7.03	7.1935655	1.24018811
RIPK4	3.15E-03	8.64E-04	3.77	-1.0677874	1.24403642
PLSCR1	4.98E-05	4.09E-06	5.82	4.1731667	1.24914081
TCL1A	5.39E-04	9.11E-05	4.63	1.1208009	1.25231666
PPT1	5.84E-06	2.49E-07	6.92	6.9404207	1.25304582
IGHV3-48///IGHV3-69-1	3.20E-02	1.39E-02	2.64	-3.6975643	1.25482654
AMMECR1L	2.08E-04	2.60E-05	5.11	2.3512626	1.25507025
NAMPT	2.38E-04	3.10E-05	5.04	2.1772692	1.25721351
GBP1	1.28E-03	2.83E-04	4.2	0.014269	1.25973021
PTAFR	9.86E-05	9.91E-06	5.48	3.3011086	1.25998088
CEP152	2.19E-03	5.60E-04	3.94	-0.6487082	1.26074045
ITGA3	7.05E-05	6.43E-06	5.64	3.7283262	1.26080598
NCAPH	7.78E-08	4.50E-10	9.68	13.1661636	1.26281585
CXCL1	2.87E-02	1.23E-02	2.69	-3.5809089	1.26633649
KPNA2	2.97E-07	4.07E-09	8.67	11.0035607	1.26821013
HLA-C	3.85E-06	1.40E-07	7.16	7.513841	1.26945182
ABHD2	2.45E-04	3.23E-05	5.02	2.1388442	1.26990127
MCM7	5.06E-07	8.46E-09	8.35	10.281728	1.26999224
ISG15	1.17E-03	2.52E-04	4.24	0.1254075	1.27198301
IFI16	3.00E-05	2.10E-06	6.08	4.8309409	1.27371807
BLM	2.33E-07	2.85E-09	8.83	11.3543487	1.27475389
SLAMF8	2.21E-04	2.83E-05	5.07	2.2674364	1.27747902
MOCOS	6.54E-07	1.21E-08	8.19	9.928938	1.27758166
NFKBIE	3.05E-07	4.20E-09	8.66	10.970907	1.28048769
CCDC150	1.11E-05	5.87E-07	6.58	6.0929468	1.28051289
GLDC	2.80E-02	1.19E-02	2.71	-3.5511369	1.29199427
B2M	7.50E-06	3.49E-07	6.79	6.6074428	1.29383562
FAM49B	1.42E-06	3.59E-08	7.73	8.8556644	1.29620456
GRHL2	4.54E-03	1.35E-03	3.59	-1.4939539	1.29676186
DSN1	3.98E-07	6.05E-09	8.49	10.6118414	1.29897337
TFRC	1.96E-05	1.22E-06	6.29	5.371939	1.29930772
SLC20A1	5.26E-05	4.39E-06	5.79	4.1032871	1.30097841
HIST1H2BL	5.81E-06	2.48E-07	6.93	6.9465706	1.30112044
TNFRSF11B	1.12E-03	2.38E-04	4.26	0.1828545	1.3015873

COL8A1	2.94E-03	7.95E-04	3.8	-0.9870289	1.30285382
CTAG1A	1.35E-04	1.48E-05	5.32	2.9079304	1.30322791
KIF2C	2.04E-08	4.28E-11	10.8	15.4607656	1.30328746
TACSTD2	7.32E-03	2.39E-03	3.37	-2.0424325	1.30440579
HIST1H2BH	2.49E-06	7.64E-08	7.41	8.1093561	1.305418
PHLDA2	1.72E-03	4.13E-04	4.05	-0.3542619	1.31133108
ADGRE1	6.34E-04	1.13E-04	4.55	0.9095672	1.31274543
HMGB3	1.55E-03	3.62E-04	4.1	-0.2239088	1.31290963
ZC3H8	3.35E-05	2.42E-06	6.02	4.6927879	1.31775147
BRI3BP	6.78E-06	3.04E-07	6.84	6.7425264	1.31800179
CCL19	9.86E-03	3.42E-03	3.22	-2.3835874	1.32013621
RAB4B	9.76E-05	9.78E-06	5.48	3.3142673	1.3204444
GINS2	2.23E-06	6.55E-08	7.47	8.2607638	1.32119339
BCKDHB	4.08E-03	1.18E-03	3.65	-1.3679877	1.32270039
SNX10	3.67E-05	2.73E-06	5.97	4.5751933	1.32428875
MAD2L1	5.27E-06	2.15E-07	6.98	7.0854558	1.32672329
HIST1H2AD	3.74E-06	1.35E-07	7.17	7.5490585	1.32696878
ADM	1.88E-03	4.65E-04	4.01	-0.4677321	1.32869031
GZMB	6.66E-03	2.13E-03	3.41	-1.9336454	1.33100169
NUSAP1	4.12E-08	1.52E-10	10.2	14.2281498	1.33557304
LYPD1	2.41E-04	3.16E-05	5.03	2.1593923	1.33813032
SULF1	5.81E-04	1.01E-04	4.59	1.0211568	1.33821886
APP	7.67E-05	7.17E-06	5.6	3.6204539	1.33917278
SPC25	1.78E-06	4.90E-08	7.59	8.5487089	1.34130597
UBALD2	1.07E-06	2.41E-08	7.89	9.2499449	1.34161829
DTL	1.47E-07	1.37E-09	9.16	12.0699793	1.34313185
SNX5	4.71E-06	1.83E-07	7.05	7.2453292	1.34318201
CTSZ	6.90E-04	1.26E-04	4.51	0.8008856	1.34992593
RABL6	5.23E-04	8.77E-05	4.64	1.1578818	1.34998774
GBP4	4.98E-04	8.20E-05	4.67	1.2231251	1.35139894
LAMC2	3.16E-04	4.51E-05	4.9	1.8097166	1.35454117
IL36G	4.16E-02	1.91E-02	2.5	-3.9887392	1.35998983
PKMYT1	1.26E-06	3.02E-08	7.8	9.0256896	1.36037685
EGLN3	8.56E-04	1.67E-04	4.4	0.5277021	1.36178822
LTB	3.98E-05	3.03E-06	5.93	4.4706457	1.36245688
HIST2H2BD	1.36E-07	1.23E-09	9.21	12.1818144	1.36337985
H2AFZ	4.16E-07	6.50E-09	8.46	10.5415573	1.36483346
MCM2	8.79E-08	5.60E-10	9.58	12.9508488	1.36557258
LEMD1	1.28E-03	2.83E-04	4.2	0.0138243	1.36623003
BTN2A2	1.57E-04	1.80E-05	5.25	2.712568	1.36647966
GGH	1.37E-04	1.51E-05	5.31	2.8884602	1.36660129
LYZ	2.87E-03	7.75E-04	3.81	-0.9629811	1.36790062
MCM4	3.17E-06	1.06E-07	7.27	7.7814878	1.36848924
FANCA	9.65E-09	1.43E-11	11.4	16.5258909	1.3685269
FCGR1B	1.16E-03	2.48E-04	4.25	0.1434768	1.36867305
OLR1	4.12E-05	3.18E-06	5.91	4.4234284	1.37444006
HMGB2	1.74E-07	1.79E-09	9.04	11.8120864	1.37462067
KNL1	4.28E-06	1.62E-07	7.1	7.3676859	1.37781153
ANXA3	3.85E-03	1.10E-03	3.67	-1.3009114	1.38125563
SAT1	7.10E-06	3.24E-07	6.82	6.6807466	1.38471227
ATAD2	2.21E-03	5.66E-04	3.93	-0.6589442	1.38593992
COTL1	9.00E-07	1.88E-08	8	9.493266	1.38598063
TAP1	7.80E-05	7.32E-06	5.59	3.5993756	1.38728092
GMNN	6.62E-06	2.96E-07	6.85	6.7711492	1.38742342

TTK	5.15E-06	2.08E-07	7	7.1200209	1.39393142
MLF1	4.97E-04	8.17E-05	4.67	1.2270888	1.39413024
NCF2	4.19E-06	1.57E-07	7.11	7.3998877	1.39697415
IFI27	3.44E-04	5.10E-05	4.85	1.689299	1.40077082
NMI	1.43E-06	3.65E-08	7.72	8.8403467	1.40082006
GALNT14	1.39E-03	3.14E-04	4.16	-0.0880853	1.40177659
RAD51AP1	3.27E-06	1.11E-07	7.25	7.7359998	1.40246908
TRIB3	3.44E-04	5.09E-05	4.85	1.6915027	1.40457654
KIF11	1.41E-06	3.57E-08	7.73	8.8611575	1.41079866
CCNB2	3.91E-06	1.43E-07	7.15	7.4916198	1.41161719
KRTCAP3	5.92E-05	5.12E-06	5.73	3.9517534	1.41884006
LAMB3	7.64E-04	1.44E-04	4.46	0.6752936	1.41899446
CD3EAP	5.23E-05	4.36E-06	5.79	4.1111519	1.4191161
WARS	1.24E-05	6.81E-07	6.52	5.9459232	1.42096263
ISG20	2.61E-04	3.51E-05	4.99	2.057429	1.4231941
BID	7.65E-06	3.59E-07	6.78	6.5801562	1.4245294
SKA3	6.85E-06	3.10E-07	6.84	6.7250471	1.42617737
IER5	4.98E-05	4.09E-06	5.82	4.1733815	1.42632772
LRP8	6.88E-07	1.30E-08	8.16	9.8618888	1.42936019
OCIAD2	5.12E-06	2.06E-07	7	7.1307649	1.43610317
RSAD2	1.29E-03	2.85E-04	4.2	0.0084778	1.43755324
ABCA17P	3.41E-03	9.50E-04	3.73	-1.1591372	1.44016932
MIAT	8.30E-04	1.60E-04	4.41	0.5683284	1.4403979
SLC2A1	1.59E-03	3.73E-04	4.09	-0.2552812	1.44281981
HIST1H1D	5.44E-06	2.26E-07	6.96	7.0383827	1.44354853
HIST1H1E	3.38E-07	4.88E-09	8.59	10.8235662	1.45873825
KRT17	3.24E-03	8.95E-04	3.75	-1.1009344	1.45950538
PSMB9	1.75E-05	1.05E-06	6.35	5.5149223	1.46235608
MELK	3.02E-07	4.16E-09	8.66	10.9809931	1.4659398
OASL	2.81E-06	9.00E-08	7.34	7.9469785	1.46728643
CENPK	9.46E-07	2.02E-08	7.97	9.4226165	1.47148281
OIP5	3.25E-06	1.10E-07	7.26	7.7460304	1.47190216
MCM6	7.11E-09	9.14E-12	11.6	16.9575498	1.47468003
STYK1	1.21E-04	1.28E-05	5.38	3.0506986	1.47476783
CDKN2B	7.62E-06	3.56E-07	6.78	6.5887463	1.47540341
TICRR	1.13E-06	2.61E-08	7.86	9.1715862	1.47626609
CENPW	5.86E-05	5.06E-06	5.73	3.9641595	1.47696249
FAM26F	6.55E-04	1.18E-04	4.53	0.8710231	1.48057698
PATJ	1.22E-06	2.90E-08	7.82	9.0657787	1.48418793
FANCD2	4.10E-08	1.49E-10	10.2	14.2488316	1.48452164
KNTC1	5.32E-09	4.88E-12	12	17.5625716	1.48556332
NETO2	6.78E-08	3.42E-10	9.81	13.432751	1.48827151
CDC45	7.96E-07	1.60E-08	8.07	9.6545409	1.48854134
KIF18B	2.75E-07	3.68E-09	8.72	11.1014298	1.48901518
KRT7	4.03E-05	3.08E-06	5.93	4.4550204	1.49012032
CLDN1	2.08E-03	5.27E-04	3.96	-0.5902955	1.49451412
IGLL5	4.14E-04	6.48E-05	4.76	1.454986	1.49550498
RDH10	5.67E-03	1.75E-03	3.49	-1.7478925	1.4975735
GABRP	4.07E-03	1.18E-03	3.65	-1.365811	1.49876707
IFI44L	6.85E-04	1.25E-04	4.51	0.8109817	1.50119721
LAMP3	4.02E-04	6.22E-05	4.77	1.4941083	1.50232109
GPR87	7.74E-04	1.46E-04	4.45	0.6583556	1.50278372
RFC4	1.88E-07	2.04E-09	8.98	11.6804211	1.50556228
HMMR	1.94E-05	1.20E-06	6.3	5.3883629	1.50639916

FAM110A	4.95E-07	8.21E-09	8.36	10.3110113	1.51451487
MMP10	8.97E-03	3.04E-03	3.27	-2.2730915	1.51670381
ADAMDEC1	3.54E-04	5.29E-05	4.84	1.6535566	1.52019061
INHBA	2.59E-04	3.47E-05	5	2.0676306	1.52294841
FAM20C	1.51E-04	1.71E-05	5.27	2.7617658	1.52362303
UCK2	6.00E-07	1.06E-08	8.25	10.0583162	1.52546739
HN1	1.72E-06	4.65E-08	7.62	8.5995961	1.53509595
ELF3	3.98E-04	6.16E-05	4.78	1.5045689	1.53644604
MB21D1	4.49E-07	7.12E-09	8.42	10.4519112	1.5372083
TIMELESS	3.98E-07	6.06E-09	8.49	10.6109754	1.54167139
FAM83D	4.81E-05	3.91E-06	5.83	4.2189277	1.54442678
DLGAP5	1.59E-07	1.56E-09	9.1	11.9442941	1.54601815
EXO1	1.09E-06	2.46E-08	7.89	9.2280806	1.54679419
HIST1H2AE	7.37E-07	1.43E-08	8.12	9.7675316	1.54731806
WDHD1	2.54E-07	3.24E-09	8.77	11.2262596	1.54952259
S100P	2.22E-02	9.01E-03	2.82	-3.295156	1.55627737
RAD51	1.19E-07	9.80E-10	9.32	12.4018257	1.5578074
STMN1	2.28E-06	6.81E-08	7.46	8.2224001	1.55784541
WDR66	9.84E-04	2.00E-04	4.33	0.3520663	1.56243084
PIM2	2.71E-04	3.68E-05	4.97	2.0105427	1.56329534
LOC100506100	4.97E-07	8.28E-09	8.36	10.3032953	1.57649784
CDH3	3.43E-06	1.19E-07	7.23	7.6740857	1.58009239
HIST2H2AB	1.58E-07	1.55E-09	9.11	11.9550708	1.58494899
TNFSF10	1.45E-04	1.62E-05	5.29	2.8197037	1.6002987
CCR7	9.76E-05	9.78E-06	5.48	3.3144172	1.60411458
SMC4	6.91E-08	3.54E-10	9.8	13.399342	1.60554728
CCNB1	5.78E-06	2.46E-07	6.93	6.9543961	1.60615646
PRC1	1.30E-07	1.15E-09	9.24	12.2431308	1.60833804
CD70	4.35E-05	3.41E-06	5.89	4.3537707	1.62240309
CDCA2	2.78E-08	7.15E-11	10.6	14.9614328	1.62317426
BPNT1	1.74E-07	1.79E-09	9.04	11.8123691	1.62383857
CDC6	8.06E-06	3.84E-07	6.75	6.5131618	1.62557939
CDCA8	5.28E-09	4.21E-12	12.1	17.7056617	1.64407922
CXCL11	1.12E-03	2.37E-04	4.27	0.1885823	1.64757214
RACGAP1	8.87E-08	5.71E-10	9.57	12.9311643	1.65299926
GDF15	1.03E-02	3.62E-03	3.2	-2.4370085	1.65321603
MEI1	3.16E-04	4.52E-05	4.9	1.808641	1.65419694
IGHM	3.78E-05	2.84E-06	5.96	4.5342534	1.65744886
FCGR3A	1.19E-04	1.26E-05	5.38	3.06442	1.65973148
CDKN3	3.63E-06	1.28E-07	7.19	7.5956706	1.66179792
LMNB1	2.78E-07	3.73E-09	8.71	11.0885108	1.6731889
CEP55	1.20E-07	9.99E-10	9.31	12.3832538	1.67591443
ASPM	4.64E-08	1.91E-10	10.1	14.0028572	1.6789199
CENPN	3.61E-06	1.27E-07	7.2	7.6044671	1.67895614
CENPA	4.94E-05	4.05E-06	5.82	4.184284	1.69337327
CXCL8	8.67E-03	2.92E-03	3.29	-2.2349682	1.69619919
SOD2	1.82E-05	1.11E-06	6.33	5.465282	1.70589271
EFNA1	3.43E-06	1.18E-07	7.23	7.6753758	1.71308511
PLK1	7.11E-09	9.23E-12	11.6	16.948333	1.71323078
TFEC	9.12E-08	6.03E-10	9.55	12.8792244	1.71898263
CTSS	1.21E-06	2.87E-08	7.82	9.0763385	1.72607477
USP18	2.13E-04	2.68E-05	5.09	2.3194632	1.72728954
NEK2	3.79E-06	1.37E-07	7.17	7.5328813	1.72907538
RAD54L	2.40E-07	2.98E-09	8.81	11.3081173	1.73482202

HAGLROS	7.76E-04	1.47E-04	4.45	0.6543491	1.7350099
SPAG5	3.48E-08	1.09E-10	10.4	14.547152	1.7370533
HIST1H1C	5.61E-07	9.67E-09	8.29	10.1495444	1.74139487
IL2RA	6.27E-04	1.11E-04	4.55	0.9241119	1.74401294
SPP1	4.55E-03	1.35E-03	3.59	-1.4957935	1.75962601
TOP2A	9.93E-07	2.19E-08	7.94	9.3432451	1.76055126
ZNF587	1.24E-07	1.05E-09	9.29	12.3353086	1.76310972
KYNU	1.03E-05	5.32E-07	6.62	6.1909639	1.76635532
CKS2	1.18E-05	6.38E-07	6.55	6.0106338	1.77557804
GBP5	4.69E-04	7.59E-05	4.7	1.2993803	1.78466489
FOXA1	6.69E-03	2.14E-03	3.41	-1.9376631	1.78509979
CXCL10	5.83E-05	5.01E-06	5.74	3.973937	1.78679516
KIF23	7.94E-09	1.07E-11	11.6	16.8084617	1.7906285
FDCSP	3.31E-03	9.17E-04	3.74	-1.1251306	1.80705243
PLA2G7	5.60E-05	4.77E-06	5.76	4.02198	1.80712395
STAG3	1.23E-03	2.69E-04	4.22	0.06256	1.80751376
TPX2	5.21E-09	3.44E-12	12.2	17.899471	1.81017803
KLHDC7B	1.38E-04	1.52E-05	5.31	2.8778083	1.8149539
FANCI	5.74E-08	2.68E-10	9.93	13.6742456	1.82523447
PTTG1	1.37E-06	3.39E-08	7.75	8.9118403	1.83805
PTTG2	3.18E-06	1.07E-07	7.27	7.7790616	1.84112862
STAT1	1.69E-06	4.55E-08	7.63	8.6217583	1.84292037
TRIM59	3.22E-06	1.09E-07	7.26	7.7613402	1.84630915
TYMP	2.61E-07	3.43E-09	8.75	11.1712125	1.84709761
RRM2	3.26E-07	4.67E-09	8.61	10.868077	1.85174085
APOL6	2.59E-06	8.01E-08	7.39	8.062213	1.85710798
NUF2	1.43E-07	1.32E-09	9.18	12.1132434	1.86213643
SYCP2	6.10E-04	1.07E-04	4.57	0.9591291	1.87769194
ICAM1	2.63E-06	8.17E-08	7.38	8.0425727	1.87770099
ANLN	1.28E-07	1.12E-09	9.25	12.26686	1.87993784
S100A2	6.36E-03	2.02E-03	3.44	-1.8810481	1.88227848
HIST2H2AA3	3.50E-07	5.15E-09	8.57	10.7706749	1.88544993
UBE2T	3.26E-07	4.66E-09	8.61	10.8694853	1.89583251
TYMS	2.19E-08	4.84E-11	10.8	15.34277	1.90277918
MMP9	9.45E-05	9.38E-06	5.5	3.3555647	1.91984053
C1orf106	6.25E-06	2.73E-07	6.89	6.8499394	1.92514051
AIM2	1.90E-03	4.70E-04	4	-0.478595	1.93326453
EZH2	4.02E-07	6.15E-09	8.49	10.5954646	1.93642957
CDT1	4.31E-09	2.39E-12	12.4	18.248505	1.9597271
BIRC5	4.77E-07	7.80E-09	8.38	10.3612932	1.97013846
PCSK9	1.93E-03	4.80E-04	3.99	-0.4998335	1.99244721
CENPF	6.53E-07	1.21E-08	8.19	9.9321176	2.01132517
CENPU	9.84E-08	6.65E-10	9.5	12.782148	2.028893
KIAA0101	3.93E-07	5.94E-09	8.5	10.6297331	2.03351892
CA2	1.73E-05	1.04E-06	6.35	5.5300606	2.03859661
CDK1	5.11E-08	2.25E-10	10	13.8451182	2.0441458
IFI30	5.01E-07	8.35E-09	8.35	10.2951016	2.08746085
PLAU	1.44E-07	1.33E-09	9.18	12.1027126	2.09261826
ECT2	1.80E-08	3.43E-11	11	15.6778382	2.11003506
NEFH	7.12E-03	2.31E-03	3.38	-2.0090021	2.11482087
E2F7	5.30E-08	2.34E-10	10	13.8045599	2.12294062
TCAM1P	1.49E-04	1.68E-05	5.27	2.7788628	2.13425869
DBF4	1.45E-09	2.97E-13	13.6	20.2414117	2.14790276
FAM64A	3.24E-08	9.73E-11	10.4	14.6614538	2.19056431

LY6K	1.18E-07	9.57E-10	9.33	12.4254863	2.23958115
TK1	9.21E-07	1.95E-08	7.98	9.4557567	2.2968608
IDO1	1.43E-04	1.60E-05	5.29	2.8293396	2.33548308
CHAF1B	1.01E-07	7.02E-10	9.47	12.7289488	2.36090194
MMP12	1.24E-03	2.71E-04	4.21	0.0573173	2.36349789
APOC1	1.42E-04	1.58E-05	5.3	2.8420994	2.42631875
EDN2	5.79E-04	1.00E-04	4.59	1.0256749	2.46675924
MMP1	4.37E-04	6.93E-05	4.73	1.3890403	2.74960669
CXCL9	1.10E-04	1.14E-05	5.42	3.1649734	2.90007138
UBD	3.68E-06	1.31E-07	7.19	7.5777397	3.11269702
CDKN2A	4.45E-11	1.01E-15	17.3	25.5303492	3.70216996
