# Supplementary material for: Identification of Prognostic miRNA Signature and Lymph Node Metastasis-Related Key Genes in Cervical Cancer
Source: Front Pharmacol. 2020 May 8;11:544. doi: 10.3389/fphar.2020.00544 (PMC7226536; doi:10.3389/fphar.2020.00544)
Supplement: Supplementary file 5 [file Table_3.pdf]

Table S3. Results of differential expression genes analysis.

**down-regulated genes**				
PIP	DAPK1	WFDC1	GADD45G	ROBO4
MEGF10	C1orf105	CASZ1	GUCA1C	KLC1
ADCY7	DPYSL5	PXMP2	DKK4	KCNQ4
REG1A	KIT	PXK	AFF3	PCOLCE2
CCR4	PRKAR2B	PAPLN	PDE11A	MASP1
LINC01105	GREM2	TMPRSS6	SOX10	ADCY4
GLO1	CDK13	SRPX	C2orf54	KLHL17
HIST1H2AG	CCND3	RBPJL	TMEM132B	RASL12
BCAS3	MAGEA9	ZMAT4	PLEKHB1	NFIC
ZNF471	GDF11	C4BPA	UGT2A3	ACSS3
FZR1	PRAMEF8	PDE6G	CPB1	SLC9B1
EGFL7	LINC01556	ANKMY1	SH2D5	MIR99AHG
PRAME	ZBED5	NT5C1B	ATP1A1-AS1	SOBP
LINC01558	SEZ6L	MEOX2	RAPGEF4	OSR2
GUCY1A2	LOC284242	HYOU1	RORC	CRYAB
ZCCHC4	TMEM17	CFHR4	LCA5L	TMEM202
CLDN2	SNTG1	ZNF175	MATN4	UGT2B7
ACSM5	DAG1	ATOH8	WBSCR28	LY6G6F
CD300LG	C16orf45	STXBP5	FAM150A	FBXL22
CDON	FLRT3	USP51	RAPGEF3	ZCCHC3
C7orf69	FAIM2	CMTR2	LVCAT5	TGFBR3
DBF4B	LINC01314	KCNN3	MBLAC2	OR2T8
MMAB	NKAIN2	ACOX2	SLC4A4	RSPO1
KIF13A	SNHG25///SNORA50C	MYL1	LOC284244	GABRA2
HNF4G	KRTAP17-1	LOC441178	SLC6A2	ZXDC
ARHGAP6	TMEM14EP	BAAT	ANKH	CRYGS
SCAMP1	HDDC2	GCRG224	RBBP5	C11orf88
CA8	UNC93A	LINC01111	PRICKLE1	SAMD4B
MIR100HG	DLK1	SPI1	FAM19A5	UNC5C
CYP4F11	TMEM27	POLR3A	GPR173	RNF165
FAM86C2P	CCDC57	STAB1	SMIM2	LOXL4
ITPK1	RGAG1	HDLBP	TM4SF5	PRUNE2
PML	IFNA14	SEZ6	SLC19A3	RIT2
AP1S2	PCDHGB4	LINC00327	PLPPR2	CHRM2
UNC45B	PRKCDBP	CFAP43	ABRA	JAM2
DOK6	EPT1	FRAS1	PHACTR3	NR2F2
HTRA1	PAGE1	DNAH5	ESR2	HIF3A
EML6	OTP	CA5B	XYLT1	CDR1
RUNDC3B	LPAR1	EIF3L	WDR19	TRH
KCNG2	CTGF	SEPT8	LOC102723694	FGF13
SH3BGRL2	FOXO4	ERBB2	KNDC1	GDF7
PAQR7	RAB43	ZFP14	TPCN1	PIGR
LRRC4B	PAX3	KNG1	ROPN1	LOC100128164
LOC101060157	MUSTN1	IL10RA	TAS2R13	COX7A1
PLEKHA7	CALD1	DUSP8	GLIS3	SPON1
FLJ32742	PCK1	LTBP2	PLEKHS1	ENPP1
TRHDE	REV3L	KMT5A	CASP2	FGF9
ABCA6	CNIH3	CFC1	LYG2	NPFFR2
TMEM245	C14orf37	C16orf89	BTNL9	LRFN5
TGM2	WFIKKN1	ZNF76	HIST1H2AC	PNMT
TDH	NKX6-2	KRTAP9-8	CCL25	FGF7

AGER	CNTN2	SYTL4	WDR27	THSD4
NOP14	TCEAL5	ZSCAN21	TMEM204	NOSTRIN
LOC105374366	PLA2R1	TCAF2	HOXD13	HIGD1B
ZNRD1ASP	PRICKLE4	AGTR1	LONRF1	ACTA2
SPATA13	KCTD17	MAP2	CDH2	HDX
CCR10	MRO	SLC25A42	TAS2R50	FLRT2
TSPAN14	TMPRSS11F	OLFML1	ITIH6	MGAT3
PIGS	PXDN	ESM1	PCDHGC3	PCDH11Y
TERB1	FAM175A	FBXO22	SLC38A3	PTGDS
CCDC65	SPG20	PRSS37	ORAI2	ADAMTS9-AS2
GLP1R	CNRIP1	PTGS1	LOC101927598	CKMT2
ZNF765	KIF5C	CHRNE	TECTA	GATA4
TMEM92	DLC1	GRIA2	CELA1	CYR61
TTTY6	ZNFX1	PCDHB13	DUSP18	PSMD5-AS1
SNRPN///IPW	OR2H2	KCNN1	SP2	COL6A3
MBD5	C4orf22	FENDRR	PAPPA	CYP1B1
BCL7A	SH3PXD2A	GSTK1	ERAP2	NANP
USP22	GATC	CDH18	PHF23	LINC01197
SLC6A4	MTX3	MLXIPL	HEXA	PLEKHG4B
FGF2	SPON2	FAM81A	DBNDD1	CNNM1
SH3BP5	FRRS1	CAPN8	CCDC121	C9orf135
IQSEC3	ASCL1	LOC780529	C3orf49	LINC00635
CLU	FBXL7	CLIC5	NUDCD3	SENCR
SCN2B	METTL21C	CLIC3	ZNF491	LINC01094
LSAMP	DNAAF3	UPK1B	FXYD6	NR0B1
ADGRF5	LIPF	FAM162B	GPRC5B	ADAM20
CLDN10	TMEM26	ZNF280D	EVC2	RFX1
SPEF2	CCDC81	LTBP4	TRDMT1	GPR20
PLEKHA5	DCTN1-AS1	F10	CTSK	BICC1
MS4A2	C15orf32	GATS	HS6ST1	EPHA3
DMBX1	NOP9	PIK3R6	ATP8B3	PRR15
ZNF214	KRTAP13-1	SAXO1	TCTE3	KCNA5
GLDN	TLR6	ERCC2	C15orf59	CACNA1C
CPA4	FBN2	DNAH14	IFT46	APOM
MAN2C1	SYNRG	SERPINF1	TDGF1	HSPB7
TIMP3	SMIM6	PTGER3	SLITRK4	CHRDL2
TUFT1	FOXL2	RBPMS	APEH	TYRP1
FAM65C	SEPP1	S1PR1	RGS11	TENM3
TMSB4Y	HLA-DPB2	CYBRD1	HLX	RSPO2
SFTA2	TSPYL5	SYT10	C21orf2	SOX17
ARMCX1	TIE1	CDH19	DENND4A	MICU3
ICA1L	PPP1R14A	KCNIP3	C17orf64	LEF1-AS1
NPAS1	OR1L4	F2	GVINP1	CACNG5
CD200R1	AOC1	MMP16	SDPR	SYNPO2
F11-AS1	SP5	SERPIND1	GMDS-AS1	ALOX12
MAGEH1	VIM	GPM6A	ATP8A2	RBM24
DNAJC28	ACADL	LGR5	NPY5R	TNXB
HPCA	SLPI	ZNF23	THSD7A	CXCL14
BBOF1	TST	BTC	SLC38A11	ADRA2C
ZNF586	SCUBE1	GAL3ST4	LOC100129503	CLEC3B
GABRQ	NRXN3	KLK15	EPM2A	ABCC8
GRIP2	SPTBN5	CCNO	ZNF585A	ADCY1
SCARF2	COQ2	INSL4	ZNF445	CARMN
LOC100996455	TMEM155	SLC22A8	RYR3	AOC3

NXPH3	CYP2A7	ADRA2A	GALNT16	PTGIS
AMOT	KCND3	OR51E2	PGLYRP3	KIAA1683
KANSL1L	OR1N2	RBM23	CKB	MYRIP
PMEL	LOC105379252///FAM95B1	PDLIM7	DSPP	DES
CNTLN	LHX5	C6orf58	SRY	NEFM
CARNS1	SCG3	LCT	RAB3C	PHF21B
ST8SIA4	KRTAP13-2	KRT37	GRM5	SPARCL1
CD160	FAM110D	ECM2	SLC6A3	C2orf40
FGFRL1	OR6K2	CCDC187	ST6GALNAC3	HLF
IFNA21	EPS8	LOC100130256	RS1	FCGBP
CDK18	OTUD5	GATA1	RAMP1	MYOT
SLC6A6	MFSD6L	PHKA1	KCNA7	EPDR1
PRUNE1	LOC100134317///LOC284412	PDCD11	SORCS3-AS1	MRVI1
BMP6	PMP22	MGAM	SLFNL1	MEG3
BMP1	ZNF257	AQP1	TMEM47	EDN3
CDH5	CNTN6	GLRB	RIC8B	SBSPON
GDPD1	SLC36A2	ERBB3	IGFBP6	SFRP1
WBSCR17	ECSCR	RAB28	UPK2	GFRA2
DKK3	NPW	ZNF711	REM2	PLPP3
ERVW-1	FAM149B1	HOXA11-AS	MXRA7	HSPB2
MS4A6E	SPRY2	MICAL3	FAM228A	PLN
BEND6	PGM5	GDAP1L1	IGDCC4	NR2F1
C20orf197	GIF	AGO4	RTN4RL1	MYH11
POU3F1	KIAA1211	SNX21	KRT38	DCLK1
DNALI1	RHPN1	C20orf85	BTD	RUNX1T1
UBQLN3	PDZD4	MLLT6	LRTOMT	FOXA2
CDKN1C	MAP9	NCAM1	SH2D3C	DIO3
CRYBA2	GPR37L1	NBEA	TAPT1-AS1	GARNL3
FUT9	PARVA	C8orf34	CDH22	RERGL
SACS	CAPN3	HOGA1	ITGA11	CADPS
REG3A	PPM1K	STK32B	SPERT	DPY19L2
ZNF414	SIRPD	ASRGL1	HSPA12B	ZFP41
RBMS3	GJC1	ENDOU	LHFP	PCOLCE
RPL23AP32	GSTM4	NTNG1	MUC5B	SPINK6
LRRC17	MYH1	ADAM22	BARHL1	NUDT10
GIPC2	THNSL2	GRIA3	GLRA1	SERP2
CEP170	LOC105377924	PDGFRB	PAQR9	PTPRS
CCDC102B	DNAH1	SMOC2	THRA	FXYD1
LRRC7	CYP46A1	C3orf18	NBL1	RAMP2-AS1
KIR2DS1	CSN1S1	MYO7B	CD79B	PPP1R9A
TAF4B	NUTM1	RASD1	FABP12	TEX28
POFUT1	KCNJ5	ADD2	ROBO2	KCNMA1
ABI3BP	GTF3C2	TUBB1	GPR141	HAND2
VGLL3	GP1BB	ACTN2	UPK1A-AS1	MIR503HG
FOXF1	TRIM36	DNAJB2	SCTR	SEMA6D
LOC158863	OR4K1	LOC284112	FAM107A	TMEM169
C11orf86	RASGRP1	KLHL3	SHANK3	ANKRD35
GABRG2	LRRC4C	SSC5D	CBLN2	LILRA5
KCNS2	COX4I2	DLGAP2	LRCH3	OXGR1
KRTAP7-1	SLC34A2	ANTXR2	TEX12	EPX
KIAA1107	SPACA5	PCDHGA3	OR7D2	HTN3
MEGF8	GABRB3	GNAO1	ATAT1	BMPER
LINC00466	PRKAR2A	CPO	MAGOHB	CAPN11
C1QL2	SPATA8	RASSF8-AS1	PABPC4L	PDE2A

LOC153682	TPH2	SLITRK5	DIO3OS	PDK4
ARHGDIG	ZNF473	ZRANB3	C9orf47	CILP
PLB1	PLCXD3	PMEPA1	KIAA1661	RDH12
DIXDC1	TCEAL1	CA5BP1	TSPEAR-AS2	ATP1A2
NAT6	CA3	FERD3L	KCNB2	KCNB1
MYL3	GLT1D1	IGF2	C10orf126	MFAP4
LGALS8-AS1	NAXD	SGTB	BPIFB3	ATP1B2
RLN1	PLPP7	INSM2	LINC01354	PAMR1
SLC7A14	PFKFB3	EFHC1	GRK1	C1QTNF7
CARD18	KLHDC4	CREB3L3	CDK5R1	SLC12A3
TMCC2	CDH7	TMEM135	ZNF772	MITF
CPNE6	KIF1A	TRUB1	ULK2	LONRF2
RALGAPA1	P2RX7	PCSK6	SLC4A2	ADAM6
CDX4	CACNG2	CYP1A1	LOC642852	SLC16A9
SLC2A6	CBX7	RTN1	ID4	SCUBE2
PKI55	CYP4F22	ZNF853	SEMA3D	RADIL
MMACHC	ZNF311	PHOX2B	LOC100505549	DDIT4L
DLL4	PAX7	LOC100132356	SLC17A1	TLCD2
CCDC26	CPED1	JAZF1	CHRNA9	TPRG1
GSTM3	GABRD	HES2	GDPD4	PDZRN3
ZNF135	LINC01101	TPD52L1	ASIC2	PTN
RAB17	TTI1	ATP2A1	ITIH5	EDNRA
HBG1	USP54	DKK1	CCT8L2	ATP6V1C2
SUN3	SH3GL2	UST	FRMPD4	PID1
BNC2	VAT1L	TEKT3	HYDIN	FBLN1
PBX1	MGC24103	PDLIM5	AVPR1A	FAM189A2
FAM101A	PIK3C2G	PMP2	LINC01095	MRGPRF
GDNF	NR2F1-AS1	RELN	RPS4Y2	DCN
LINC00663	BTBD18	NDUFAF7	ZNF704	ASPA
LOC101927770	ATP6V0D2	SSPN	CACNA1G	LMO3
MSC	TEX26	OGG1	PDZRN4	LIFR
LOC101927021	EMP1	ANGPTL1	CHRM3	TUBB2B
C3orf36	HNRNPM	SCN7A	FAM234B	PDE8B
UBE2A	FAM222A-AS1	DENND2A	SLC7A9	KDM4D
FHL1	ANO4	ARC	MXD1	SLIT2
PRL	LINC00261	HCN1	U2AF1L4	HOXA13
ZFPM2	CHIT1	CCDC8	SULT1E1	DACH1
ZBTB40	TMEM198	RPRM	PP13439	HP
SECISBP2L	BAIAP2L2	COL1A2	ACPP	MSX1
TCEB3B	AMY1C	DTNA	SALL2	NDP
KCNIP2	MYO3A	SOCS2	RIMBP3	FOXP4
COMT///ARVCF	WDR78	ITGA8	SOCS7	KIAA1456
ZBTB18	ZNF177	C8orf4	CCDC80	CLEC1A
LINC00944	DNAH12	BDNF	HTR1F	PI16
REG3G	WHSC1	TNRC18	ZNF396	SERPINA11
LOC101928087	CDH13	ITGA9	ACE	PCYT1B
SHC3	DTX1	SLC2A13	DPYSL4	SSR1
GRAP2	BEST3	SYT15	TCEANC2	CRISP3
TEX33	ZNF521	TPCN2	FAM129C	LOC84843
TEK	ZNF346	LOC102606465	TMOD1	FAM9C
POU3F4	PDE9A	TPM1	SSX7	ROR1
TGM4	NR3C2	ADRA1B	LTBP1	TNS2
PTPRB	LOC101929680	BSN-AS2	P2RY14	RAI2
SEMA6A	SRMS	WDR25	FGFR2	NCAM2

GABRA4	FGA	FRRS1L	KAZALD1	LRTM2
C5orf66-AS2	CPQ	BLID	FZD10-AS1	ABCG2
CD248	HS3ST5	FILIP1L	NF2	GNG11
C12orf66	UACA	C17orf51	LOC100507311	GJA1
FAM198A	FMOD	PHC2	REN	TMEM231
DNM3OS	HOPX	LOC101927958	GLIPR1L2	MYLK
CYP3A43	DGKB	ERICH3	GULP1	GPRASP1
KRTAP4-8	ZNF439	PCDHGB2	NKPD1	NECTIN3
NFKBIB	KCNIP4	HAND2-AS1	CD34	CARMIL2
ATP2B2	TLL1	EFHB	GRIN2A	SLC15A1
ZSCAN18	MATN2	LOC105371352	PKDCC	KANK2
DOK1	OLFML3	CCDC74B	HMCN1	TMPRSS11B
ABL1	BCHE	RHOF	FAM227B	SCARA5
MAS1	DDX17	DACT3	HBE1	PCP4
FZD4	PRKCE	FUT7	AGT	AGAP11
ZNF570	SLURP1	ZNF516	SEMA7A	LCE3D
ARHGEF10	SMAD9	COL6A2	LRCH2	IGF1
LGI4	CRYBB1	MATN3	HSPA12A	KRTDAP
RCOR1	C22orf24	HTR2C	DNAJB3	CRCT1
TSPYL6	C7orf50	MAPK8IP1	PHACTR2-AS1	PGR
PAX2	CPXM2	PIP5K1B	TSPYL1	SCGB3A1
TCEAL2	FBXO27	SPHKAP	CACNB2	EMX2
OR8D2	NT5E	LINC00308	LOR	CXCL12
CEMIP	FOCAD	ADRA1A	DST	ESR1
VIPR1	CYYR1	DQX1	VSTM4	ACTG2
PDE1A	NPY	ADGRA2	EBF1	MAL
SFTPA2	LAMA3	SATB2	LYPD2	COL14A1
RSPH10B	NACAD	PLXNA4	ADIRF	SPINK5
RNF150	CHD2	SNTG2	CCL23	EMCN
TAS2R43	GPER1	C14orf79	PRSS23	CGNL1
RPS6KA6	GXYLT1	SLC28A1	PRICKLE2	TFF3
MRAP2	GLI1	CTNNA2	HTR6	NRG2
NR2F2-AS1	HPGDS	DKK2	ADAMTS5	CNN1
TRPM3	LOC400655	SOX1	ADAMTS19	SFRP4
UBXN10	CXorf36	C8orf22	RECK	PPP1R3C
RPE65	TRPC1	NAP1L3	NDN	KRT1
MYO15B	SHISA6	CYP2C9	RCAN2	AR
CT55	RBM45	MTBP	KIAA1755	EMX2OS
PRAMEF17	TMIGD3	ITGBL1	ZNF44	CFD
DNAJC22	KLK6	LRRC2-AS1	EYA1	KLK12
SLIT3	LOC100287166	TMTC1	DPT	IGFBP5
RAD51B	CPS1	CHST8	LENEP	SCGB1D2
CLDN5	KCNF1	SARDH	KIAA0408	SCGB2A1
PIF1	DPEP1	FOXJ1	COL21A1	WISP2
KCNA6	LTF	C8orf33	POPDC2	OGN
LOC100506191	LCN12	SMIM10L2B	GRPR	CRNN
ATF2	LRRC73	SCGB1D1		

up-regulated genes

OSGIN2	ARHGAP11A	SEH1L	SDC4	LAMB3
CTSL	ATP13A3	BCL2L12	TROAP	CD3EAP
PAICS	ZWILCH	SPICE1	KIFC1	WARS
EXOC6B	CORT	VRK1	BCL3	ISG20
CLEC2B	TOPBP1	MDH1B	SPINT2	BID
ZNF165	NCBP2	ABCC5	ELF4	SKA3

TRBV5-4	HES1	ITGA2	HSPA1A	IER5
NUP155	IGSF9	FUBP1	PCK2	LRP8
TAGAP	IRF5	SECTM1	GPD2	OCIAD2
FAM199X	POU2AF1	LOC100507639	TLR2	RSAD2
CHEK1	HIGD1A	APOBEC3B	LAPTM5	ABCA17P
MYO1B	KRT18	CD247	ZNF90	MIAT
CBX3	FOXM1	SAMD9	HAUS8	TOP2A
LIN9	DENND1A	HLA-A	RFC5	ZNF587
CPSF3	RGS1	ACTB	TTTY3	KYNU
KLHL6	ARL11	PSMB3	CCT5	CKS2
VPS29	SDC1	TMPRSS4	PNP	GBP5
AMPD3	ZNF101	ALYREF	KDF1	FOXA1
PSMC4	GNLY	FOXE1	CENPQ	CXCL10
REL	PSMB2	BMS1P6	RPL39L	KIF23
SKA1	POLR2H	DHFR	HIST1H3B	FDCSP
CYB5R1	TMPO	PSAT1	KIF4A	PLA2G7
HSPD1	THOC3	SNRPA1	RELB	STAG3
HIST1H2BK	MARVELD3	HIST1H2BE	HIST3H2A	TPX2
PNPLA3	CXorf65	EDARADD	HLA-K	KLHDC7B
HIST1H2BB	LMNB2	ARMC8	CD86	FANCI
TUBA1C	B3GNT3	ZNF683	EPPK1	PTTG1
ADGRG1	KIF15	KIF18A	S100A11	NETO2
GK	GRP	CBWD5	FZD6	CDC45
RNASEH2A	HIST2H2AC	GPR68	NDC80	KIF18B
DDX39A	PMAIP1	CYTIP	LDHA	KRT7
BIRC3	SLC31A2	RBP1	MTHFD2	CLDN1
DSC3	MX2	IL32	NMB	IGLL5
PGK1	C2	FOXD1	MMP3	RDH10
CDC7	PARP9	CD19	SLC17A8	GABRP
LOC100128840	RCC2	HS6ST2	ENO1	IFI44L
SNRPB	TNFAIP2	CHAF1A	TNFRSF12A	LAMP3
ACTL6A	POLQ	UHRF1	NCAPG	GPR87
NPL	DCUN1D5	TPM3	NCAPG2	RFC4
IFI44	DENND1C	DEPDC1	MTHFD1	C1orf106
FXYD3	CXCL13	MMP21	NFE2L3	AIM2
CHST11	C1orf112	IRF6	KIF20A	EZH2
HSPA6	EPSTI1	LINC00152	PHF19	CDT1
H2AFV	FAAP24	HIST1H2BM	SGO1	BIRC5
LIG1	RASSF4	PSME2	HELLS	PCSK9
HENMT1	CKAP2	CARNMT1	BATF	CENPF
NPVF	CD53	HIST1H2BI	LAP3	CENPU
BST2	FEN1	MCM3	TIPIN	KIAA0101
BBC3	ACOT7	CD300A	CCL18	CA2
DEFA6	FCGR3B	TIGAR	MX1	CDK1
C12orf54	SPOCD1	TUBB3	SPRY3	IFI30
PROSER2	MGST1	HRK	CIITA	PLAU
PTPRC	COL10A1	MIR4435-2HG	IL23A	ECT2
PLOD2	RMI1	CCNE1	PRIM1	NEFH
ZC3H12D	VEGFA	TNFRSF21	CDKN2D	STMN1
RAB3IP	PCNA	MRPL47	HAVCR2	WDR66
GNB4	LGALS8	ARNTL2	HIST2H3A///HIST2H3C	PIM2
MAGOH	PARP1	HLA-G	HCAR3	LOC100506100
SERPINB5	KRT19	G0S2	BMS1P20	CDH3
HIST1H2BG	CD96	E2F1	DHCR24	HIST2H2AB

DERL1	DONSON	SLAMF8	ADM	TNFSF10
UGT1A6	UBE2S	MOCOS	GZMB	CCR7
GMPS	HILPDA	NFKBIE	NUSAP1	SMC4
LILRB3	ADAM8	CCDC150	LYPD1	CCNB1
TRAF3	HMGB3P1	GLDC	SULF1	PRC1
MCM10	KNSTRN	B2M	APP	CD70
HIST1H2BF	HK2	FAM49B	SPC25	PTTG2
DNAJC9	MCM5	GRHL2	UBALD2	STAT1
RECQL4	IL4I1	DSN1	DTL	TRIM59
ADAT1	TMEM132A	TFRC	SNX5	TYMP
HIST1H2BN	RIPK4	SLC20A1	CTSZ	RRM2
CENPE	PLSCR1	HIST1H2BL	RABL6	APOL6
C20orf24	TCL1A	TNFRSF11B	GBP4	NUF2
C1QB	PPT1	COL8A1	LAMC2	SYCP2
SLC25A5	IGHV3-48///IGHV3-69-1	CTAG1A	IL36G	ICAM1
DUSP7	AMMECR1L	KIF2C	PKMYT1	ANLN
IRF7	NAMPT	TACSTD2	EGLN3	S100A2
SLC16A3	GBP1	HIST1H2BH	LTB	HIST2H2AA3
IFT80	PTAFR	PHLDA2	HIST2H2BD	UBE2T
FAM24A	CEP152	ADGRE1	H2AFZ	TYMS
BUB1	ITGA3	HMGB3	MCM2	MMP9
STK17A	NCAPH	ZC3H8	LEMD1	EFNA1
CREB3L2	CXCL1	BRI3BP	BTN2A2	PLK1
PBK	KPNA2	CCL19	GGH	TFEC
MARCH5	HLA-C	RAB4B	LYZ	CTSS
EBP	ABHD2	GINS2	MCM4	USP18
CKS1B	MCM7	BCKDHB	FANCA	NEK2
SLAMF7	ISG15	SNX10	FCGR1B	RAD54L
RCCD1	IFI16	MAD2L1	OLR1	HAGLROS
IL21R	BLM	HIST1H2AD	HMGB2	SPAG5
KNL1	SLC2A1	HMMR	CDCA2	HIST1H1C
ANXA3	HIST1H1D	FAM110A	BPNT1	IL2RA
SAT1	HIST1H1E	MMP10	CDC6	SPP1
ATAD2	KRT17	ADAMDEC1	CDCA8	E2F7
COTL1	PSMB9	INHBA	CXCL11	TCAM1P
TAP1	MELK	FAM20C	RACGAP1	DBF4
GMNN	OASL	UCK2	GDF15	FAM64A
TTK	CENPK	HN1	MEI1	LY6K
MLF1	OIP5	ELF3	IGHM	TK1
NCF2	MCM6	MB21D1	FCGR3A	IDO1
IFI27	STYK1	TIMELESS	CDKN3	CHAF1B
NMI	CDKN2B	FAM83D	LMNB1	MMP12
GALNT14	TICRR	DLGAP5	CEP55	APOC1
RAD51AP1	CENPW	EXO1	ASPM	EDN2
TRIB3	FAM26F	HIST1H2AE	CENPN	MMP1
KIF11	PATJ	WDHD1	CENPA	CXCL9
CCNB2	FANCD2	S100P	CXCL8	UBD
KRTCAP3	KNTC1	RAD51	SOD2	CDKN2A
RRM1	HTATIP2			
