# Supplementary material for: Identification of Prognostic miRNA Signature and Lymph Node Metastasis-Related Key Genes in Cervical Cancer
Source: Front Pharmacol. 2020 May 8;11:544. doi: 10.3389/fphar.2020.00544 (PMC7226536; doi:10.3389/fphar.2020.00544)
Supplement: Supplementary file 6 [file Table_4.pdf]

Table S4. The target genes of four prognostic miRNAs from the prediction intersection between TargetScan and miRDB.

**miR-502**				
KCTD9	KCNA1	ZFX	TENM2	SFMBT2
ZFHX4	COL10A1	DCLK1	SMIM13	MIER3
SEPSECS	SAMD12	PROM2	CCDC50	ACTBL2
NAP1L5	SCN2A	STK10	SEC14L4	STK16
DAPK1	UBE2H	TC2N	KRAS	NHLRC1
CSDE1	ANP32E	NRAS	TFDP2	PABPC1
ADAMTS3	PPP2R2C	DCUN1D5	ARHGEF15	ESCO1
CBLL1	KDM4A	TMEM65	DOK6	TMEM110
SEC63	SNX13	GCNT4	MLLT6	C7orf60
FBN2	PCDH9	TMEM151B	PHOX2B	GADD45A
TSHZ3	EML6	PEG10	SCN3A	TXNL4A
ELAVL2	MYNN	NCOA4	CHODL	AKT3
GAN	KIAA1522	ZRANB2	NOS1AP	DONSON
ZMYM4	FAM126B	EXOC5	AHR	GTF2A2
RBMS1	AFF4	JDP2	FILIP1L	DNAJC14
PPP2R5E	ASPH	PAFAH1B1	TRIP12	FIGN
SEMA3C	RCC2	WDR33	PRDM6	YTHDF3
SREK1	PSD3	PRPH2	CALU	SLC9A6
STIM2	PPP3CA	CDH5	RAB1B	ODF4
ZBTB43	SUMF1	TJP1	KITLG	SRPK2
CDK6	RCHY1	TMEM178B	SRPR	RAB6B
BCL7A	SHPRH	FUNDC2	DOT1L	IQCK
ADCY2	DCC	USP37	FAM120C	PLSCR2
RBM41	DLGAP2	CLIC4	NDUFV3	MYPN
TET3	RPRD1B	B4GALT5	RBAK	ZNF365
TPK1	SSR3	SBNO1	RFX3	LCA5
KPNA4	RNF144A	NQO1	EXTL2	EAF1
AKAP3	EIF2S2	ACSS3	NKX3-1	MRPL35
SVOP	MEGF11	MVB12B	MAPK8IP2	ADAMTS18
HNRNPA2B1	PRAMEF1	TET1	MARC2	NAA60
EIF4G2	SLC28A3	SAMD4B	SLC36A3	NAALAD2
DAZAP2	KANSL2	AICDA	KIAA0930	TBC1D10C
TOB1	KIAA1549L	C12orf54	PLEKHA6	CST3
TMEM127	APOBR	CCSAP	RPTN	SV2B
ADAM12	TNFAIP8L1	ZNF528	ARCN1	SZT2
SPTY2D1	CCR6	SLIT3	C2orf88	SYNPO2
INSIG1	SLC39A14	DEFB132	HOXA3	DAZL
CHST11	EPHA4	H1F0	CEP97	PSAPL1
ZNF471	AUTS2	DLST	LRP6	ZNF829
FRMD4A	HELZ2	COMMD9	C21orf62	AIFM1
KLK2	PRMT2	LDHD	PKP1	FAM19A1
GALNT4	HAUS6	CDC20B	WDHD1	CDKL2
QSOX2	FAM172A	SPRED2	ATP11A	PTPRT
SORBS2	FAM46A	OSBPL10	DAZ4	MREG
BABAM1	GOLGA7B	CLSTN2	CDK19	PHC1

POC1B-GALNT4	TOR1AIP2	SLC15A4	DAZ2	NUDT10
SYT4	SMEK2	SLC7A14	DAZ1	FBXO28
TFE3	ZNF709	CNTN2	DAZ3	AMIGO2
ADAMTS17	SYT9	PGM5	ATP1A2	RPRD2
B3GALT5	IDH3B	ICMT	CDK14	SGPL1
LUZP1	C16orf91	HS6ST2	NCOR2	FUT3
FOXN2	RLIM	MSL3	LAMA3	SUPT7L
ARSD	PRAMEF13	TLR6	PLXNA4	PHYHIPL
TRPM8	GLP1R			
**miR-145**				
ATRX	COPG1	CNTN5	NCOA7	FAM135A
CCDC88A	AHCYL2	TRPC5	HNRNPUL2	ANGPT2
TTBK2	RAPGEF4	CCDC40	NUGGC	GABARAPL2
RAP2C	PCGF5	MZT1	ODF2	MYO5A
ZNF268	ITGBL1	ZNF181	LACE1	ELK4
SHOC2	SLC1A2	PAX8	KANSL3	PRKX
NFYC	SHB	STRN4	ARL5B	UBA6
PBX3	RLIM	PTGFRN	ZFP1	CLCN3
SLC30A5	OGT	CEP63	PSMA1	SLC38A11
CYP51A1	TCTN3	LPCAT3	ZFP30	BACH2
DDX3X	ZFP14	HTR1F	CCDC141	MKL2
PCDH19	EGLN1	CSMD2	ZNF879	ZFHX4
SLC24A2	CD1D	LRRC2	TANGO2	ELMO1
RBPJ	PPP6C	ARMC3	AXIN2	NET1
KDM3A	TPH1	CRLF3	GFI1	ANKRD28
SMG7	ANO6	NDUFS1	NPNT	EXOC8
NUFIP2	TUFT1	CTPS2	MPZL2	TRIM2
FAM168A	DLX3	SOX12	DAB2	SERINC5
CNOT6L	MINPP1	ADARB1	KCNA4	ZFYVE9
CCNC	CDC14A	STK24	AP1G1	KATNBL1
STK17B	ZNF75D	BMS1	YTHDF2	AKAP9
MPP3	NR6A1	PSD3	SPSB4	ACTG1
HTRA1	UNC119B	NDUFA10	ATP8A1	CSMD3
LRP10	FSD1L	KCNK10	SEMA3A	MYO1D
EAF1	IVL	BVES	ABHD17B	HEPHL1
ZMYND11	ELOVL6	DGKB	DOCK9	DUSP6
DDX6	MYO7A	NCOA1	KLF5	MDFIC
ANO5	KLHL3	LRP6	CACHD1	KIAA0930
MIS18BP1	SNX2	CS	NUAK1	RNF170
LIX1	TBL1XR1	SOX6	ST6GALNAC3	RREB1
ZC3H7B	JAKMIP2	SPARC	SEMA6A	SNX27
GRIK2	LRRTM1	CCDC71L	SRGAP2	TMEM167A
EDEM3	ZNF687	EFR3B	LOX	BRCC3
SLC8A3	DPP10	MGLL	INO80	ZBTB10
LCORL	MOCS2	LCOR	ATXN2	TBC1D15
EBF1	SERPINA1	YPEL2	REV3L	PLCE1
PDZK1	SET	CTSO	FSCN1	RTKN
YWHAQ	MMP14	STAM	SRGAP1	MEST
NAA30	CCSER1	FRS2	CAMSAP2	RASA1

TFE3	AGA	CDC27	SNX8	ARHGAP6
SIX4	DAAM1	TMLHE	FLI1	KCNK1
THSD7B	PIGA	ABRACL	IVNS1ABP	TDRD6
EPS15	PLCL2	ADCYAP1	GAREM	GPR155
NEDD9	TNFRSF11B	DYRK1A	CDK14	CCDC25
CRKL	ARHGAP28	SH2B3	ARHGAP24	FBXO34
HNRNPH2	ERLIN1	FBXL4	UBN2	AREL1
PXN	SMAD3	NR4A2	TMEM56	CAMK1D
FNDC3A	MYO6	FGD6	SLC7A8	SMIM12
SIRPA	RFX3	AKAP12	CRTC1	ACSL4
RNF216	MMP16	CREB3L2	UXS1	FAXC
FLNB	SMAD5	PAK7	XRN1	SHISA9
ZFYVE26	RBM22	DPH5	ITPRIPL2	FNDC3B
ADAM19	RBM20	AP3S1	RC3H1	GTPBP8
GGT7	USP31	PAPD5	HS6ST1	TENM1
TPM3	PTGFR	LLPH	PDGFD	KCNA1
FAM174B	ABR	KIF21A	TMED7	TULP4
RAB14	DDC	LENG8	ERF	SMAD2
GPBP1	OS9	SNED1	GRB10	DPY19L4
RGS7	TLN2	ZC3H11A	EYA3	SPTLC2
DLG4	DENND4B	H2AFX	NTN4	GXYLT1
PCSK5	CLOCK	SLC25A36	RBPMS2	KLHL28
EPB41L5	PPP3CA	CTNNBIP1	ACBD3	SLITRK4
SLC25A25	AARS	TM9SF4	PAQR9	PHACTR2
SBF2	MAP4K4	NAP1L1	ERG	ADRA2B
RAD51B	EEA1	SNX15	APAF1	GRIK4
LRAT	CPSF6	SORCS1	C5AR1	CAPZB
EBF3	SPATS2	CSTF3	MAP3K3	HTR2A
HECW2	MED13	CLINT1	TAT	SLC24A4
HIC2	NSUN4	NRAS	CDC37L1	TARSL2
ARIH1	NXPE3	IRS1	ZBTB33	PVRL3
SKP1	GFRA1	KCNA6	CDR2L	MTF1
TNFRSF10A	RAPH1	CLCN5	BCAT1	CADM2
GLIS1	OSBPL1A	CPEB1	GPR137C	THPO
ARPC5	SLITRK6	CITED2	SMIM15	EPHA6
PAFAH1B2	LMBRD2	LRRC16A	PLAGL2	PPIP5K2
ADPGK	MBTD1	JPH1	FBXL3	VPS41
PRKD3	SPOP	MAGI2	TAGLN2	CHRNB2
YTHDC1	ARAP2	TGFBR2	PPP3R2	FREM1
ACTB	ZNF423	FBXO28	DENND5B	ZNF436
PAN2	FOXO1	EYS	CCNL1	RNF207
KDM2B	USP46	ADAM17	RAB11FIP4	TBC1D14
ABL2	SH3BP1	CNDP2	EIF4EBP2	GOPC
CSRNP2	QSER1	ZRANB3	SNTB2	USP3
NAA50	SCAMP3	KDM6A	GATC	RRP7A
NUS1	RAD52	HOMER2		
**miR-142**				
FAM208B	SGMS1	GTF2A1	IER3	SETD2
WASL	PLCB1	ZMYND8	CASK	PRPF40A

HECTD1	MYH10	ADAMTS3	RFWD3	FAM91A1
RLF	RIMKLB	RARG	RPRD1A	ROBO1
USP6NL	LRP1B	GFI1	HMGA1	FAM199X
C9orf72	CLOCK	AFF2	SP8	CNEP1R1
TWF1	CLTA	APC	LLGL2	STC1
C20orf194	MBD6	LCOR	ZFPM2	HIPK1
BAZ1A	ZNF217	FNDC3A	BAI3	ZCCHC11
EML4	FBXO3	GOLGA1	AFF4	ICK
FMNL2	RHEB	SLC7A11	DIAPH2	APPBP2
C5orf24	HSPE1-MOB4	ZCCHC14	C11orf87	WWP1
RICTOR	MARCKS	RBM47	CDK17	SIX4
STAU1	PSMB5	RAB3A	IGF2BP3	REV3L
S1PR3	KDM6A	RBFOX2	ADAMTS1	FAM63B
MORF4L2	ASH1L	AKT1S1	CBLN4	SACS
FOXO4	SNX18	XPO1	KITLG	MOSPD2
LRRC1	BOD1	MGAT4A	ATXN7L2	ZNF503
DCUN1D4	ARL15	SLC37A3	GOPC	UBE2D1
RERE	BNC2	HGS	ZNF770	ARRDC3
OSBPL3	TGFBR1	CPEB2	LMX1A	NCKAP1
KIF5B	FAM114A1	TNKS	PTEN	BTBD7
STRN3	UTRN	ATXN1L	RAP1A	ARID2
ZBTB41	AFF1	SUCO	RAB6B	TRIM36
TMEM59	STX12	PFKM	VPS54	DNAJC25
STAM	SYPL1	CFL2	PRPF4B	MED14
RHOBTB3	ANK3	SLC12A9	BOD1L1	RSF1
TMEM200B	TAB2	FAM46A	LHFPL3	NFE2L2
INPP5A	MRFAP1	FYCO1	GRSF1	CAMSAP2
ARNTL	TBL1X	COG4	RERG	PKN2
BRWD3	ROCK2	KDELR2	SLAIN1	MAGI2
RGL2	SGK1	TEAD1	GAS7	DIO2
SMG1	PCGF3	MAP3K11	PTP4A1	FEM1C
SLC35F5	TNRC18	TIRAP	BMPR1A	FAM168A
IPMK	EGR2	REPS2	STAG1	PRKG1
MLXIP	SIK1	BACH2	KLF10	TMF1
ZEB2	HMGB1	HEATR5A	LRP12	ABCA1
SLC38A2	TRPC4	DUSP2	TNKS2	SP2
CUL4A	CDK19	SCN2A	BECN1	DIAPH3
ELAVL4	BTBD1	OTUD4	PRNP	CIT
FIGN	FAM19A1	ATG16L1	GDNF	UCHL3
HERPUD1	KIAA2022	SAMD8	EMB	RGPD6
IL6ST	PUM2	SMURF1	SGCE	TLE4
ULK1	BRD1	DNAJC7	GCNT1	RGPD4
ALS2	USP9X	RNF180	SELT	ACIN1
SPAG9	JMJD1C	FBXL3	KRR1	GCC2
NR2C1	RHOT1	HSPA13	HSD11B1	LPP
ARHGEF12	BMPR2	EIF4E3	DDHD1	GATA3
FMR1	BNIP2	CXorf23	NECAB1	SLC5A3
PLD1	HECA	IQCJ-SCHIP1	HLTF	RHOBTB1
NPAT	VGLL4	ANKRD29	STK35	SLC30A7
MED13	RALGPS2	KRAS	PLXNA2	PHIP

TRPC4AP	ARHGEF6	CAPN10	SSH1	HMGCLL1
ATP1B1	CNTN1	MED12L	FAM107B	TWISTNB
CUL2	RHOC	MAN1A1	FBXO30	MSL2
SORBS1	LUZP2	TMEM65	NBEA	CHD9
TCEB1	CEP97	FGG	SPSB1	LY75
FAM134B	BVES	PGRMC2	TMTC1	NPAS4
RNF128	TCF21	FAM92A1	DNAJB4	KLHL14
ZBTB43	EFCAB14	ST6GALNAC3	PDLIM5	SGCD
TSC22D2	STK38L	FCHO2	EGLN3	ACADSB
RAB11FIP5	MYO1D	BAZ2A	VPS26A	WDR26
HDLBP	TP53BP2	PPM1G	CDK5	CADM2
FAM131B	RPS6KA5	DCBLD2	PBX3	TRIM23
ZBTB37	NPNT	CPSF6	SREBF1	GPR75
CCNG2	HMGCS1	TRIP11	BTBD10	CD69
PDS5B	EYA4	SPEN	RFX3	MYT1L
SYNJ1	RCN2	APBB3	PVRL3	TMEM38B
MBD2	LEPROT	FRMD5	CELF5	CACNA1E
SLC26A3	SEL1L3	PCBP2	TOX	MASTL
RC3H1	SLC2A13	ZFYVE26	TIAM1	TGFBR2
NFAT5	MGAT4B	TBC1D9	PHF6	SLC24A2
MIER3	CREBRF	RRM2B	IGF1	SOX5
PTBP3	NCK2	UBN2	GK	RTN1
**miR-33b**				
FEZF1	BICD2	PLD1	MYOCD	ALDH1A3
TIGD2	PTPRG	RNF43	ZNF24	EPB41
CLOCK	MAPK10	DAZAP2	PKD1L1	TTLL1
ZKSCAN1	TRAK1	MYO5A	ARHGEF12	NAA30
IGSF10	RCBTB2	ATG14	TSPAN1	GALE
LRP8	TMEM2	AKAP6	MCF2L	TAC4
EPHA7	YPEL4	MSR1	DNAJC11	GAPT
FAT2	DLL1	ZFP3	DENND1B	SERPINA1
CYB561D1	CBLL1	PRAMEF16	GABBR2	ZSCAN20
NPAS2	ATP1A2	DLG2	RTN4	BCL11B
MYO6	SAMD5	LYRM2	TBC1D2	CS
AGO1	HMGB3	CACNB4	USP46	CERS5
PAFAH1B2	TRAF3IP1	MTRF1L	FGF1	MPV17
DCUN1D3	CADM1	PRAMEF17	SPRYD7	LMO2
MARCH8	HSP90AA1	C5AR2	PTBP3	MBOAT2
FRMD4B	CMTR2	ZFYVE26	C11orf87	SCNM1
SSFA2	MKL2	RBFOX1	SNN	MON2
SYT1	C6orf222	TLK2	DCAF12	ZKSCAN8
ARIH1	KIAA1549L	TMX3	KIAA1432	STK4
CSRNP3	ZNRF3	CXorf23	TLN2	HAUS4
ZNF513	CRYZL1	DHX15	BAZ1B	SHISA2
DOCK4	GIGYF1	SMARCAD1	NANOS1	ARL4A
URI1	RIMKLA	NFIA	ARL1	DYRK1A
PRKAR1A	TMEM39A	FAM212B	VAPA	LHX6
PLBD1	VDAC1	ABCD3	MTMR4	NR1D2
ACBD3	EGR4	HSF5	CRK	PHC1

ZFAND5	PAG1	RBM12B	GCNT4	PTPRB
ZNF202	BCOR	TENM3	XRN1	FAM134A
KIAA1715	KLHL31	ANKRD29	CSMD1	MAP4K4
SS18L1	RABGAP1	MAPK8	GPR88	RFX3
UBA7	TMEM194B	ANKRD44	ZMIZ1	NDUFA10
PRPF40A	SORBS2	PIM1	CPT1A	SEPSECS
SV2B	MED31	CCNY	IRS2	NPC1
SLC6A15	MKI67	ABHD2	SELT	SLC14A1
CTSV	SLCO3A1	SLU7	SIRT6	EEF1A1
CXCL12	MBL2	PPTC7	SNRK	SECISBP2L
KCNJ13	TMEM218	SETD7	CHD1	EGFLAM
MLLT3	MKRN1	ENC1	RASSF8	BTBD2
ERI3	GPR123	UBE2V2	PTGFRN	TFDP2
C10orf128	NTM	PRICKLE2	LUC7L3	ESCO1
GPR155	PFN4	UNC79	HMGA2	ZCCHC14
ACSM2A	ZNF185	GPCPD1	PCDH18	SDC3
RHD	SFR1	NIPAL4	USP32	RNF180
SUZ12	BRD4	FAM46C	ADAM12	TMEM236
GPR173	TRIM32	KPNA4	GDNF	TLE3
PTGES3L	ABCA1	FBXO47	FBXO33	PTPRC
NR4A2	HADHB	SEMA7A	ARMC8	XBP1
FAM188A	CROT	CERK	CPEB3	MAP3K7
RORA	SCN8A	TM9SF3	KPNA3	PIM3
PTPN3	CDK6	DSC3	RNF128	TANC2
STAMBP	SEC24C	PHF12	TSPAN9	RASEF
MBD1	CACNA1C	STON1	SREK1	CLPX
DCTN4	YWHAH	HIPK1	MMP16	CSNK1D
SAMD12	SLC25A25	CADM2	ST18	METTL21A
BORA	ZNF281	MDM4	CNTN4	SAMD4A
TRIM9	MLXIP	RPS6KA3	PDE7A	
