# Supplementary material for: Identification of Prognostic miRNA Signature and Lymph Node Metastasis-Related Key Genes in Cervical Cancer
Source: Front Pharmacol. 2020 May 8;11:544. doi: 10.3389/fphar.2020.00544 (PMC7226536; doi:10.3389/fphar.2020.00544)
Supplement: Supplementary file 7 [file Table_5.pdf]

Table S5. The enrichment analysis of KEGG pathways and GO function annotations.

Term	Genes	Count	PValue
GO-CC plasma membrane	SLC2A13, HDLBP, PLXNA4, NBEA, KCNA6, PAQR9, TLR6, **CDH5**, TNFRSF11B, DGKB, HTRA1, SEMA7A, RAPGEF4, HTR1F, ACTB, GPR173, **PTPRC**, **IGF1**, MMP16, ATP1A2, DAPK1, RCC2, BNC2, DSC3, CNTN2, LRP8, CACNA1C, GLP1R	28	0.04327666
GO-CC integral component of plasma membrane	PTPRB, **PTPRC**, SLC2A13, PLXNA4, KCNA6, MMP16, TLR6, SLC7A14, SEMA6A, TNFRSF11B, TENM3, CNTN2, HS6ST1, DCLK1, HTR1F	15	0.00691935
GO-BP cell adhesion	LAMA3, PGM5, CNTN2, DSC3, **CXCL12**, **CDH5**, ITGBL1	7	0.03033652
KEGG Pathway cAMP signaling pathway	CREB3L2, RAPGEF4, ATP1A2, CACNA1C, GLP1R, HTR1F	6	0.00386967
GO-CC proteinaceous extracellular matrix	TNFRSF11B, MMP16, FBN2, COL10A1, SLIT3	5	0.03943575
GO-CC postsynaptic density	DLGAP2, PDLIM5, LRP8, CACNA1C, DCLK1	5	0.01168554
KEGG Pathway Adrenergic signaling in cardiomyocytes	CREB3L2, RAPGEF4, ATP1A2, CACNA1C, TPM3	5	0.00612939
GO-BP neuron migration	PHOX2B, SEMA6A, CNTN2, **CXCL12**, DCLK1	5	0.00206901
KEGG Pathway Insulin secretion	CREB3L2, RAPGEF4, ATP1A2, CACNA1C, GLP1R	5	0.00105101
GO-BP axon guidance	SEMA6A, CNTN2, **CXCL12**, SLIT3	4	0.04822608
KEGG Pathway Axon guidance	SEMA6A, SEMA7A, **CXCL12**, SLIT3	4	0.0302569
KEGG Pathway Leukocyte transendothelial migration	ACTB, RAPGEF4, **CXCL12**, **CDH5**	4	0.02341206
KEGG Pathway Dilated cardiomyopathy	ACTB, **IGF1**, CACNA1C, TPM3	4	0.0101214
KEGG Pathway Hypertrophic cardiomyopathy (HCM)	ACTB, **IGF1**, CACNA1C, TPM3	4	0.00826116
GO-BP extracellular matrix disassembly	LAMA3, HTRA1, MMP16, FBN2	4	0.00696678
GO-BP regulation of insulin secretion	RAPGEF4, CACNA1C, GLP1R	3	0.04625611
GO-BP adult locomotory behavior	ATP1A2, GDNF, **CXCL12**	3	0.02913473
GO-BP neural crest cell migration	SEMA6A, SEMA7A, GDNF	3	0.02225771
GO-BP negative chemotaxis	SEMA6A, SEMA7A, SLIT3	3	0.01307806
GO-BP semaphorin-plexin signaling pathway	SEMA6A, PLXNA4, SEMA7A	3	0.01234947
GO-BP positive regulation of G2/M transition of mitotic cell cycle	PHOX2B, **RAD51B**, RCC2	3	0.0037608
GO-BP sympathetic nervous system development	PHOX2B, PLXNA4, GDNF	3	0.0026068
GO-BP positive regulation of dopamine secretion	GDNF, **CXCL12**	2	0.03034261
GO-BP heparan sulfate proteoglycan biosynthetic process, enzymatic modification	HS6ST2, HS6ST1	2	0.02534946
GO-MF heparan sulfate 6-O-sulfotransferase activity	HS6ST2, HS6ST1	2	0.01975771
GO-BP postganglionic parasympathetic fiber development	PLXNA4, GDNF	2	0.01021705
